# Dietary intake of total vegetable, fruit, cereal, soluble and insoluble fiber and risk of all-cause, cardiovascular, and cancer mortality: systematic review and dose–response meta-analysis of prospective cohort studies

**DOI:** 10.3389/fnut.2023.1153165

**Published:** 2023-10-03

**Authors:** Feifei Yao, Jianping Ma, Yong Cui, Cuihong Huang, Ruiqi Lu, Fulan Hu, Xiaoming Zhu, Pei Qin

**Affiliations:** ^1^Clinical Public Health Center, Shenzhen Qianhai Shekou Free Trade Zone Hospital, Shenzhen, Guangdong, China; ^2^Shenzhen Qianhai Shekou Free Trade Zone Hospital, Shenzhen, Guangdong, China; ^3^Department of Oncology, Shenzhen Qianhai Shekou Free Trade Zone Hospital, Shenzhen, Guangdong, China; ^4^School of Public Health, Sun Yat-sen University, Guangzhou, Guangdong, China; ^5^School of Public Health, Shantou University, Shantou, Guangdong, China; ^6^Department of Biostatistics and Epidemiology, Shenzhen University Health Science Center, School of Public Health, Shenzhen, Guangdong, China; ^7^Department of Biostatistics and Epidemiology, School of Public Health, Xi'an Medical University, Xi'an, Shanxi, China

**Keywords:** dietary fiber intake, mortality, cancer, cardiovascular disease, meta-analysis

## Abstract

**Objectives:**

To conduct a systematic review and meta-analysis of prospective cohort studies to investigate the association between total, vegetable, fruit, cereal, soluble and insoluble fiber intake and risk of all causes, cardiovascular disease (CVD), and cancer mortality and quantitatively assess the dose–response relation.

**Methods:**

Eligible studies were identified by searching PubMed, Embase and Web of science before August 2023. Random effects models were used to calculate summary relative risk (RR) and 95% confidence intervals (CI) and restricted cubic splines to model the linear/non-linear association.

**Results:**

The summary RR for all-cause, CVD and cancer mortality of dietary fiber was 0.90 (95% CI: 0.86,0.93), 0.87 (0.84,0.91), 0.91 (0.88,0.93), respectively. Significant association was observed for all-cause and CVD mortality with fruit, vegetable cereal and soluble fiber intake and cancer mortality with cereal fiber intake. No significant association was found for insoluble fiber, vegetable or fruit fiber intake and cancer mortality. Dose-response analysis showed a significant non-linear relation of dietary fiber intake with all-cause mortality, and linear relation for others.

**Conclusions:**

Higher dietary fiber including different type and food sources of fiber intake were associated with lower risk of mortality. Our findings provide more comprehensive evidence on dietary fiber intake with mortality.

**Systematic review registration:**

https://www.crd.york.ac.uk/prospero, identifier: CRD42022338837.

## 1. Introduction

Cardiovascular disease (CVD) and cancer are the leading causes of death globally (1). It has been estimated that global deaths from coronary heart disease, stroke, and cancer will reach up to 18.6 million, 12.2 million, and 10.0 million, respectively in 2019–2020 (1–3). Poor diet contributed to one of the largest risk factors for death, accounting for 8.3% of all deaths (4). The WHO recommends a daily intake of dietary fiber >25 g/day for adults (5); however, the consumption of dietary fiber remains low in many high-income countries (18.3 g/day in the United States, 14.8 g/day in the United Kingdom, 16.9 g/day in France, and 15.0 g/day in Japan) (6). Major nutrition shifts occur in developing countries with an increase in fat intake and a decrease in whole grain and fiber intake. The dietary fiber consumption level was reported to be even lower in middle-income countries (9.7 g per capita/day in China) (7, 8). Accumulating evidence indicated that dietary fiber might decrease the risks of various chronic diseases (9, 10), including obesity, diabetes, hypertension, CVD (11–14), and cancer (15–17).

Inconsistent results were found in previous studies examining the effect of dietary fiber on mortality. Most of the previous studies detected an inverse association between dietary fiber and all-cause, CVD, or cancer mortality (18–20), but no association was found in other studies (21, 22). Although few systematic reviews and meta-analyses were conducted to analyze the relationship between fiber intake and mortality, some of those meta-analyses focused on specific populations such as patients and cancer survivors (23–25) and unstable findings have been reported with controversial results in many subgroups. A most recent meta-analysis conducted in 2019 analyzed the relationship between total fiber and a series of health outcomes, which included 68,183 deaths, but did not take into consideration the specific types of dietary fiber (26). More than 10 studies (18–20, 22, 27–33) have been reported since the last meta-analysis, with approximately 424,953 participants and 30,215 deaths that could be further added in this updated meta-analysis. Therefore, it is necessary to conduct an updated meta-analysis to explore the association between dietary fiber intake and all-cause, CVD, or cancer mortality and provide evidence on their dose–response relationship.

Dietary fiber can be classified into insoluble and soluble fibers based on solubility (34). Studies on associations between insoluble or soluble fiber intake and mortality have also been inconclusive. In a cohort study of 92,924 Japanese consumption of both insoluble and soluble fibers was associated with a lower risk of all-cause mortality (20). While some observational studies have not found a significant association between soluble or insoluble fiber intake and all-cause mortality mortality (28, 35). Only one previous systematic review and meta-analysis investigated the association between soluble and insoluble fiber intake and CVD mortality (36); however, the study did not assess the association between all-cause between all-cause mortality and cancer mortality.

The levels and sources of dietary fiber intake may be substantially different among countries. For example, grain products are the main source of dietary fiber for the US population (37), while dietary fiber mainly comes from vegetables for the Japanese population (38). Bean, fruit, and vegetable fibers but not cereal fibers are associated with reduced risk of all-cause mortality in a study conducted in Japan (39), whereas others reported no associations of individual food sources of dietary fiber (including fibers from cereals, fruits, or vegetables) with the risk of ischemic heart disease mortality mortality (40). Although a previous meta-analysis investigated the association between cereal fiber intake and all-cause, cardiovascular, and cancer mortality (41), the study included general participants and people with diseases, and several cohort studies with large sample sizes have been published in recent years (20, 28). Different from previous meta-analyses, this meta-analysis explored dietary fibers from different sources and cardiovascular or cancer mortality. To the best of our knowledge, most previous meta-analyses (24, 26, 36, 41–45) did not analyze the relationship between fibers from different sources and mortality. A meta-analysis (46) was conducted on the association between dietary fibers obtained from different sources including cereal, fruit, legume, and vegetable fibers and cardiovascular mortality.

Hence, our study aimed to conduct an updated systematic review and meta-analysis of prospective cohort studies to investigate the risk of all-cause, cardiovascular, and cancer mortality associated with dietary fiber intake and different food sources and different types (soluble and insoluble fiber) of dietary fiber intake in general populations and further explore the dose–response relationship.

## 2. Methods

The systematic review and meta-analysis were registered in the prospective register of systematic reviews database (PROSPERO) (https://www.crd.york.ac.uk/prospero/index.asp, identifier CRD42022338837) and conducted and reported according to the 2020 Preferred Reporting Items for Systematic Reviews and Meta-Analyses (PRISMA) guidelines (47).

### 2.1. Search strategy

We systematically searched the PubMed, Embase, and Web of Science electronic databases from their inception up to 25 August 2023. We used a combination of MeSH terms and free-text terms to identify relevant publications assessing dietary fiber intake and fibers from different food sources in relation to all-cause, CVD, and cancer mortality, with restriction to the English language and without date limitation. Moreover, the reference lists from the retrieved articles, systematic reviews, and meta-analyses were searched for further relevant studies. Study authors were contacted, but non-peer-reviewed sources were not considered. Details of the search terms used for querying literature are shown in Supplementary Table 1. The literature search was conducted by two independent investigators (F. Y. and P. Q.).

### 2.2. Inclusion and exclusion criteria

The PICOS (participants, interventions/exposures, comparators, outcomes, and study design) criteria were used to identify studies that were eligible for inclusion: (1) the study design was prospective cohort studies; (2) the exposure of interest was dietary fiber intake; (3) the outcome of interest included all-cause, CVD, or cancer mortality; and (4) the risk estimates, including adjusted hazard ratios (HR) or risk ratios (RR), with their corresponding 95% confidence intervals (CIs) were reported. When reports pertained to overlapping participants, we included only the study with a larger population to avoid duplication of data.

Reviews, abstracts, comments, or unpublished results were excluded. Studies on children, adolescents, or patients with chronic kidney disease, or who were undergoing hemodialysis, end-stage cancer, or critical illnesses were excluded.

### 2.3. Data extraction and quality assessment

The data extraction and quality assessment were conducted by F. Y. and P. Q., and any discrepancies were discussed with a third investigator (C. H.). The following characteristics from each study finally included in the meta-analysis were extracted using a standardized form: name of first author, publication year, country or region, the name of the study, sample size and number of deaths, follow-up period, types of outcomes, gender, age, types of fibers, amount of intake, measurement of fiber, assessment of interested outcomes, RRs/HRs and 95% CIs, and variables adjusted for in the analysis. When separate risk estimates for men and women were available in a study, their RRs were combined using a fixed-effects model to generate a pooled risk estimate. For dose–response meta-analysis, the risk estimates should be provided for at least three quantitative categories of fiber intake.

We assessed study quality with the Newcastle–Ottawa Scale (NOS) for cohort studies (48). A maximum score of 1 for each question in the checklist can be awarded. Scores were calculated according to three major aspects: selection of participants, adjustment for confounders, and ascertainment of outcomes and nine questions. Scores of 0–2, 3–5, 5–7, and 7–9 were considered poor, fair, good, and high quality, respectively.

### 2.4. Statistical methods

For studies reporting HRs for fiber consumption, we assumed that the HR was approximately equal to the RR (49). The missing number of cases in each category was calculated by using the reported RRs/HRs and the number of total cases (50). The average or midpoint of each defined quartile was used for the dose amount. If the category dose range was open-ended, we assumed the length of the open-ended interval to be the same as that of the adjacent interval. For studies reporting risk estimates compared to medium or highest dietary fiber intake, the RR was recalculated by setting the lowest category of dietary fiber intake as the reference.

We computed the highest vs. lowest estimates by using a random-effects model (51), which considered variations (heterogeneities) both within and between studies. We calculated summary RRs (95% CIs) of all-cause, CVD, and cancer mortality per 10 g/day increment. We used the generalized least squares regression to estimate study-specific dose–response associations (52) and the random-effects model to pool the study-specific dose–response RR estimates (51). To examine possible linear or non-linear associations, we used restricted cubic splines for each study with more than three categories of exposure, with three fixed knots at 25%, 50%, and 75% of the total distribution of the reported intake, and combined them using multivariable meta-analysis (53). The significance of non-linearity was calculated using null hypothesis testing (53). We combined the study-specific slopes using random-effects models.

Heterogeneity was assessed using Cochran's *Q* test and *I*^2^ statistic (54), with a value of *I*^2^ > 50% considered to represent potentially important heterogeneity, and *P* < 0.1 was considered statistically significant for the Q statistic (55). Publication bias was assessed using Egger's test and funnel plots. When Egger's test indicated bias, a trim and fill method was used to detect the effect of probable missing studies on the overall effect. We further carried out subgroup analyses stratified by study characteristics, including duration of follow-up (>10 vs. ≤10), number of cases (≤1,000 vs. >1,000), geographical location, study quality (>7 vs. ≤7), adjustment for confounding factors (physical activity (PA), comorbidity at baseline, carbohydrate, protein), and dietary assessment methods, and meta-regression to investigate potential sources of heterogeneity. We also conducted sensitivity analyses excluding each study at a time from each analysis to clarify if the results are robust. A two-tailed P < 0.05 was considered significant. The Stata version 15.0 software (Stata Corp., TX) was used for the analyses.

## 3. Results

**Figure 1:**
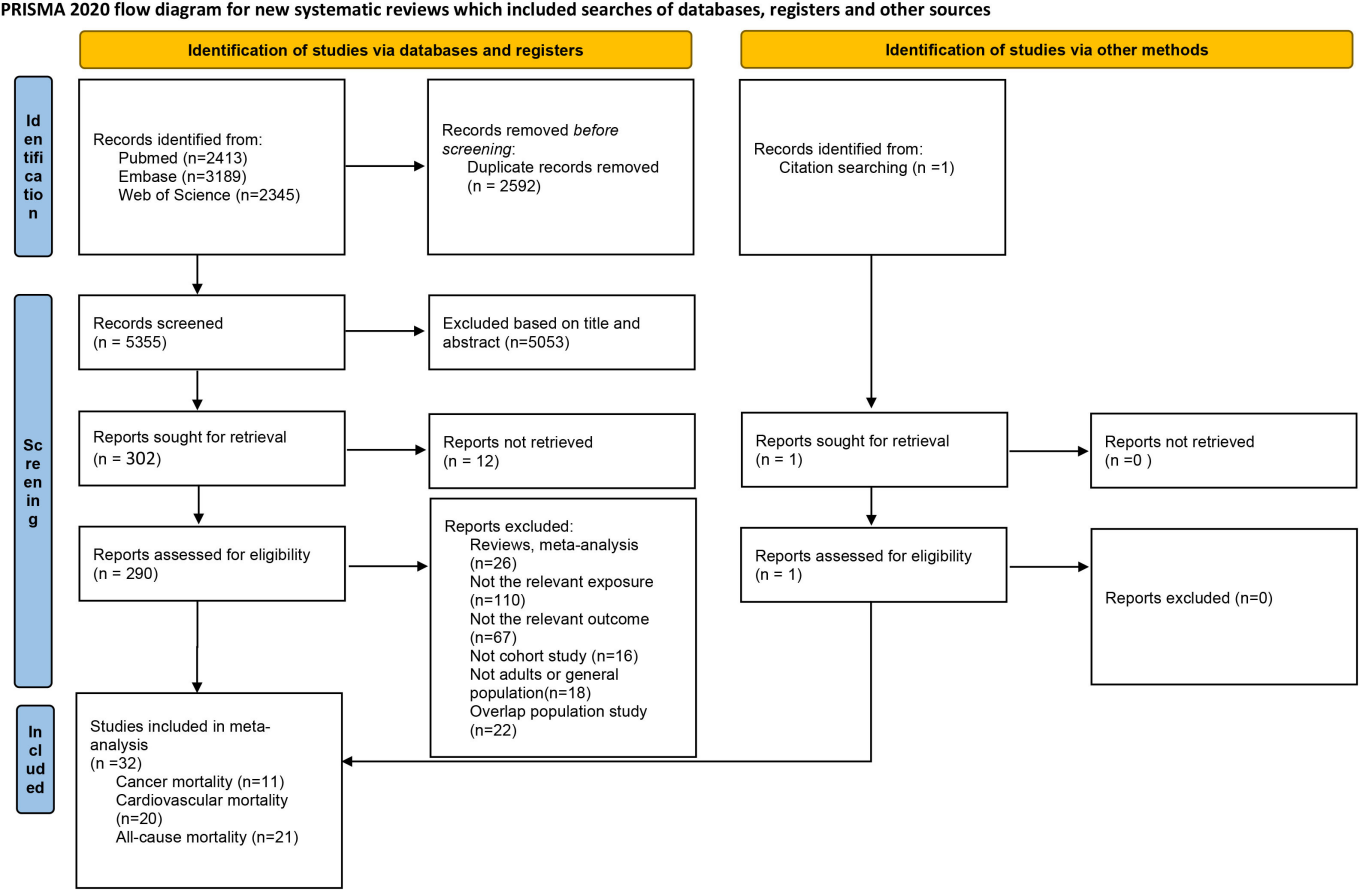
Flowchart of study selection.

The flowchart for the selection is presented in Figure 1. We found 7,947 studies through the database search and reference lists. After removing duplicates, 5,355 records remained. After reviewing the title and abstract of these studies, 5,053 studies were subsequently excluded, and 302 full-text studies were then assessed. After full-text screening, a total of 290 publications were excluded because of duplicated data from the same cohort studies (*n* = 22), reviews (*n* = 11), or meta-analyses (*n* = 15), not relevant exposure (*n* = 110), not relevant outcome (*n* = 67), not cohort study (*n* = 16), or not adults or general population (*n* = 18). Finally, 32 publications were included in the systematic review and meta-analysis.

### 3.1. Study characteristics

A total of 32 articles (18–22, 28, 29, 32, 35, 40, 56–77) were included in the systematic review and the present meta-analysis. The characteristics of the studies included in the meta-analysis are listed in Table 1. The number of participants in these studies ranged from 314 to 452,717, with a mean or median age ranging from 16 to 99 years. Ten studies were from the United States (18, 21, 35, 56, 62, 64, 65, 67, 72, 77), four from the United Kingdom (19, 70, 71, 74), three cohorts conducted in Australia (58, 61, 63), two conducted among multiple nations (40, 59), two from Spain (28, 57), one from Dutch (69), one from Finland (68), one from France (22), one from Israeli (66), three from Japan (20, 29, 60), one from Korea (32), one from China (75), one from Malaysia (76), and one from Sweden (73). The follow-up period ranged from 2 to 40 years. Notably, 22 studies assessed dietary fiber intake using the food frequency questionnaire (FFQ) (20, 28, 32, 40, 56–63, 65–67, 70–74), and 10 using 24-h dietary records (18, 19, 21, 22, 35, 64, 69, 75–77). A total of 21 studies adjusted for physical activities (18, 20–22, 28, 32, 35, 40, 56, 57, 59, 60, 62, 65, 67, 68, 71, 72, 75), and others did not adjust for physical activities, and only one study did not adjust for age (69).

**Table 1:** Main characteristics of prospective studies examined the association of dietary fiber intake with all-cause, cardiovascular, cancer mortality.

**References**	**Location**	**Follow-up (year)**	**Proportion of women**	**Age**	**Sample size**	**Outcome and cases**	**Exposure type**	**Exposure measurement**	**Adjustments**
You et al. (76)	Malaysia	5	52.0%	>60	2,322	All-cause mortality 336	Dietary fiber	24 h recall	Age, gender, marital status and years of education
Zhang et al. (75)	China	11	52.8%	47.35 (mean)	8,307	All-cause mortality468	Dietary fiber	24 h recall	Age, sex, BMI, education, regions, physical activity, smoking status, alcohol drinking, total energy intake, total carbohydrate intake, protein intake, fatty intake, systolic blood pressure, diastolic blood pressure, Na intake, legume fiber, fruit fiber, and vegetable fiber.
Xu et al. (77)	US	17.1	45.80%	62.1 (mean)	86,642	All-cause mortality 17,536; CVD mortality 4,842; Cancer mortality 5,760	Dietary fiber; Insoluble fiber; Soluble fiber	Dietary history method	Age (continuous), sex (male vs. female), race (non-Hispanic White vs. Other), body mass index (BMI, < 25.0 kg/m2 vs. ≥ 25.0 kg/m2), education (≤ high school vs. ≥ some college), smoking status (never vs. former ≤ 15 years since quit vs. former > 15 years since quit vs. former year since quit unknown vs. current smoker ≤ 1 pack per day vs. current smoker > 1 pack per day vs. current smoker intensity unknown), marital status (married vs. not married), alcohol drinking status (never vs. former vs. current), and total energy intake (continuous)
Kwon et al. (32)	Korea	10.1 (median)	61.6%	>40	3,892	All-cause mortality 602; CVD mortality 149	Dietary fiber	FFQ	Age, sex, BMI, smoking, alcohol intake, exercise, total calorie intake, hypertension, diabetes, dyslipidemia, and baseline eGFR
Ha et al. (18)	US	9.3 (median)	51.3%	≥30	20,602	All-cause mortality 3,539; CVD mortality 798; Cancer mortality 714	Dietary fiber	1-d 24-h dietary recall	Age, sex, and race/ethnicity education, smoking, BMI, physical activity, dietary supplement use, and history of cardiovascular disease, diabetes, and hypertension, Adequate Intake;
Ho et al. (19)	UK	10.6 (mean)	55.9%	37–73	195,658	All-cause mortality 4,780	Dietary fiber	24 h recall	Total energy intake and office-based risk factors: age, sex, diabetes, body mass index categories, systolic blood pressure, and smoking. Protein, saturated fatty acids, polyunsaturated fatty acid, monounsaturated fatty acid, starch, sugar
Katagiri et al. (20)	Japan	16.8 (mean)	54.0%	45–74	5,4445	Men: All-cause mortality 11,773; Women: All-cause mortality 7,627; Men: Cancer mortality 11,773; Women: Cancer mortality7627; Men: CVD mortality 11,773; Women: CVD mortality 7,627	Dietary fiber; Soluble fiber; Insoluble fiber; Cereal fiber; Vegetable fiber; Fruit fiber	FFQ	Age, area, BMI, smoking status, alcohol intake, sports or physical exercise during leisure time, hypertension with medication, self-reported diabetes with and without medication, health check-up, amount of green tea intake, coffee intake, salt intake
Miyazawa et al. (29)	Japan	24	56.12%	30–79	8,925	Men: CVD mortality 419; Women: CVD mortality 404; Men: stroke mortality 205; Women: stroke mortality 180	Dietary fiber	Modified Standards Tables for Food Composition in Japan (Third edition)	Age, smoking status, drinking status, BMI, medication of hypertension, past history of diabetes mellitus, sodium, saturated fatty acids, long-chain n-3 polyunsaturated fatty acids, available carbohydrate
Partula et al. (22)	France	5 (median)	78.7%	>18	107,377	All-cause mortality 635	Dietary fiber	Web-based 24-h dietary records	Age, sex, educational level, BMI, physical activity, smoking status, alcohol intake, energy intake, and number of 24-h dietary records. family history of cancer and CVD, and the personal history of cancer, CVD, and T2D.
Dominguez et al. (28)	Spain	10.1 (mean)	61.0%	NR	19,703	All-cause mortality 323	Dietary fiber; Vegetable fiber; Fruit fiber; Legume fiber; Cereal fiber; Soluble fiber; Insoluble fiber	136-item FFQ	Age, sex, marital status, body mass index, smoking, alcohol, physical activity, hours per day spent watching television, baseline hypercholesterolemia, baseline hypertension, history of depression, history of CVD, history of cancer, history of diabetes, following special diets at baseline, snacking between meals, sugar-sweetened beverages consumption, and total energy intake
Chan et al. (35)	US	13.74 (mean)	53.4%	>20	15,740	All-cause mortality 3,164; Cancer mortality 656	Insoluble fiber	24-h dietary recall	Age, sex, race, marital status, education level, energy intake, folate intake, body mass index, alcohol consumption, smoking status and physical activity frequency per week.
Xu et al. (72)	US	14 (mean)	43.9%	61.7 (mean)	367,442	All-cause mortality 38,381; CVD mortality 9,323; Cancer mortality 16,000	Dietary fiber	Self-administered 124-item FFQ	Age, gender, smoking status, smoking dose, and time since quitting smoking, race/ethnicity, education, marital status, self-rated health status, body mass index, physical activity, use of menopausal hormone therapy, and intake of alcohol, red meat, fruits, vegetables, and total energy.
Gopinath et al. (61)	Australia	10 (total)	56.7%	>49	1,609	All-cause mortality 610	Dietary fiber	FFQ	Age, sex, marital status, living status, smoking, and weight status
Huang et al. (62)	US	14 (mean)	43.9%	50–71	36,7442	All-cause mortality 46,067; CVD mortality 11,283; Cancer mortality 19,043	Cereal fiber	A self-administered 124-item FFQ	Age, gender, the number of cigarettes smoked per day, time of smoking cessation, race or ethnicity group, alcohol intake, education level, marital status, health status, obesity, physical activity, consumption of red meat, total fruit and total vegetables, total energy intake, and hormone usage.
Xu et al. (21)	US	10 (median)	0%	70–71	1,110	Men: All-cause mortality 300; Men: CVD mortality 138; Men: Cancer mortality 111	Dietary fiber	7-day dietary record	Protein intake (energy adjusted), age, BMI, smoking, physical activity, education, CVD, diabetes, hyperlipidemia, hypertension, eGFR, UAER, and CRP.
Buil-Cosiales et al. (57)	Spain	5.9	43.0%	55–75	7,216	All-cause mortality 425; CVD mortality 103; Cancer mortality 169	Dietary fiber	A 137-item validated FFQ	Age, sex, smoking status, diabetes, BMI, baseline systolic and diastolic arterial blood pressures, and intervention group and stratified by recruitment center, use of statins, alcohol intake, educational level, physical activity, and total energy intake.
Threapleton et al. (70)	UK	14.3 (median)	100%	50.4 (mean)	31036	Women: CHD mortality 113; Women: stroke mortality 117; Women: CVD mortality 230	Soluble fiber; Insoluble fiber; Cereal fiber; Fruit fiber; Vegetable fiber	Self-administered FFQ	Age, BMI, calories from carbohydrate, fat and protein, ethanol intake, METS, smoking status, socio-economic status.
Crowe et al. (40)	Eight European countries	11.5 (mean)	62.4%	53.8 (mean)	306,331	IHD deaths 2,381	Dietary fiber; Cereal fiber; Fruit fiber; Vegetable fiber	Quantitative FFQ; diet history questionnaires; semi-quantitative FFQ	Stratified by sex, centre and smoking and adjusted for age, alcohol intake, BMI, physical activity, marital status, highest education level, current employment, hypertension, hyperlipidaemia, angina pectoris, diabetes mellitus, polyunsaturated to saturated fat ratio and total energy intake.
Chuang et al. (59)	Multi-national	12.7 (mean)	71.2%	50.8 (mean)	452,717	All-cause mortality 23,582; Men: All-cause mortality 10,366; Women: All-cause mortality 13,216; Men: Cancer mortality 4,039; Women: Cancer mortality 5,575	Cereal fiber; Fruit fiber; Vegetable fiber; Dietary fiber	Extensive self-administered quantitative dietary questionnaires; semiquantitative FFQ; diet-history method; 7-d menu book	Education, smoking, alcohol consumption, BMI, physical activity, and total energy intake.
Nilsson et al. (73)	Sweden	1–19	52.0%	49 (median)	21,596	Men: All-cause mortality:1460; Women: All- cause mortality:923	Dietary fiber	FFQ	BMI, sedentary lifestyle, education, current smoking, intake of alcohol and total energy, Red meat, Fatty fish, Fat, Berries, Boiled coffee, Blood dishes, Vegetables, Bread
Akbaraly et al. (74)	UK	18	30.30%	39–63	7,319	All-cause mortality: 534; Cancer mortality: 259; CVD mortality: 141	Dietary fiber	Semiquantitative FFQ	Sex, age, ethnicity, occupational grade, marital status, smoking status, total energy intake, physical activity, BMI categories, prevalent CVD, type 2 diabetes, hypertension, dyslipidemia, metabolic syndrome, and inflammatory markers
Baer et al. (56)	US	18	100%	30–55	50,112	Women: CVD mortality: 1,026; Women: All-cause mortality: 4,893; Women: Cancer mortality: 1,430	Cereal fiber	Semiquantitative FFQ	Age, Body mass index at age 18 years, Smoking status, Physical activity, Alcohol intake, Nut consumption, Polyunsaturated fat, Glycemic load, Dietary cholesterol, Systolic blood pressure, Personal history of diabetes, Parental MI before age 60 years, Time since menopause
Buyken et al. (58)	Australia	13 (total)	54.5%	≥49	2,735	Women: CVD mortality 109; Men: CVD mortality 151	Dietary fiber; Vegetable fiber; Fruit fiber; Cereal fiber	145-item FFQ	Age, energy, dietary glycemic index residuals, alcohol consumption 20 g/d compared with 20 g/d, current smoking, and presence of diabetes at baseline;
Eshak et al. (60)	Japan	14.3	60.6%	40–79	58,730	Men: CVD mortality 1,063; Women: CVD mortality 1,017; Men: CHD mortality 1,063; Women: CHD mortality 1,017; Men: stroke mortality 1,063; Women: stroke mortality 1,017	Dietary fiber; Insoluble fiber; Soluble fiber; Cereal fiber; Fruit fiber; Vegetable fiber	Self-administered FFQ	Age, BMI, history of hypertension, history of diabetes, alcohol consumption, smoking, education level, hours of exercise, hours of walking, perceived mental stress, sleep fish, SFA, n-3) fatty acids, sodium, folate, and vitamin E.
Kaushik et al. (63)	Australia	13	43.3%	>49	2,897	Stroke mortality 95; CHD mortality NR	Cereal fiber	FFQ.	Age, gender, systolic blood pressure, diastolic blood pressure, antihypertensive medication use, body mass index, smoking status, educational qualifications, fair or poor self-rated health, history of myocardial infarction and stroke, and presence of diabetes, energy
Streppel et al. (69)	Dutch	40	0%	49 (mean)	1,373	Men: CHD mortality 348; Men: All-cause mortality 1,130	Dietary fiber; Vegetable fiber; Fruit fiber; Cereal fiber	Cross-check dietary history method	Total energy, saturated fat, trans unsaturated fatty acid, and cis polyunsaturated fat acid intakes; alcohol intake, wine use, fish intake, prescribed diet, the number of cigarettes smoked, the duration of cigarette smoking, cigar or pipe smoking, BMI, and socioeconomic status.
Lubin et al. (66)	Israeli	18	52.0%	55.2 (mean)	623	All-cause mortality 151	Dietary fiber	FFQ	Mean daily energy intake, Ethnic origin, Sex, Age, 5-y increment, Smoking status, Systolic blood pressure, Physical activity, BMI, Fatty acids, Energy intake from fat, Cholesterol
Mozaffarian et al. (67)	US	8.6 (mean)	38.8%	>65	3,588	IHD deaths159	Cereal fiber; Fruit fiber	99-item FFQ	Age, sex, education, diabetes, ever smoking, pack-years of smoking, daily physical activity, exercise intensity, alcohol intake, and cereal, fruit, and vegetable fiber intake
Liu et al. (65)	US	6 (mean)	100%	≥45	Women: 38,480	Women: CVD mortality 570	Dietary fiber; Cereal fiber; Vegetable fiber; Fruit fiber; Soluble fiber; Insoluble fiber	A validated 131-item SFFQ	Age, randomized treatment assignment, smoking status, exercise, alcohol intake, use of postmenopausal hormone, body mass index, use of multivitamin supplements, history of hypertension, history of high cholesterol, history of diabetes, parental history of MI before age 60, dietary folate, total fat, protein, and total energy intake.
Todd et al. (71)	UK	3 (total)	100%	40–59	3,833	Women: All-cause mortality 108	Dietary fiber	Semiquantitative FFQ	Age, serum total cholesterol, systolic blood pressure, carbon monoxide, energy, previous medical diagnosis of diabetes, body mass index, the Bortner personality score, triglycerides, high density llpoproteln cholesterol, fibrinogen, a self-reported measure activity in leisure, and alcohol consumption
Pietinen et al. (68)	Finland	6.1	0%	50–60	21,930	Men: CHD mortality 1,399;	Dietary fiber; Soluble fiber; Insoluble fiber; Cereal fiber; Vegetable fiber; Fruit fiber	A self-administered modified dietary history method.	Age, treatment group, smoking; body mass index; blood pressure; intakes of energy, alcohol, and saturated fatty acids, education, and physical activity, intakes of beta-carotene, vitamin C, and vitamin E
Khaw et al. (64)	US	12 (mean)	58.6%	50–79	859	Men: IHD deaths 42; Women: IHD deaths 23; IHD deaths 356; Men: IHD deaths 42; Women: IHD deaths 23	Dietary fiber	A 24-hour dietary recall	Age, systolic blood pressure, plasma cholesterol, fasting blood glucose, obesity, cigarette smoking habit

CVD, cardiovascular disease; FFQ, food-frequency questionnaire; US, United States; eGFR, estimated glomerular filtrationrate; BMI, Body mass index; NR, Not reported; UAER, Urinary Albumin Excretion Rate; CRP, C-reactive protein; UK, United Kingdom; CHD, Coronary Heart Disease; IHD, ischemic heart disease; T2D, Type 2 Diabetes Mellitus; SFA, Saturated Fatty Acid; METS, Metabolic Equivalants; MI, myocardial infarction; SFFQ, Simplified Food Frequency Questionnaire.

In all, 22 prospective cohort studies were summarized for meta-analysis to evaluate the possible relationships between dietary fiber consumption and mortality risk, totaling 171,751 deaths (164,183 for all-cause, 95,879 for CVD, and 107,114 for cancer mortality) among 2,567,890 participants. A total of 21 articles reported RRs of all-cause mortality (18–22, 28, 32, 35, 57, 59, 61, 62, 66, 69, 71–77), 11 reported RRs of cancer mortality (18–22, 28, 32, 35, 57, 59, 61, 62, 66, 69, 71–74, 77), 5 reported RRs of mortality from coronary heart disease (60, 63, 68–70), 14 reported RRs of mortality from CVD (18, 20, 21, 29, 32, 56–58, 60, 62, 65, 70, 72, 77), three reported RRs of mortality from ischemic heart disease (40, 64, 67), and four reported RRs of mortality from stroke (29, 60, 63, 70). Assessment of quality of the included studies for the association between dietary fiber and mortality is shown in Supplementary Table 2. By applying the NOS, the mean quality assessment score of included studies was 7.39 (range 5–8), with 28 studies assessed as high quality (more than 7 points) (18–22, 28, 29, 32, 35, 40, 56–60, 62–68, 70–73, 75, 77) and the other four (61, 69, 74, 76) as good quality.

The results of the highest vs. lowest meta-analyses on the associations between intake of dietary fiber and all-cause, CVD, and cancer mortality are shown in Table 1, Supplementary Figures 1–12.

### 3.2. Dose–response meta-analysis

#### 3.2.1. Dietary fiber

**Figure 2:**
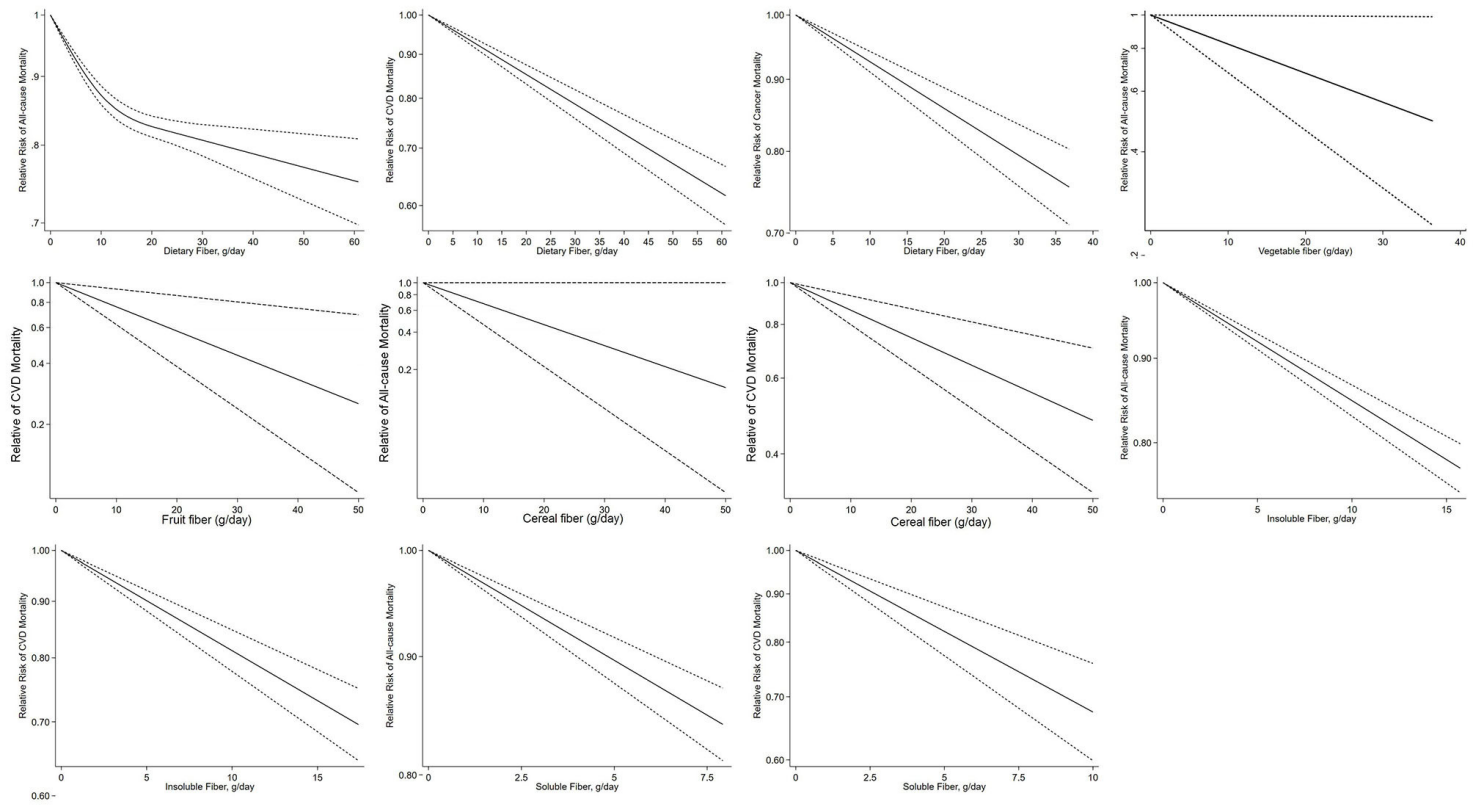
Dose–response association of per 10 g/day increase in total fiber, vegetable, fruit, cereal, soluble and insoluble fiber intake with all-cause, cardiovascular, and cancer mortality by restricted cubic splines.

A total of 14 studies (18–22, 28, 32, 57, 59, 69, 72, 75–77) with a total of 1,367,285 participants and 97,469 deaths were included in the dose–response meta-analysis of dietary fiber intake and all-cause mortality. The summary RR for a 10-g/day increment of dietary fiber intake was 0.90 (95% CI: 0.86–0.93; *I*^2^ = 86.1%, *P*_*heterogeneity*_ < 0.001; Table 2, Supplementary Figure 19). Evidence of heterogeneity between subgroups in stratified analyses was not found (Supplementary Figure 7). A non-linear dose–response association was found between dietary fiber intake and all-cause mortality (*P*_*non*−*linearity*_ = 0.0096, Figure 2). The shape of the non-linear curve was steeper with a dietary fiber intake of < 15 g/day, but the increase was more gradual after 15 g/day.

**Table 2:** Dietary fiber intake and risk of all-cause, cardiovascular, and cancer mortality for the highest versus lowest and dose-response meta-analysis.

		**Highest vs. lowest fiber analysis**				**Dose-response analysis**			
		**No of studies**	**RR (95% CI)**	***I** ^2^ **%***	***P*** **value**	**No of studies**	**RR (95% CI)**	***I** ^2^ **%***	***P*** **value**
Dietary fiber	All-cause mortality	16	0.81(0.77,0.86)	71.90	< 0.001	14	0.90(0.86,0.93)	86.10	< 0.001
	CVD mortality	14	0.78(0.72,0.84)	63.20	0.001	13	0.87(0.84,0.91)	77.80	< 0.001
	Cancer mortality	6	0.82(0.77,0.87)	58.70	0.033	7	0.91(0.88,0.93)	27.90	0.216
Vegetable fiber	All-cause mortality	5	0.96(0.83,1.10)	69.30	0.011	4	0.88(0.73,1.05)	49.60	0.114
	CVD mortality	7	0.87 (0.81,0.94)	0.00%	0.952	7	0.91(0.78,1.06)	0.00	0.498
	Cancer mortality	-	-	-	-	2	0.89(0.79,1.01)	47.30	0.168
Fruit fiber	All-cause mortality	5	0.89(0.81,0.98)	19.00	0.294	4	0.99(0.92,1.07)	27.60	0.246
	CVD mortal ity	11	0.79(0.68,0.92)	71.90	< 0.001	8	0.76(0.52,1.09)	73.30	0.001
	Cancer mortality	-	-	-	-	-	-	-	-
Cereal fiber	All-cause mortality	5	0.88(0.80,0.97)	84.70	< 0.001	5	0.82(0.73,0.93)	56.00	0.059
	CVD mortality	13	0.87(0.81,0.94)	46.40	0.033	9	0.84(0.73,0.97)	47.10	0.057
	Cancer mortality	3	0.86(0.83,0.90)	0.00	0.589	2	0.77(0.56,1.06)	90.20	0.001
Insoluble fiber	All-cause mortality	5	0.85(0.78,0.93)	79.20	0.001	5	0.86(0.81,0.92)	71.30	0.008
	CVD mortality	6	0.74(0.69,0.79)	0.00	0.986	6	0.81(0.78,0.85)	0.00	0.647
	Cancer mortality	3	0.92(0.74,1.14)	82.90	0.003	3	0.93(0.81,1.07)	87.30	< 0.001
Soluble fiber	All-cause mortality	5	0.91(0.85,0.97)	66.80	0.017	5	0.83(0.74,0.92)	60.90	0.037
	CVD mortality	5	0.79(0.72,0.86)	0.00	0.719	5	0.62(0.47,0.84)	63.80	0.026
	Cancer mortality	2	0.97(0.63,1.51)	88.80	0.003	2	0.97(0.55,1.70)	89.00	0.003

CVD, cardiovascular disease; RR, relative risk.

Thirteen studies (18, 20, 21, 29, 32, 40, 57, 58, 60, 64, 65, 72, 77) on the association between dietary fiber intake and CVD mortality were included in the dose–response analysis, which included 945,653 participants and 78,735 deaths. The summary RR for a 10-g/day increment of dietary fiber intake was 0.87 (95% CI: 0.84–0.91; *I*^2^ = 79.2%, *P*_*heterogeneity*_ < 0.001; Table 2, Supplementary Figure 19). Evidence of heterogeneity between subgroups was observed in the analysis stratified by adjustment for comorbidity at baseline (*P* = 0.032) (Supplementary Figure 7). No evidence of a non-linear dose–response association was found between dietary fiber intake and risk of CVD mortality (*P*_*non*−*linearity*_ = 0.247, Figure 2). Dose–response analysis of six studies (18, 20, 21, 57, 59, 72) showed an inverse association between dietary fiber and cancer mortality (summary RR 0.90, 95% CI: 0.87–0.94; *I*^2^ = 35.4%, *P*_*heterogeneity*_ = 0.17; Table 2, Supplementary Figure 19). Evidence of heterogeneity between subgroups was observed in the analysis stratified by adjustment for region (*P* = 0.032) (Supplementary Figure 7). There was no indication of non-linearity between dietary fiber intake and risk of cancer mortality (*P*_*non*−*linearity*_ = 0.995, Figure 2).

Sensitivity analysis showed that the exclusion of any single study from the analysis did not appreciably alter the summary effect sizes (Supplementary Table 9).

#### 3.2.2. Vegetable fiber

Four studies (22, 28, 59, 69) were included in the dose–response meta-analysis of vegetable fiber intake and all-cause mortality. The summary RR for a 10-g/day increment of vegetable fiber intake was 0.88 (95% CI: 0.73–1.05; *I*^2^ = 49.6%, *P*_*heterogeneity*_ = 0.11; Table 2, Supplementary Figure 20). No evidence of heterogeneity between subgroups was observed (Supplementary Figure 8). The non-linearity between dietary fiber intake and risk of cancer mortality approached significance (*P* = 0.07, Figure 2).

No significant association was seen between vegetable fiber intake and CVD mortality based on six studies (40, 58, 65, 68–70). The summary RR for a 10-g/day increment of vegetable fiber was 0.91 (95% CI: 0.78–1.06; *I*^2^ = 0%, *P*_*heterogeneity*_ = 0.50; Table 2, Supplementary Figure 20). No evidence of heterogeneity between subgroups was observed (Supplementary Figure 8). A non-linear dose–response association was found between vegetable fiber intake and risk of cancer mortality (*P*_*non*−*linearity*_ = 0.01, Figure 2). The association between vegetable fiber and CVD mortality has a J-shape, with the lowest estimates at 5 g/day.

In the sensitivity analysis, exclusion of the study by Partula et al. (22) and Streppel et al. (69) resulted in a change in the significant inverse association between vegetable fiber intake and all-cause mortality to a marginally significant inverse association, but the summary estimate of vegetable fiber intake and CVD mortality remained robust (Supplementary Table 10).

#### 3.2.3. Fruit fiber

No significant association was seen between fruit fiber intake and all-cause mortality based on four studies (22, 28, 59, 69). The summary RR for a 10-g/day increment of fruit fiber intake was 0.99 (95% CI: 0.92–1.07; *I*^2^ = 27.6%, *P*_*heterogeneity*_ = 0.25; Table 2, Supplementary Figure 21). No significant association with fruit fiber intake was found in subgroup analyses, and no evidence of heterogeneity between subgroups was observed (Supplementary Figure 9). There was no indication of non-linearity between fruit fiber intake and all-cause mortality (*P*_*non*−*linearity*_ = 0.25, Figure 2).

No significant association was found between fruit fiber intake and CVD mortality based on seven studies studies (40, 58, 60, 65, 68–70). The summary RR for a 10-g/day increment of fruit fiber intake was 0.76 0.52–1.09; *I*^2^ = 73.3%, *P*_*heterogeneity*_ = 0.001; Table 2, Supplementary Figure 21). No evidence of heterogeneity between subgroups was observed (Supplementary Figure 9). There was no evidence of non-linearity between fruit fiber intake and CVD mortality (*P*_*non*−*linearity*_ = 0.13, Supplementary Figure 14).

A sensitivity analysis showed that exclusion of the studies by Eshak et al. (60) or Pietinen et al. (68) resulted in a change from the non-significant association between fruit fiber intake and CVD mortality to a significant inverse association (Supplementary Table 11).

#### 3.2.4. Cereal fiber

In the dose–response analysis of cereal fiber intake and all-cause mortality, based on five studies studies (22, 28, 56, 59, 69), a significant inverse association was found. The summary RR for a 10-g/day increment cereal fiber intake was 0.82 (95% CI: 0.73–0.93; *I*^2^ = 56.0%, *P*_*heterogeneity*_ = 0.06; Table 2, Supplementary Figure 22). No evidence of heterogeneity between subgroups was observed (Supplementary Figure 10). There was no indication of non-linearity between soluble fiber intake and CVD disease mortality (*P* = 0.24, Figure 2).

In the dose–response analysis of cereal fiber intake and CVD mortality, based on nine studies (40, 56, 58, 60, 63, 65, 68–70), a significant inverse association was found. The summary RR for a 10-g/day increment of cereal fiber intake was 0.84 (95% CI: 0.73–0.97; *I*^2^ = 47.1%, *P*_*heterogeneity*_ = 0.06; Table 2, Supplementary Figure 22). No evidence of heterogeneity between subgroups was observed (Supplementary Figure 10). There was no indication of non-linearity between cereal fiber intake and CVD mortality (*P*_*non*−*linearity*_ = 0.45, Figure 2), with nine studies included (40, 56, 58, 60, 63, 65, 68–70).

Two studies (56, 59) reported data on cereal fiber intake and cancer mortality. The summary RR for a 10-g/day increment of cereal fiber intake was 0.77 (95% CI: 0.56–1.06; *I*^2^ = 90.2%, *P*_*heterogeneity*_ = 0.001; Table 2, Supplementary Figure 22).

The sensitivity analysis showed that the summary estimate is robust (Supplementary Table 12).

#### 3.2.5. Insoluble fiber

Five studies (20, 22, 28, 35, 77) assessed the dose–response meta-analysis of insoluble fiber intake and all-cause mortality. The summary RR for a 10-g/day increment of insoluble fiber intake was 0.86 (95% CI: 0.81–0.92, *I*^2^ = 71.3%, *P*_*heterogeneity*_ = 0.008; Table 2, Supplementary Figure 23). Evidence of heterogeneity between subgroups was observed in the analysis stratified by the number of cases included in the study (0.034) and whether adjusted for region (*P* = 0.040) (Supplementary Figure 11). There was no indication of non-linearity between insoluble fiber intake and all-cause mortality (*P*_*non*−*linearity*_ = 0.909, Figure 2), with five studies included (20, 22, 28, 35).

Six studies (20, 60, 65, 68, 70, 77) on the association between insoluble fiber intake and CVD mortality were included in the dose–response analysis. The summary RR for a 10-g/day increment of insoluble fiber intake was 0.81 (95% CI: 0.78–0.85; *I*^2^ = 0.00%, *P*_*heterogeneity*_ = 0.65; Table 2, Supplementary Figure 23). No evidence of heterogeneity between subgroups was observed (Supplementary Figure 11). There was no evidence of non-linear dose–response association between insoluble fiber intake and CVD mortality (*P*_*non*−*linearity*_ = 0.52, Figure 2), with six studies included (20, 60, 65, 68, 70).

The dose–response analysis of three studies (20, 35, 77) showed no significant association between insoluble fiber and cancer mortality (summary RR: 0.93, 95% CI: 0.81–1.07), with no significant heterogeneity among the studies (*I*^2^ = 87.3%, *P*_*heterogeneity*_ < 0.001; Table 2, Supplementary Figure 23). There was no evidence of non-linear dose–response association between insoluble fiber intake and CVD mortality (*P*_*non*−*linearity*_ = 0.699, Figure 2), with two studies included (20, 35).

In the sensitivity analysis, the summary estimate is robust for all-cause and CVD mortality. Exclusion of the study by Katagiri et al. (20) resulted in a change from the non-significant association between insoluble fiber intake and cancer mortality to a significant inverse association (Supplementary Table 13).

#### 3.2.6. Soluble fiber

Five prospective studies (20, 22, 28, 35, 77) were included in the dose–response meta-analysis of soluble fiber intake and all-cause mortality. The summary RR for a 10-g/day increment of soluble fiber intake was 0.83 (95% CI: 0.74–0.92; *I*^2^ = 60.9%, *P*_*heterogeneity*_ = 0.037; Table 2, Supplementary Figure 24). Evidence of heterogeneity between subgroups was observed in the analysis stratified by dietary fiber measurement (*P* = 0.032) (Supplementary Figure 12). There was no indication of non-linearity between soluble fiber intake and all-cause mortality (*P*_*non*−*linearity*_ = 0.785, Figure 2), with five studies included (20, 22, 28, 35).

Five studies (60, 65, 68, 70, 77) provided RRs of soluble fiber intake and CVD mortality. The summary RR for a 10-g/day increment of soluble fiber intake was 0.62 (95% CI: 0.47–0.84; *I*^2^ = 63.8%, *P*_*heterogeneity*_ = 0.026; Table 2, Supplementary Figure 24). Evidence of heterogeneity between subgroups in stratified analyses was not observed (Supplementary Figure 12). There was no indication of non-linearity between soluble fiber intake and CVD mortality (*P*_*non*−*linearity*_ = 0.587, Figure 2).

In the sensitivity analysis, the summary estimate is robust, except that exclusion of the study by Katagiri et al. and Xu et al. (20, 77) lead to a non-significant association between soluble fiber intake and all-cause mortality (Supplemental Table 14).

### 3.3. Publication bias

In the highest vs. lowest meta-analysis, Egger's linear regression test and visual inspection of the funnel plots (Supplementary Figure 25) indicated possible publication bias for the association between dietary fiber intake and CVD mortality (*P* = 0.001), and vegetable fiber intake and all-cause mortality (*P* = 0.038), but no evidence of publication bias for other outcomes. In the dose–response analyses, Egger's linear regression test and visual inspection of the funnel plots indicated possible publication bias for the association between dietary fiber intake and cancer mortality (*P* = 0.043) (Supplementary Figures 31, 36). No evidence of significant publication bias was found in other analyses (Supplementary Figures 26–30, 32–35). Application of the trim and fill method did not result in a change in the average effect size, further suggesting that the results were not affected by publication bias.

## 4. Discussion

The present systematic review and meta-analysis investigated the association between dietary fiber intake and different sources and types of fiber intake and all-cause, CVD, and cancer mortality by applying highest vs. lowest, linear, and non-linear dose–response analyses. We found that dietary fiber intake was inversely associated with all-cause, CVD, and cancer mortality. The inverse association was also found for cereal fiber intake. All categories of fibers were inversely associated with CVD mortality. The inverse association of cancer mortality was only detected for cereal fiber and dietary fiber. Significant associations were also found for other fiber intake and all-cause mortality, except for fruit and vegetable fiber intake. Besides, a non-linear relationship was found for all-cause mortality.

A large number of longitudinal cohort studies have reported the health benefits of dietary fiber intake (78–80). Several systematic reviews and meta-analyses suggested that high dietary fiber intake was associated with a reduced risk of all-cause, CVD, and cancer mortality (42, 46), which was consistent with the findings from our systematic review and meta-analysis. The subgroup analysis also showed the stability of the findings, which was different from previous meta-analyses meta-analyses (24). This may account for the fact that our study has additionally included more than 14 related studies studies (18–22, 28, 29, 32, 35, 61, 62, 75–77) published in recent years, with than 2,614,294 participants included, compared to the previous meta-analysis. This study found a non-linear relationship between dietary fiber and all-cause mortality, showing that the protective effect of dietary fiber is relatively constant when the daily intake is >15 g. A meta-analysis including five papers concluded that risk reduction associated with all-cause mortality was greatest when the daily intake of dietary fiber was between 25 and 29 g, while the dose–response data suggested that amounts >30 g/day confer additional benefits (26). The inconsistent findings might be because of the relatively large number of studies included: publications since 2016 were not included in their dose–response analyses of dietary fiber intake and all-cause mortality (26), and ~14 more articles updated to 2023 were included in our dose–response meta-analysis. The non-linear relationship of dietary fiber was not found among all-cause and cancer mortality because the effect of dietary fiber on different health outcomes may have different mechanisms.

Dietary fibers from different food sources have a distinctive mix of different types of compounds and may have a different effect on all-cause and CVD mortality (81, 82). The present systematic review and meta-analysis found the inverse association between vegetable and fruit fiber intake and CVD mortality as well as the significantly inverse association between cereal fiber intake and all-cause, CVD, and cancer mortality, but no association of vegetable fiber with cancer or all-cause mortality. A meta-analysis also found that cereal fiber intake was protectively associated with all-cause, CVD, and cancer mortality, although it included general participants and people with diseases (41). Our study also showed that cereal fiber but not fruit fiber or vegetable fiber was significantly associated with lower total mortality in the dose–response analysis, which was in line with an earlier meta-analysis (45). The recommended level of dietary fiber intake is 25 g for adult women and 38 g for adult men, and the public should consume adequate amounts of dietary fiber from a variety of plant foods (83). Plant foods contain more than just dietary fiber, so any protective properties of plant-based diets may be linked to other dietary components, such as vitamins, minerals, or phytochemicals, and not just isolated dietary fiber (84). The unstable findings in the subgroup analysis suggest that more studies are further needed on the association between fruit fiber and CVD mortality.

Soluble fiber is found in oat bran, barley, beans, lentils, peas, and some fruits and vegetables, while insoluble fiber is rich in foods such as wheat bran, whole grains, nuts, and seeds (77). Although mounting evidence has suggested the protective role of dietary fiber against various chronic diseases (13, 22), the health effect may depend on the dietary fiber type (85, 86), and the findings on soluble and insoluble fiber intake and mortality are contradictory (20, 22). Our study found the inverse association between both soluble and insoluble fiber intake and all-cause and CVD mortality. The finding on CVD mortality was in line with one previous systematic review and meta-analysis (87), and our study included eight additional studies (18, 20, 21, 29, 32, 57, 72, 77) after 2012 and found a linear relationship. To the best of our knowledge, this is the first study to explore soluble and insoluble fiber intake and all-cause and cancer mortality. No significant association was found between insoluble or soluble fiber intake and cancer mortality in the present study, which may be explained by the limited number of studies included. Insoluble fiber is characterized by a fecal-bulking ability, which may reduce the risk of cancer mortality (77); however, evidence regarding soluble or insoluble fiber on cancer mortality remains limited and inconsistent, and only three studies (20, 35, 77) conducted in Japan and the United States were included in our systematic review and meta-analysis. Further prospective studies on soluble and insoluble fiber intake and cancer mortality are therefore needed.

The mechanism underlying the inverse relationship between dietary fiber and mortality is unclear, but there are several plausible explanations. The protective effect of dietary fiber on cholesterol (88, 89), blood pressure (90), insulin sensitivity (85), and blood glucose (91) as well as the anti-inflammatory effects (92) may partly explain the protection from mortality. A study demonstrated that the inclusion of a practical dose of dietary fiber (11.6 g) in a bakery product significantly reduced postprandial glucose and insulin responses in healthy adults (93). Insulin is known to promote the action of the hepatic enzyme 3-hydroxy-3-methylglutaryl-coenzyme A (HMG-CoA) reductase (94). Inhibition of HMG-CoA reductase may result in the prevention of excess cholesterol being synthesized and released into circulation by the liver and may thereby reduce the risk of CVD (95). Moreover, alteration of intestinal microbiota composition and function may be an important reason for the potential benefits of dietary fiber (96). Experimental studies also suggested that the reduction of soluble fiber may influence the synthesis of microbial metabolites that are important for regulating metabolic, immune, behavioral, and neurobiological outcomes (97).

This review has some strengths. First, the present study was a comprehensive systematic review and meta-analysis of prospective cohort studies to investigate the association between dietary fiber intake and mortality, using high vs. low analysis and dose–response analysis. Second, the different types and food sources of dietary fiber were also considered, which can provide valuable insight into the mechanisms and evidence for strategies to derive the greatest benefit from balanced consumption of dietary fiber. Furthermore, a large number of participants and deaths have been included and allowed us to quantitatively assess the association between dietary fiber intake and risk of mortality.

In terms of study limitations, first of all, most studies did not consider other nutrients as confounding factors, such as protein, carbohydrate, or fiber from other food sources, which may affect the magnitude of the association between dietary fiber intake and mortality. Besides, comorbidity at baseline was not controlled in a few studies, which could affect the association between dietary fiber and mortality. Second, different dietary fiber assessment tools were used, which might lead to variation in the study results. Third, only three studies (20, 35, 77) reported risk estimates on soluble or insoluble fiber intake and cancer mortality, which limit us to conduct the subgroup and sensitivity analyses and suggest the necessity of further studies. Fourth, different diet assessment tools were used (FFQ, 24-h dietary recall, semiquantitative FFQ), and therefore measurement error was unavoidable. Fifth, sensitivity analyses demonstrated a profound lack of robustness among summary estimates for vegetable fiber and fruit fiber intake on mortality in the dose–response meta-analysis. Sixth, high heterogeneity exists in our meta-analysis of fruit fiber–CVD mortality and dietary fiber–all-cause mortality associations, although sensitive and subgroup analyses were conducted to show stable findings. The meta-regression analysis was also conducted, and we found that the heterogeneity may come from different levels of study quality for studies included in the meta-analysis of dietary fiber and all-cause mortality and different durations of follow-up for the studies on the association of fruit fiber and CVD mortality.

In conclusion, the present systematic review and meta-analysis found that higher dietary fiber intake was associated with a lower risk of all-cause, CVD, and cancer mortality. For different food sources of dietary fibers, fruit, vegetable, and cereal fiber intake were related to reduced risk of mortality, but there was no association of vegetable or fruit fiber with cancer mortality, showing a significant non-linear relationship between dietary fiber intake and all-cause mortality and a linear relation for other fibers. Our study incorporates different types and food sources of dietary fibers, which provide valuable insight into the mechanisms and may provide evidence for strategies to derive the greatest benefit from a balanced consumption of dietary fiber. The association between insoluble or soluble fiber intake and mortality and the difference between sources of dietary fiber and cancer mortality warrants further investigation.

## Data availability statement

The original contributions presented in the study are included in the article/Supplementary material, further inquiries can be directed to the corresponding author.

## Author contributions

FY and PQ conceived, designed, performed the study, and drafted the manuscript. FY, JM, CH, XZ, YC, RL, and PQ extracted, analyzed, or interpreted the data. FY, JM, YC, RL, CH, XZ, FH, and PQ revised the manuscript. All authors approved the final manuscript.
