# Supplementary material for: Dietary intake of total vegetable, fruit, cereal, soluble and insoluble fiber and risk of all-cause, cardiovascular, and cancer mortality: systematic review and dose–response meta-analysis of prospective cohort studies
Source: Front Nutr. 2023 Oct 3;10:1153165. doi: 10.3389/fnut.2023.1153165 (PMC10579821; doi:10.3389/fnut.2023.1153165)
Supplement: Supplementary file 1 [file Data_Sheet_1.docx]

Supplementary Material

Dietary intake of total, vegetable, fruit, cereal, soluble and insoluble fiber and risk of all cause, cardiovascular, and cancer mortality: systematic review and dose-response meta-analysis of prospective cohort studies

**Feifei Yao^1^, Jianping Ma^2^, Yong Cui^3^, Cuihong Huang^4^, Ruiqi Lu^5^,** **Fulan Hu^6^,Xiaoming Zhu^7^,Pei Qin^1*^**

*** Correspondence:**

Pei Qin Email：[qinpei225@163.com](mailto:qinpei225@163.com)

1. **Highest versus Lowest Dietary Fiber Intake**

***Dietary Fiber***

Of the 16 studies (1-16) on intake of dietary fiber and all-cause mortality, 12 studies reported an inverse association(1-3, 5-9, 11, 12, 15, 16) and other 4 reported no significant association(4, 10, 11, 14). For the association between intake of dietary fiber and CVD mortality, ten studies showed an inverse association(1, 7, 14, 16-22),and the others indicated no significant association(5, 8, 18-20, 23, 24). For cancer mortality, five studies showed a protective association with intake of dietary fiber (2, 5, 7, 12, 16).

Sixteen studies (1-16) were assessed to estimate risk between intake of dietary fiber and all-cause mortality, including 3,177,061 participants and 181,948 deaths. The summary RR for all-cause mortality comparing the highest and lowest intakes of dietary fiber was 0.81 (95% CI: 0.77,0.86; *I^2^*=71.9%, *P_heterogeneity_*<0.001) (Table 2, Supplemental Figure 13). The inverse associations persisted in additional subgroup analyses. Evidence of heterogeneity between subgroups in stratified analyses was observed for studies stratified by region, whether adjust for carbohydrate (Supplemental Figure 1).

The association between consumption of dietary fiber and CVD mortality was examined in 14 studies (1, 5, 7, 8, 16-21, 23, 24),including a total of 966,473participants and 35,019 deaths. Significant inverse association was found (summary RR comparing highest and lowest intakes was 0.78, 95% CI: 0.72,0.84), with significant heterogeneity among the studies (*I^2^*=63.2%, *P_heterogeneity_* =0.001; Table 2, Supplemental Figure 13). Evidence of heterogeneity between subgroups in stratified analyses was observed for studies stratified by region and whether adjusted for PA(Supplemental Figure 13).

The association between dietary fiber intake and cancer mortality was examined in 6 studies (1, 2, 5, 7, 12, 16),including a total of 977,373 participants and 11,335 deaths. An inverse association was also found (summary RR comparing highest and lowest intakes was 0.82, 95% CI: 0.77,0.87; *I*^2^=58.7%, *P_heterogeneity_* =0.03; Table 2, Supplemental Figure 13). Consistent associations were observed in the subgroup analysis and no evidence of heterogeneity between subgroups in stratified analyses was observed (*P* >0.05) (Supplemental Figure 1).

Sensitivity analysis showed that exclusion of any single study from the analysis did not appreciably alter the summary effect sizes and the summary RRs ranged from 0.81 (95% CI: 0.72 to 0.92) to 0.84 (95% CI: 0.78 to 0.90) (Supplemental Table 3).

***Vegetable Fiber***

Of the 5 studies (3, 4, 7, 10, 14) on intake of vegetable fiber and all-cause mortality, 2 studies(3, 7) reported an inverse association and other 3 studies(4, 10, 14) reported no significant association. For the association between intake of vegetable fiber and cardiovascular mortality, one study(7) showed an inverse association, and the others indicated no significant association(17, 18, 21, 23-25).

Five studies (3, 4, 7, 10, 14) were assessed to estimate risk between intake of vegetable fiber and all-cause mortality, including 232,142 participants and 21,759 deaths. The summary RR for all-cause mortality comparing the highest and lowest intakes of vegetable fiber was 0.96 (95% CI: 0.83,1.10; *I^2^*=69.3%, *P_heterogeneity_* =0.011) (Table 2, Supplemental Figure 14). No evidence of heterogeneity between subgroups was observed in stratified analyses (Supplemental Figure 14).

The association between consumption of vegetable fiber and CVD mortality was examined in 7 studies(7, 17, 18, 21, 23-25), including a total of 552,166 participants and 26,320 deaths. Significant inverse association was found (summary RR comparing highest and lowest intakes was 0.87, 95% CI: 0.81 to 0.94), with no significant heterogeneity among the studies (*I*^2^=0.0%, *P_heterogeneity_* =0.95; Table 2, Supplemental Figure 14). No evidence of heterogeneity between subgroups was observed in stratified analyses (*P* >0.05) (Supplemental Figure 14).

In the sensitivity analysis, no single study substantially changed the statistical significance or direction of the combined RR, except that exclusion of the study by Katagiri et al(7) and Dominguez(3) showed a non-significant association for all-cause (RR: 0.99, 95% CI: 0.78 to 1.28; RR: 0.99, 95% CI: 0.77 to 1.29) , and exclusion of the study by Katagiri et al(7) showed a non-significant association for CVD mortality (RR: 0.90, 95% CI: 0.81to 1.01) (Supplemental Table 4).

***Fruit Fiber***

Of the 5 studies(3, 4, 7, 10, 14) on intake of fruit fiber and all-cause mortality, 1 study(7) reported an inverse association and other 4 studies(3, 4, 10, 14) reported no significant association. For the association between intake of fruit fiber and cardiovascular mortality, 4 studies(7, 17, 18, 23) showed an inverse association and the others indicated no significant association.

Five studies (3, 4, 7, 10, 14) with a total of 28,793 participants and 21,436 deaths were included to test the association between intake of fruit fiber and risk of all-cause mortality. The summary RR for all-cause mortality comparing the highest and lowest intakes of dietary fiber was 0.89 (95% CI: 0.81, 0.98; *I^2^*=19.0%, *P_heterogeneity_* =0.29; Table 2, Supplemental Figure 15). Evidence of heterogeneity between subgroups in stratified analyses was observed by region and whether adjusted for number of cases included in study (Supplemental Figure 3).

The association between consumption of fruit fiber and CVD mortality was examined in 8 studies(7, 17, 18, 21, 23-26) including a total of 555,754 participants and 26,479 deaths. Significant inverse association was found (summary RR comparing highest and lowest intakes was 0.81, 95% CI: 0.69 to 0.97; *I*^2^=76.6%, *P_heterogeneity_* <0.001; Table 2, Supplemental Figure 15). No evidence of heterogeneity between subgroups was observed in stratified analyses (*P* >0.05) (Supplemental Figure 3).

In the sensitivity analysis, no single study substantially changed the statistical significance or direction of the combined RR , except that exclusion of the study by Katagiri et al(7) showed a non-significant association (RR: 0.92, 95% CI: 0.81 to 1.05) for all-cause mortality, and studies by Threapleton et al, Eshak et al , Mozaffarian et al, and Xu et al(14, 18, 25, 26) (Supplemental Table 5).

### *Cereal Fiber*

Of the 4 studies(3, 7, 10, 27) on intake of cereal fiber and all-cause mortality, 2 studies(7, 27) reported an inverse association and other 2(3, 10) reported no significant association. For the association between intake of cereal fiber and cardiovascular mortality, four studies(17, 18, 21, 27, 28) showed an inverse association and the others indicated no significant association.

Four studies(3, 7, 10, 27) were assessed to estimate risk between cereal fiber intake and risk of all-cause mortality, including 569,965 participants and 66,425 deaths. The summary RR for all-cause mortality comparing the highest and lowest intakes of cereal fiber was 0.86 (95% CI: 0.77 to 0.97; *I^2^*=87.1%, *P_heterogeneity_* <0.001; Table 2, Supplemental Figure 16). Evidence of heterogeneity between subgroups in stratified analyses was observed for studies stratified by region (*P*=0.04) and whether to adjust for comorbidity at baseline (*P*=0.04). Subgroup analyses showed a significant association for studies that conducted in Asia and North America. The inverse association was similar among studies stratified by score and whether controlled for comorbidity at baseline. (Supplemental Figure 4).

The association between consumption of cereal fiber and CVD mortality was examined in 10 studies(7, 17, 18, 21, 23-28), including a total of 926,093 participants and 72,641 deaths. Significant inverse association was found (summary RR comparing highest and lowest intakes was 0.86, 95% CI: 0.79 to 0.94; *I^2^*=58.7%, *P_heterogeneity_* =0.01; Table 2, Supplemental Figure 16). Evidence of heterogeneity between subgroups in stratified analyses was observed for studies stratified by whether to control for comorbidity at baseline (*P*=0.019). Subgroup analyses showed a significant association for studies that did not control for protein intake, carbohydrate intake or comorbidity at baseline. (Supplemental Figure 4).

The association between cereal fiber intake and cancer mortality was examined in 2 studies(7, 27), with a total of 460,366 participants and 65,467 deaths. The summary RR for cancer mortality comparing the highest and lowest intakes of cereal fiber was 0.86 (95% CI: 0.83 to 0.90; *I*^2^=0%, *P_heterogeneity_* =0.33; Table 2, Supplemental Figure 16).

### Sensitivity analysis showed that exclusion of any single study from the analysis did not appreciably alter the summary effect sizes and the summary RRs ranged from 0.85 (95% CI: 0.79 to 0.92) to 0.93 (95% CI: 0.89 to 0.97) (Supplemental Table 6).

### *Insoluble Fiber*

Of the 5 studies(3, 7, 10, 16, 29) on relation of insoluble fiber intake and all-cause mortality, 2 study(7, 16) reported an inverse association and other 3(3, 10, 29) reported no significant association. For the association between intake of insoluble fiber and cardiovascular mortality, 4 studies(7, 16, 18, 21) showed an inverse association and the others indicated no significant association.

The association between consumption of insoluble fiber and all-cause mortality was examined in 5 studies (3, 7, 10, 16, 29), including 226,570 participants and 23,990 deaths. Marginal significant inverse association was found (summary RR comparing highest and lowest intakes was 0.85, 95% CI: 0.78,0.93; *I^2^*=79.2%, *P_heterogeneity_* =0.001; Table 2, Supplemental Figure 17). Evidence of heterogeneity between subgroups in stratified analyses was observed for studies stratified by follow-up years (*P*=0.036). (Supplemental Figure 5).

The association between consumption of insoluble fiber and CVD mortality was examined in 6 studies(7, 16, 18, 21, 24, 25), including 289,988 participants and 16,748 deaths. Significant inverse association was found (summary RR comparing highest and lowest intakes was 0.74, 95% CI: 0.69,0.79; *I*^2^=0%, *P_heterogeneity_* =0.99; Table 2, Supplemental Figure 17). No evidence of heterogeneity between subgroups was observed in stratified analyses (*P* >0.05) (Supplemental Figure 5).

For cancer mortality, which was examined in 3 studies(7, 16, 29) with a total of 152,552 participants and 14,043 deaths, no significant association was found; summary RR comparing the highest and lowest intakes was 0.92 (95% CI: 0.74,1.14; *I*^2^=82.9%, *P_heterogeneity_* =0.03; Table 2, Supplemental Figure 17).

### In the sensitivity analysis, no single study substantially changed the statistical significance or direction of the combined RR, except that exclusion of the study by Katagiri et al and Xu et al(16) lead to a non-significant association between insoluble fiber intake and all-cause mortality (Supplemental Table 7).

### *Soluble Fiber*

Of the 5 studies(3, 7, 10, 16, 29) on intake of soluble fiber and all-cause mortality, 3 studies(3, 7, 16) reported an inverse association and other 2(10, 29) reported no significant association. For the association between intake of soluble fiber and cardiovascular mortality, 2 study(16, 21) showed an inverse association and the others indicated no significant association.

Five studies(3, 7, 10, 16, 29) were assessed to estimate risk between intake of soluble fiber intake and all-cause mortality, including 304,905 participants and 41,058 deaths. The summary RR for all-cause mortality comparing the highest and lowest intakes of soluble fiber was 0.91 (95% CI: 0.85,0.97; *I^2^*=66.8%, *P_heterogeneity_* =0.02; Table 2, Supplemental Figure 18). Evidence of heterogeneity between subgroups in stratified analyses was observed for studies stratified by region (*P*=0.018) (Supplemental Figure 6).

The association between consumption of soluble fiber and CVD mortality was examined in 5 studies(16, 18, 21, 24, 25), including 236,818participants and 9,121 deaths. The summary RR for all-cause mortality comparing the highest and lowest intakes of soluble fiber was 0.79 (95% CI: 0.72,0.86; *I*^2^=0%, *P_heterogeneity_* =0.72; Table 2, Supplemental Figure 18). Evidence of heterogeneity between subgroups in stratified analyses was observed for studies stratified by region and whether adjusted for carbohydrate and different dietary fiber measurement (*P*<0.05). (Supplemental Figure 6).

### In the sensitivity analysis, the summary estimate is robust on relationship between soluble fiber intake and CVD mortality, but still unstable for all-cause and cancer mortality (Supplemental Table 8).

#

# Supplementary Figures and Tables

**Supplemental Table 1.** Systematic literature review search terms and strategy.

| **Search terms for PubMed (n=2413), until 25 August 2023** |
| --- |
| #1 “Dietary Fiber”[Mesh] OR “Fiber”[Title/Abstract] OR “Fibre”[Title/Abstract] OR “Fibers”[Title/Abstract] OR “Fibres”[Title/Abstract] |
| #2 “mortality” [Mesh] OR “death” [Mesh] OR “survival” [Mesh] OR “mortality” [Title/Abstract] OR “death*” [Title/Abstract] OR “fatal” [Title/Abstract] OR “surviv*” [Title/Abstract] |
| #3 "prospective studies"[Mesh] OR "cohort studies"[Mesh] OR "longitudinal studies"[Mesh] OR "prospective"[Title/Abstract] OR "longitudinal"[Title/Abstract] OR "cohort"[Title/Abstract] OR "cohorts"[Title/Abstract] OR "follow-up"[Title/Abstract] OR “followed up"[Title/Abstract] |
| #1 AND #2 AND #3 |
| **Search terms for Embase (n=3189), until 25 August 2023** |
| #1 fiber.mp. OR fiber/ OR fibre.mp. OR fibers.mp. OR fibres.mp. |
| #2 mortality/ or death/ or survival/ or mortality.mp. or death.mp. or deaths.mp. or fatal.mp. or survival.mp. or survive.mp. |
| #3 cohort.mp. or prospective study.mp. or prospective study/ or proportional hazards models/ or follow-up.mp. or follow up/ |
| #1 AND #2 AND #3 |
| **Search terms for Web of Science (n=** **2397), until 25 August 2023** |
| TS= ((“Fiber” OR “Fibre” OR “Fibers” OR “Fibres”) AND (“mortality” OR “death” OR “deaths” OR “survival” OR “fatal” OR “survive”) AND ("prospective studies" OR "cohort studies" OR "longitudinal studies" OR "prospective" OR "longitudinal" OR "cohort" OR "cohorts" OR "follow-up" OR “followed up”)) |

## Supplemental Table 2. Assessment of quality of included studies for the association between dietary fiber and mortality.

| **First author (year)** | **Study Selection** | | | | **Adjustment for confounders** | | **Outcome** | | | **Total** |
| --- | --- | --- | --- | --- | --- | --- | --- | --- | --- | --- |
|  | **a** | **b** | **c** | **d** | **e** | **f** | **g** | **h** | **i** |  |
| **All-cause mortality** |  |  |  |  |  |  |  |  |  |  |
| Zhang(2022) | 1 | 1 | 1 | 1 | 1 | 1 | 0 | 1 | 1 | 8 |
| Xu(2022) | 1 | 1 | 1 | 1 | 1 | 0 | 1 | 1 | 1 | 8 |
| You(2022) | 1 | 1 | 1 | 1 | 1 | 0 | 1 | 0 | 0 | 6 |
| Kwon (2022) | 1 | 1 | 1 | 1 | 1 | 1 | 1 | 1 | 0 | 8 |
| Ha (2021) | 1 | 1 | 1 | 1 | 1 | 1 | 1 | 1 | 0 | 8 |
| Ho (2020) | 1 | 1 | 1 | 1 | 1 | 0 | 1 | 1 | 0 | 7 |
| Katagiri (2020) | 1 | 1 | 0 | 1 | 1 | 1 | 1 | 1 | 0 | 7 |
| Partula (2020) | 1 | 1 | 1 | 1 | 1 | 1 | 1 | 1 | 0 | 8 |
| Dominguez (2019) | 0 | 1 | 0 | 1 | 1 | 1 | 1 | 1 | 1 | 7 |
| Chan (2016) | 1 | 1 | 1 | 1 | 1 | 1 | 1 | 1 | 0 | 8 |
| Gopinath (2016) | 0 | 1 | 1 | 1 | 1 | 0 | 1 | 1 | 0 | 6 |
| Xu (2016) | 1 | 1 | 1 | 1 | 1 | 1 | 1 | 1 | 0 | 8 |
| Huang (2015) | 1 | 1 | 1 | 1 | 1 | 1 | 1 | 1 | 0 | 8 |
| Cosiales (2014) | 1 | 1 | 1 | 1 | 1 | 1 | 1 | 1 | 0 | 8 |
| Xu (2014) | 1 | 1 | 1 | 1 | 1 | 1 | 1 | 1 | 0 | 8 |
| Chuang (2012) | 1 | 1 | 1 | 1 | 1 | 1 | 0 | 1 | 0 | 7 |
| Nilsson (2012) | 1 | 1 | 1 | 1 | 1 | 1 | 1 | 1 | 0 | 8 |
| Akbaraly (2011) | 1 | 1 | 1 | 1 | 1 | 1 | 1 | 1 | 0 | 8 |
| Streppel (2008) | 1 | 1 | 1 | 1 | 0 | 0 | 1 | 1 | 0 | 6 |
| Lubin (2003) | 1 | 1 | 1 | 1 | 1 | 0 | 1 | 1 | 0 | 7 |
| Todd (1999) | 1 | 1 | 1 | 1 | 1 | 1 | 1 | 0 | 1 | 8 |
|  |  |  |  |  |  |  |  |  |  |  |
| **Cardiovascular mortality** |  |  |  |  |  |  |  |  |  |  |
| Xu(2022) | 1 | 1 | 1 | 1 | 1 | 0 | 1 | 1 | 1 | 8 |
| Kwon (2022) | 1 | 1 | 1 | 1 | 1 | 1 | 1 | 1 | 0 | 8 |
| Ha (2021) | 1 | 1 | 1 | 1 | 1 | 1 | 1 | 1 | 0 | 8 |
| Miyazawa (2020) | 1 | 1 | 1 | 1 | 1 | 0 | 1 | 1 | 0 | 7 |
| Katagiri (2020) | 1 | 1 | 0 | 1 | 1 | 1 | 1 | 1 | 0 | 7 |
| Huang (2015) | 1 | 1 | 1 | 1 | 1 | 1 | 1 | 1 | 0 | 8 |
| Xu (2014) | 1 | 1 | 1 | 1 | 1 | 1 | 1 | 1 | 0 | 8 |
| Cosiales (2014) | 1 | 1 | 1 | 1 | 1 | 1 | 1 | 1 | 0 | 8 |
| Threapleton (2012) | 1 | 1 | 1 | 1 | 1 | 0 | 1 | 1 | 0 | 7 |
| Crowe (2012) | 1 | 1 | 0 | 1 | 1 | 1 | 1 | 1 | 0 | 7 |
| Akbaraly(2011) | 1 | 1 | 1 | 1 | 1 | 1 | 1 | 1 | 0 | 8 |
| Eshak (2010) | 1 | 1 | 1 | 1 | 1 | 1 | 1 | 1 | 0 | 8 |
| Buyken (2010) | 1 | 1 | 1 | 1 | 1 | 0 | 1 | 1 | 1 | 8 |
| Baer (2010) | 1 | 1 | 1 | 1 | 1 | 1 | 0 | 1 | 0 | 7 |
| Kaushik (2009) | 1 | 1 | 1 | 1 | 1 | 0 | 1 | 1 | 0 | 7 |
| Streppel (2008) | 1 | 1 | 1 | 1 | 0 | 0 | 1 | 1 | 0 | 6 |
| Mozaffarian (2003) | 1 | 1 | 1 | 1 | 1 | 1 | 1 | 1 | 0 | 8 |
| Liu (2002) | 1 | 1 | 1 | 1 | 1 | 1 | 1 | 1 | 0 | 8 |
| Pietinen (1996) | 1 | 1 | 1 | 1 | 1 | 1 | 1 | 1 | 0 | 8 |
| KHAW (1987) | 1 | 1 | 1 | 1 | 1 | 0 | 1 | 1 | 0 | 7 |
|  |  |  |  |  |  |  |  |  |  |  |
| **Cancer mortality** |  |  |  |  |  |  |  |  |  |  |
| Xu(2022) | 1 | 1 | 1 | 1 | 1 | 0 | 1 | 1 | 1 | 8 |
| Ha (2021) | 1 | 1 | 1 | 1 | 1 | 1 | 1 | 1 | 0 | 8 |
| Katagiri (2020) | 1 | 1 | 0 | 1 | 1 | 1 | 1 | 1 | 0 | 7 |
| Chan (2016) | 1 | 1 | 1 | 1 | 1 | 1 | 1 | 1 | 0 | 8 |
| Xu (2016) | 1 | 1 | 1 | 1 | 1 | 1 | 1 | 1 | 0 | 8 |
| Huang (2015) | 1 | 1 | 1 | 1 | 1 | 1 | 1 | 1 | 0 | 8 |
| Cosiales (2014) | 1 | 1 | 1 | 1 | 1 | 1 | 1 | 1 | 0 | 8 |
| Xu (2014) | 1 | 1 | 1 | 1 | 1 | 1 | 1 | 1 | 0 | 8 |
| Chuang (2012) | 1 | 1 | 1 | 1 | 1 | 1 | 0 | 1 | 0 | 7 |
| Akbaraly(2011) | 1 | 1 | 1 | 1 | 1 | 1 | 1 | 1 | 0 | 8 |
| Baer (2010) | 1 | 1 | 1 | 1 | 1 | 1 | 0 | 1 | 0 | 7 |

a. Representativeness of the exposed cohort;

b. Selection of the non-exposed cohort;

c. Ascertainment of exposure;

d. Demonstration that outcome of interest was not present at start of study;

e. Comparability of cohorts on the basis of the design or analysis (adjusted for the most important factor);

f. Comparability of cohorts on the basis of the design or analysis (adjusted for a second important factor);

g. Assessment of outcome;

h. Was follow-up long enough for outcomes to occur;

i. Adequacy of follow-up of cohorts.


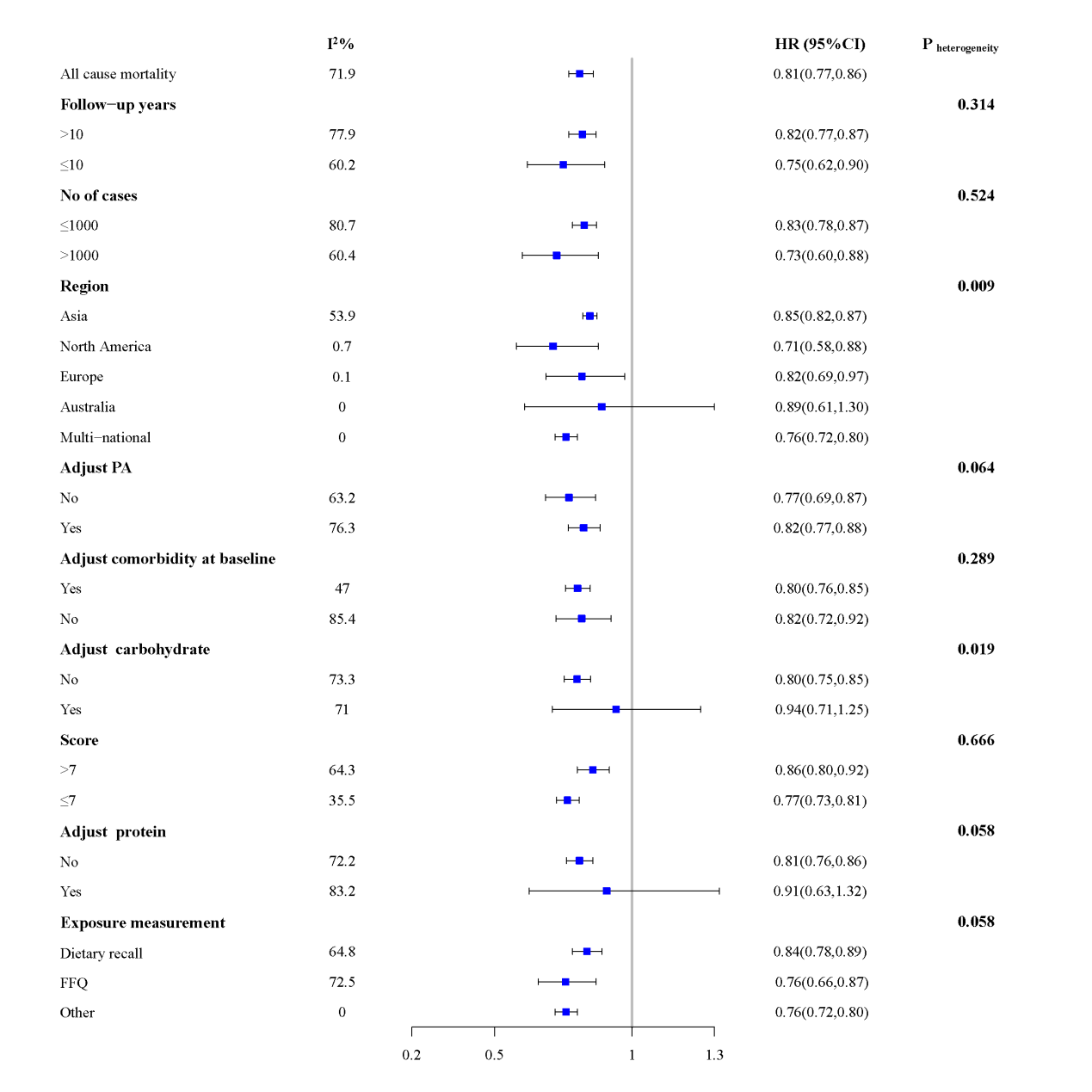

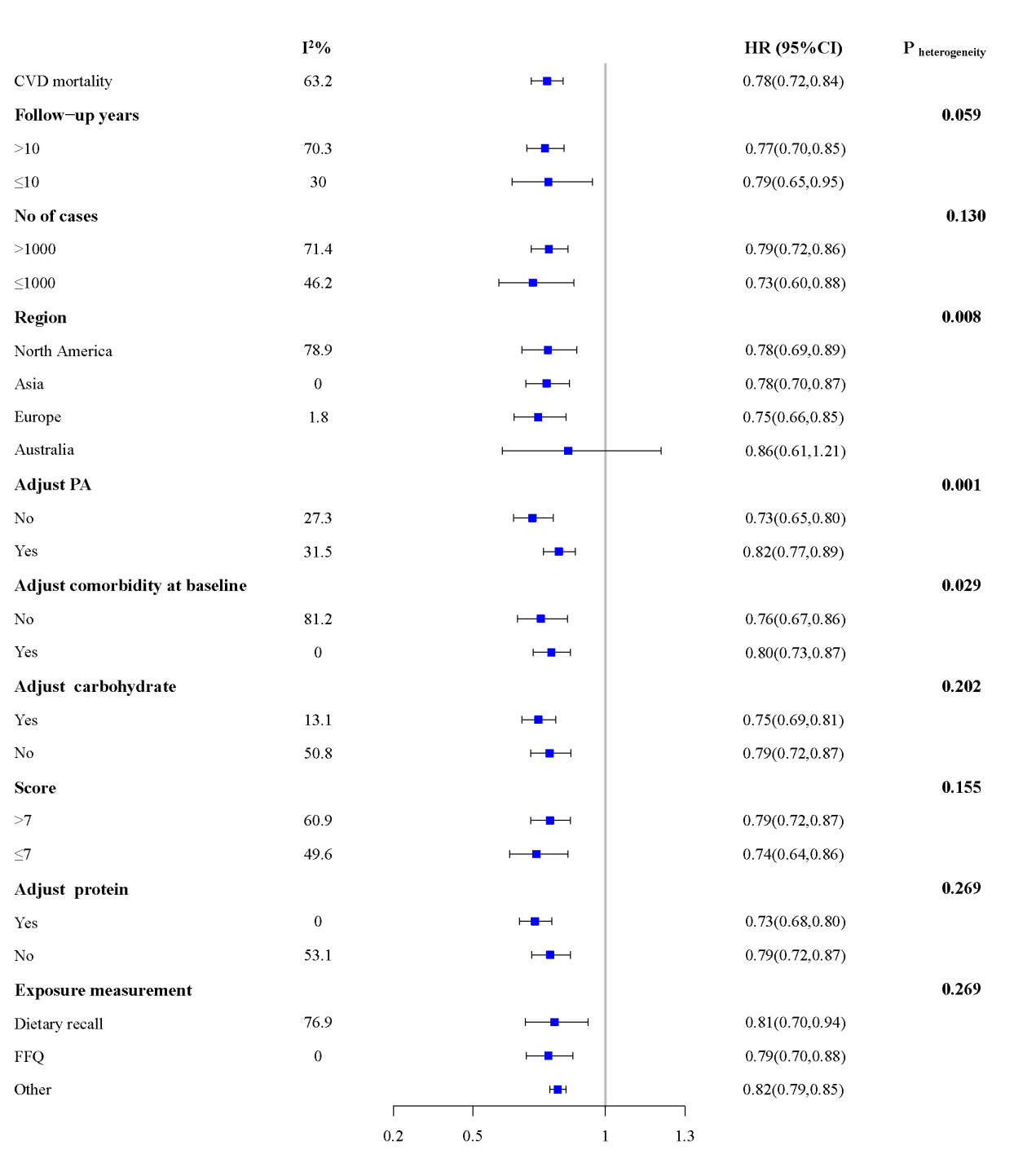


A

B


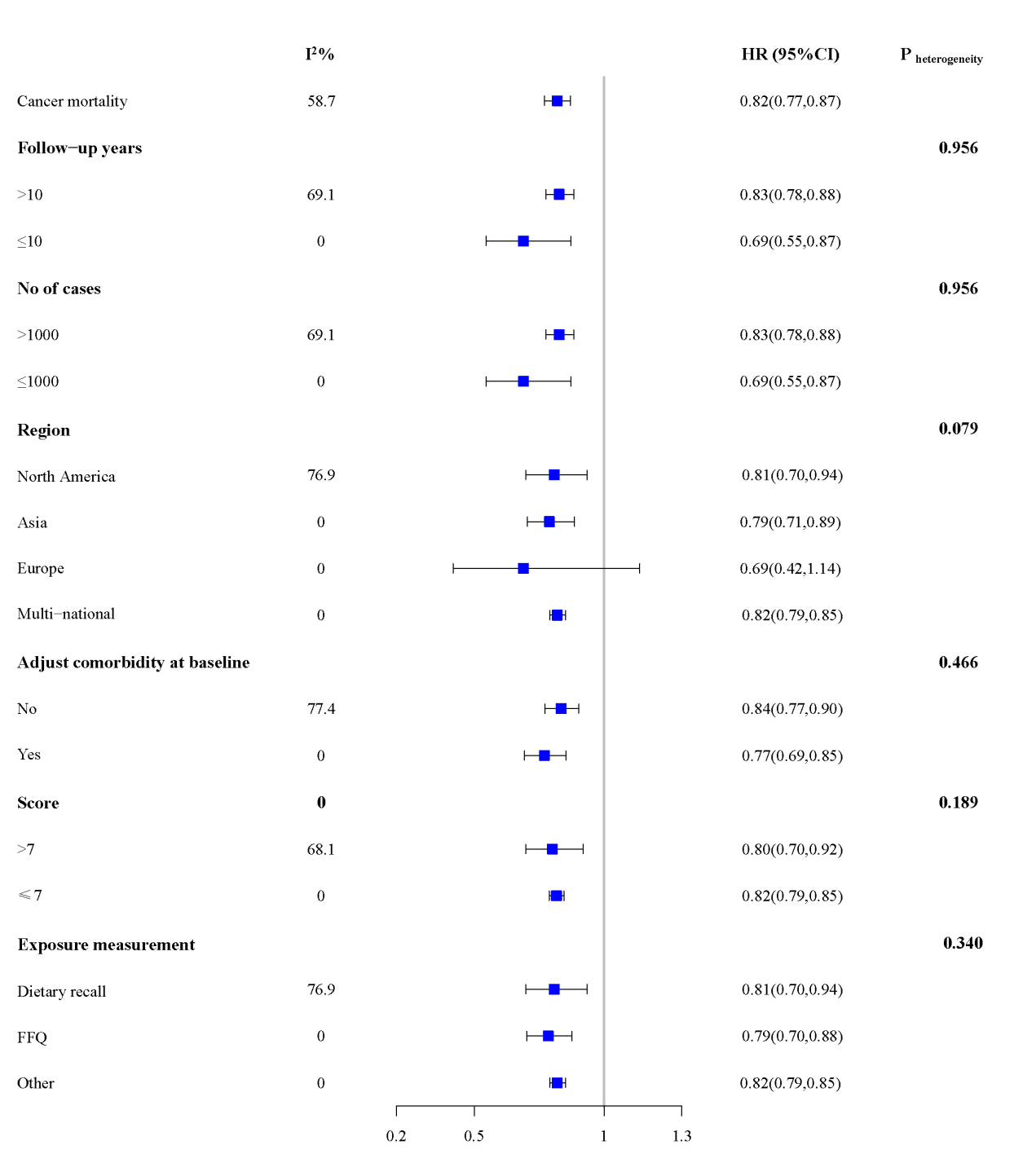


C

**Supplemental Figure 1.** Subgroup analysis of dietary fiber intake and risk of all cause mortality (A), CVD mortality (B) and cancer (C) mortality for the highest versus lowest meta-analysis.**Supplemental Table 3.** Sensitivity analysis of dietary fiber intake and risk of all cause, CVD and cancer mortality for the highest versus lowest meta-analysis.

| Study omitted | | RR | | | | 95% CI | | |
| --- | --- | --- | --- | --- | --- | --- | --- | --- |
| **All cause mortality** | |  | |  | | |  | |
| Xu(2022) | | 0.81 | | 0.76 | | | 0.86 |  |
| You(2022) | | 0.82 | | 0.78 | | | 0.86 |  |
| Zhang(2022) | | 0.80 | | 0.76 | | | 0.85 |  |
| Kwon (2022) | | 0.82 | | 0.77 | | | 0.86 |  |
| Ha (2021) | | 0.81 | | 0.76 | | | 0.86 |  |
| Katagiri (2020) | | 0.81 | | 0.76 | | | 0.87 |  |
| Partula (2020) | | 0.81 | | 0.76 | | | 0.85 |  |
| Ho (2020) | | 0.82 | | 0.77 | | | 0.87 |  |
| Dominguez (2019) | | 0.82 | | 0.77 | | | 0.86 |  |
| Gopinath (2016) | | 0.81 | | 0.77 | | | 0.86 |  |
| Xu (2016) | | 0.80 | | 0.75 | | | 0.86 |  |
| Cosiales (2014) | | 0.82 | | 0.77 | | | 0.86 |  |
| Nilsson(2012) | | 0.80 | | 0.76 | | | 0.84 |  |
| Chuang (2012) | | 0.82 | | 0.77 | | | 0.87 |  |
| Lubin (2003) | | 0.82 | | 0.77 | | | 0.86 |  |
| Todd (1999) | | 0.82 | | 0.77 | | | 0.86 |  |
|  | |  | |  | | |  |  |
| **CVD mortality** | |  | |  | | |  |  |
| Xu(2022) | | 0.78 | | 0.72 | | | 0.85 |  |
| Zhang(2022) | | 0.78 | | 0.72 | | | 0.85 |  |
| Kwon (2022) | | 0.78 | | 0.72 | | | 0.84 |  |
| Ha (2021) | | 0.76 | | 0.70 | | | 0.83 |  |
| Katagiri (2020) | | 0.77 | | 0.70 | | | 0.84 |  |
| Miyazawa (2020) | | 0.78 | | 0.72 | | | 0.85 |  |
| Xu (2016) | | 0.76 | | 0.71 | | | 0.81 |  |
| Cosiales (2014) | | 0.78 | | 0.72 | | | 0.84 |  |
| Crowe (2012) | | 0.77 | | 0.71 | | | 0.85 |  |
| Buyken (2010) | | 0.77 | | 0.71 | | | 0.84 |  |
| Eshak (2010) | | 0.77 | | 0.71 | | | 0.84 |  |
| Liu (2002) | | 0.77 | | 0.71 | | | 0.84 |  |
| Pietinen(1996) | | 0.78 | | 0.72 | | | 0.85 |  |
| KHAW (1987) | | 0.78 | | 0.73 | | | 0.85 |  |
| **Cancer mortality** | |  | |  | | |  |  |
| Xu(2022) | | 0.83 | | 0.77 | | | 0.89 |  |
| Ha (2021) | | 0.83 | | 0.78 | | | 0.88 |  |
| Katagiri (2020) | | 0.82 | | 0.76 | | | 0.88 |  |
| Xu (2016) | | 0.81 | | 0.78 | | | 0.84 |  |
| Cosiales (2014) | | 0.82 | | 0.77 | | | 0.87 |  |
| Chuang (2012) | | 0.80 | | 0.73 | | | 0.89 |  |
|  | |  | |  | | |  |  |

**
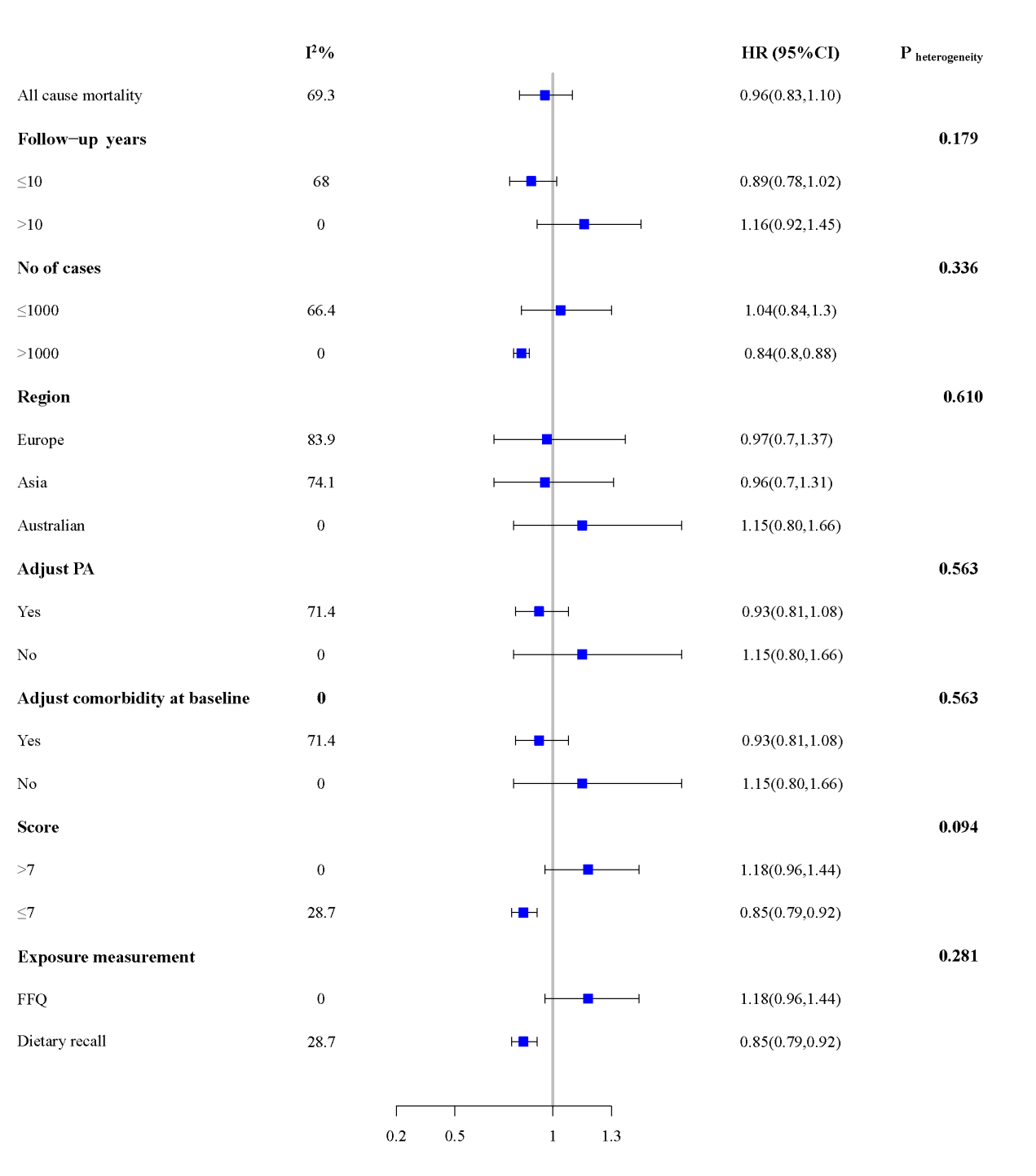
**

A


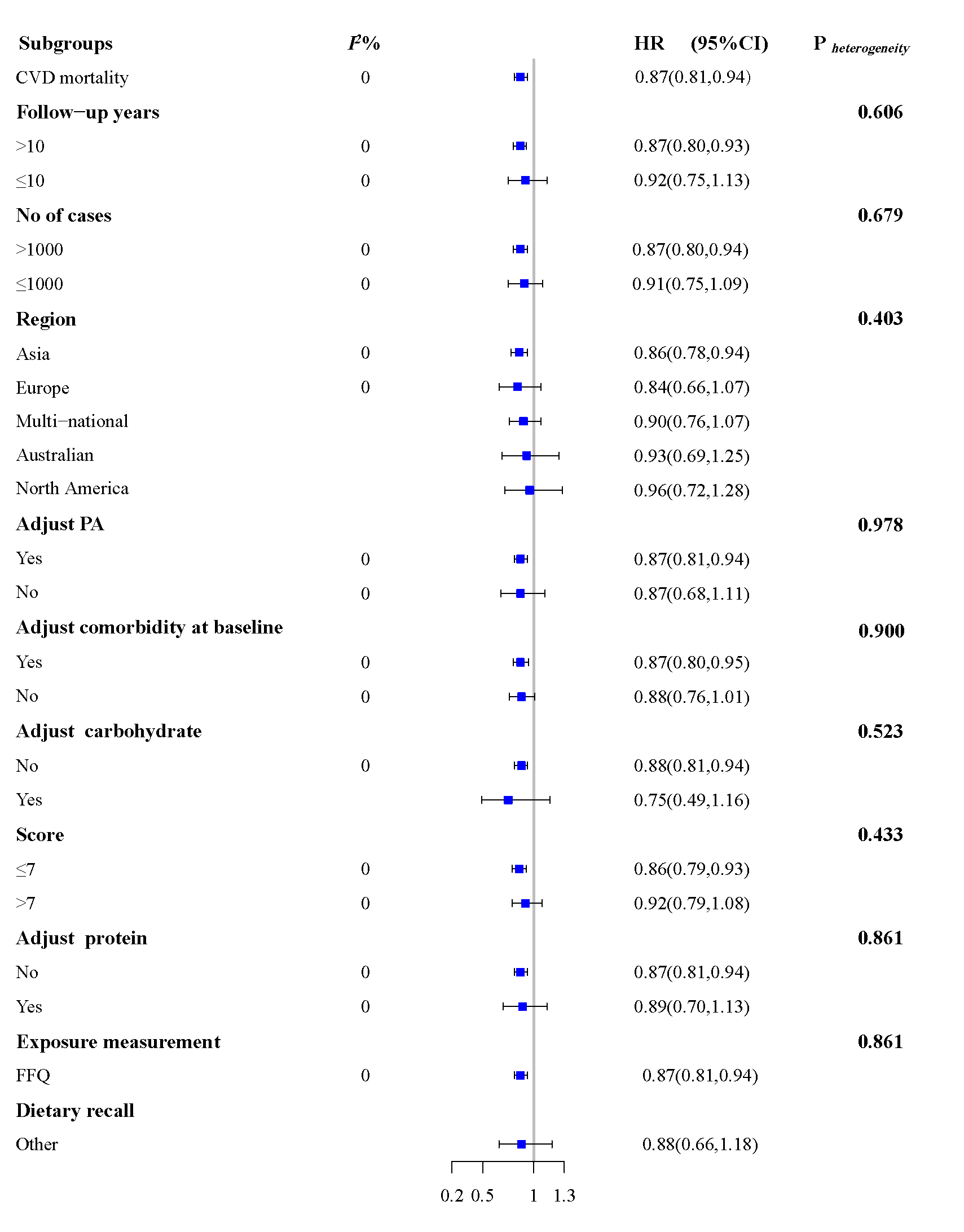


**Supplemental Figure 2.** Subgroup analysis of vegetable fiber intake and risk of all cause mortality (A) and CVD mortality (B) for the highest versus lowest meta-analysis.**Supplemental Table 4.** Sensitivity analysis of vegetable fiber intake and risk of all cause mortality and CVD mortality for the highest versus lowest meta-analysis.

B

| **Study omitted** | | **RR** | | | | **95% CI** | | |
| --- | --- | --- | --- | --- | --- | --- | --- | --- |
| **All cause mortality** | |  | |  | | |  | |
| Zhang(2022) | | 0.90 | | 0.80 | | | 1.02 |  |
| Partula(2020) | | 0.92 | | 0.80 | | | 1.05 |  |
| Katagiri(2020) | | 1.04 | | 0.84 | | | 1.29 |  |
| Dominguez(2019) | | 1.04 | | 0.82 | | | 1.32 |  |
| Gopinath(2016) | | 0.93 | | 0.81 | | | 1.08 |  |
|  | |  | |  | | |  |  |
| **CVD mortality** | |  | |  | | |  |  |
| Katagiri(2020) | | 0.90 | | 0.81 | | | 1.01 |  |
| Threapleton(2012)women | | 0.88 | | 0.81 | | | 0.94 |  |
| Crowe(2012) | | 0.87 | | 0.8 | | | 0.94 |  |
| Buyken(2010) | | 0.87 | | 0.81 | | | 0.93 |  |
| Eshak(2010) | | 0.87 | | 0.81 | | | 0.94 |  |
| Liu(2002)women | | 0.87 | | 0.80 | | | 0.93 |  |
| Pietinen(1996)men | | 0.87 | | 0.81 | | | 0.94 |  |

**
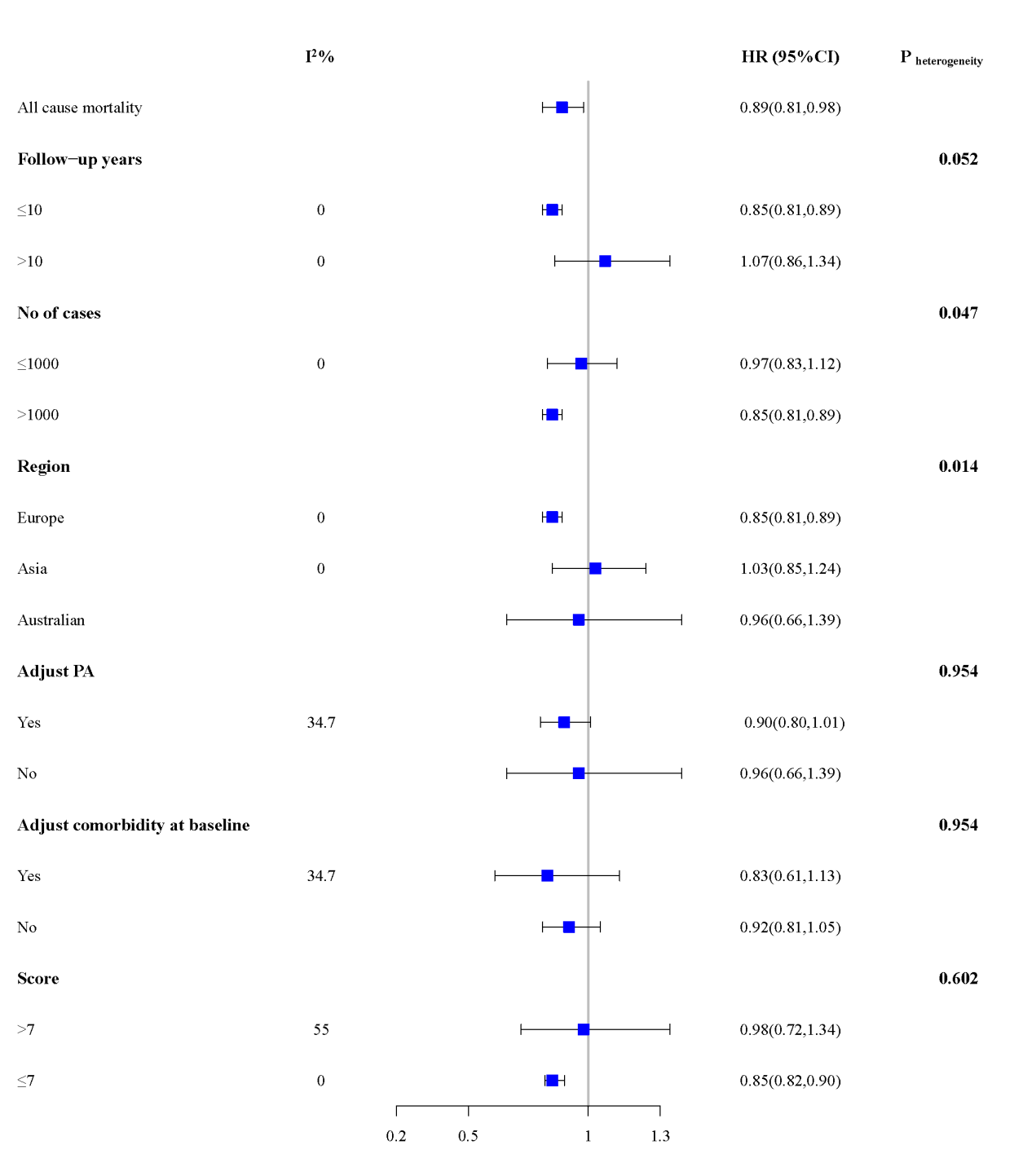
 Supplemental Figure 3.** Subgroup analysis of fruit fiber intake and risk of all cause mortality (A) and CVD mortality(B) for the highest versus lowest meta-analysis.

A


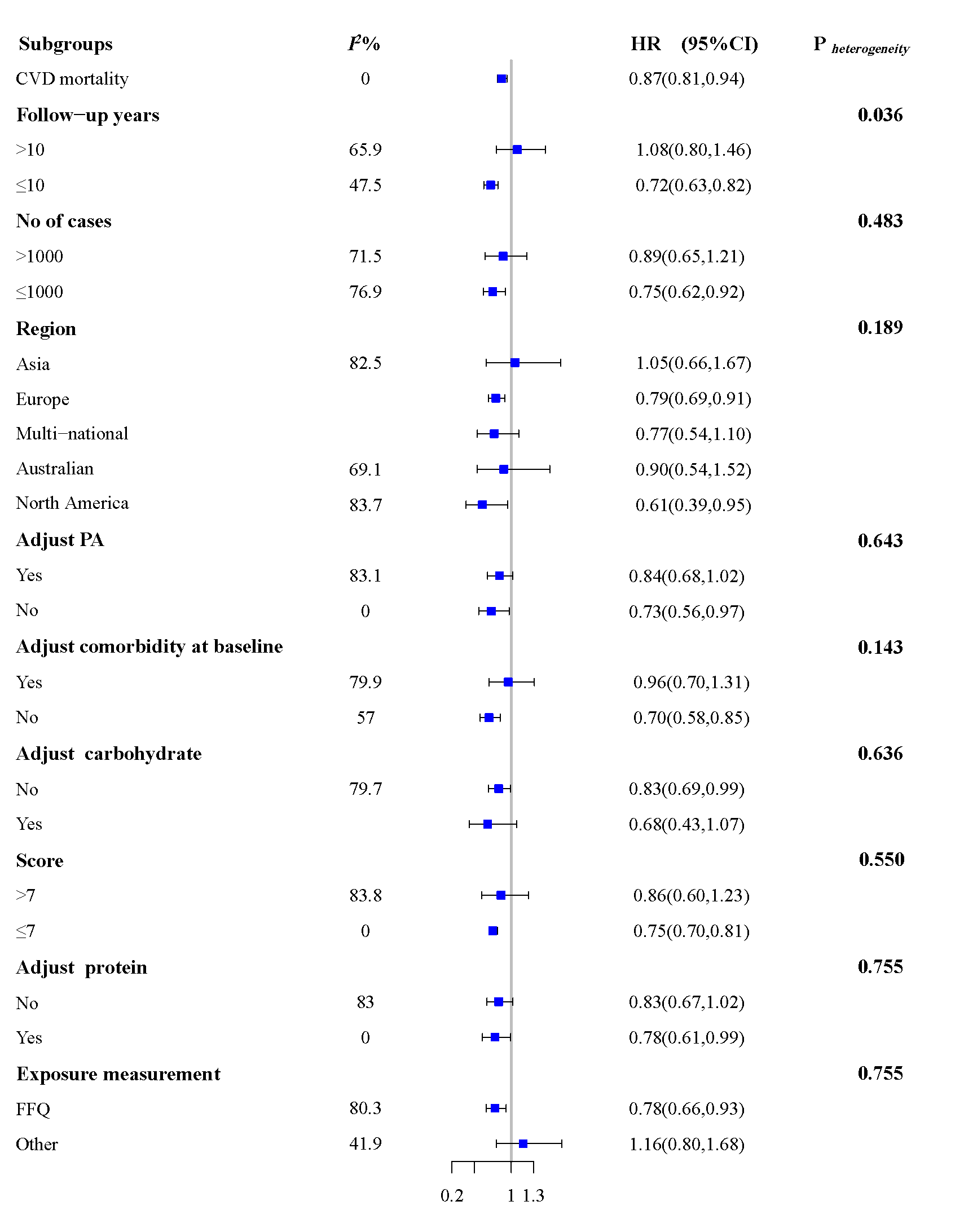


**Supplemental Figure 2.** Sensitivity analysis of fruit fiber intake and risk of all cause mortality, CVD mortality and cancer mortality for the highest versus lowest meta-analysis.

B

| **Study omitted** | | **RR** | | | | **95% CI** | | |
| --- | --- | --- | --- | --- | --- | --- | --- | --- |
| **All cause mortality** | |  | |  | | |  | |
| Zhang(2022) | | 0.92 | | 0.81 | | | 1.05 |  |
| Partula(2020) | | 0.85 | | 0.82 | | | 0.89 |  |
| Katagiri(2020) | | 0.97 | | 0.83 | | | 1.12 |  |
| Dominguez(2019) | | 0.90 | | 0.79 | | | 1.02 |  |
| Gopinath(2016) | | 0.90 | | 0.80 | | | 1.01 |  |
|  | |  | |  | | |  |  |
| **CVD mortality** | |  | |  | | |  |  |
| Katagiri(2020) | | 0.82 | | 0.71 | | | 0.96 |  |
| Threapleton(2012)women | | 0.84 | | 0.71 | | | 0.99 |  |
| Crowe(2012) | | 0.84 | | 0.72 | | | 0.98 |  |
| Buyken(2010) | | 0.81 | | 0.69 | | | 0.95 |  |
| Eshak(2010) | | 0.87 | | 0.78 | | | 0.97 |  |
| Mozaffarian (2003) | | 0.87 | | 0.78 | | | 0.97 |  |
| Liu(2002)women | | 0.84 | | 0.71 | | | 0.98 |  |
| Pietinen(1996)men | | 0.83 | | 0.72 | | | 0.95 |  |

A


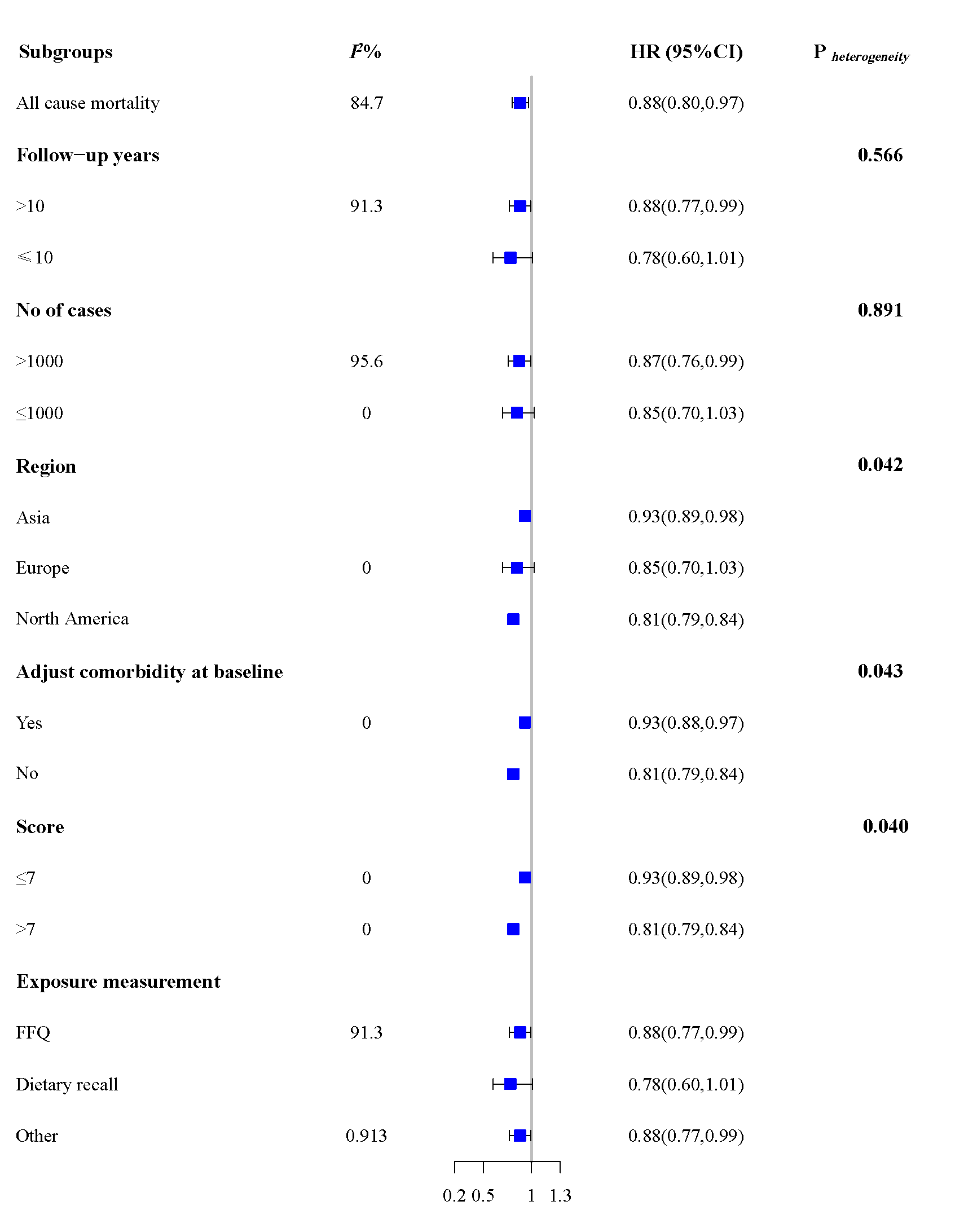

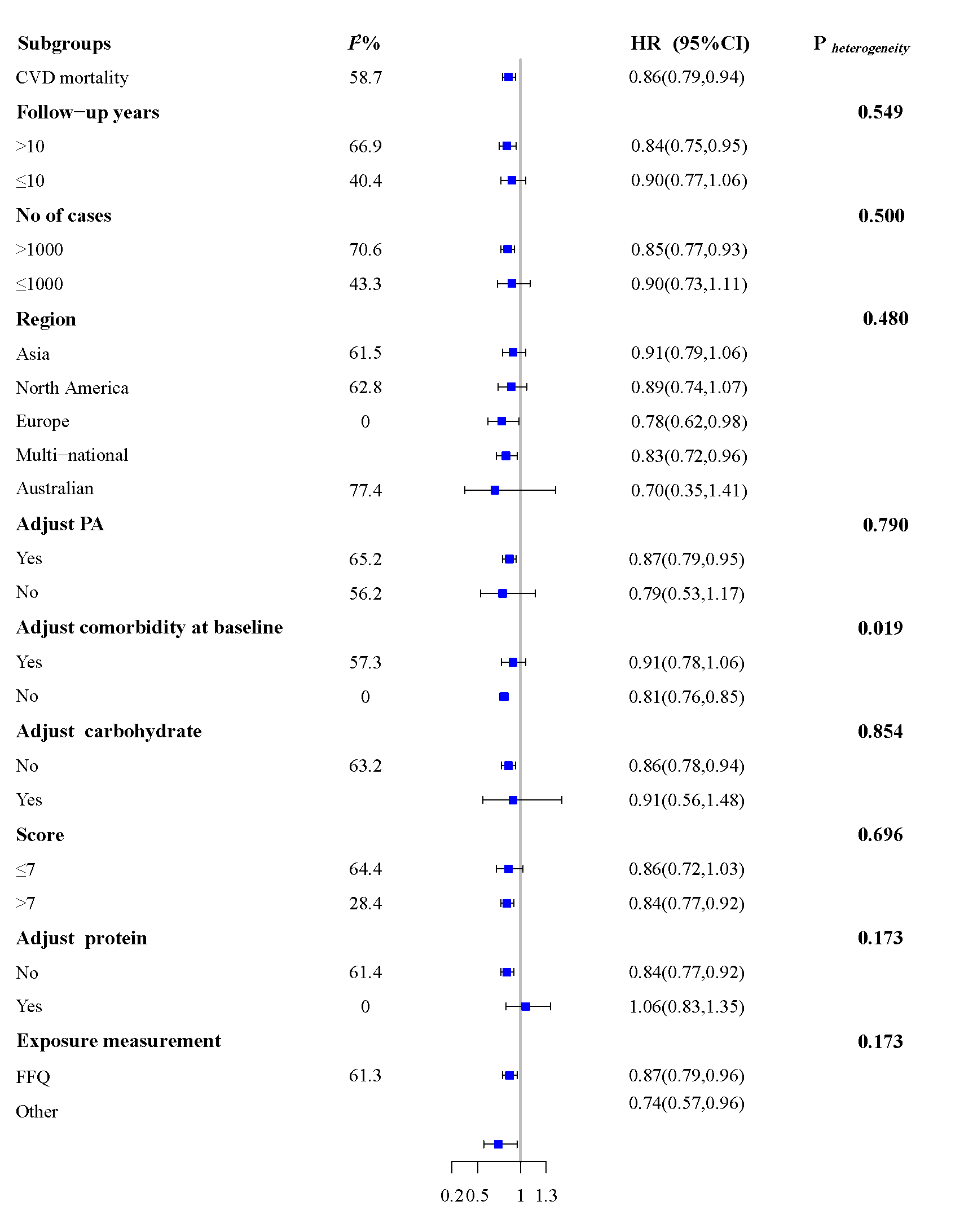


**Supplemental Figure 4.** Subgroup analysis of cereal fiber intake and risk of all cause mortality (A), CVD and mortality (A) for the highest versus lowest meta-analysis.

B

**Supplemental Table 6.** Sensitivity analysis of cereal fiber intake and risk of all cause mortality, and CVD mortality for the highest versus lowest meta-analysis.

| **Study omitted** | | **RR** | | | | **95% CI** | | |
| --- | --- | --- | --- | --- | --- | --- | --- | --- |
| **All cause mortality** | |  | |  | | |  | |
| Katagiri(2020) | | 0.81 | | 0.79 | | | 0.84 |  |
| Partula(2020) | | 0.88 | | 0.77 | | | 0.99 |  |
| Dominguez(2019) | | 0.85 | | 0.76 | | | 0.96 |  |
| Huang(2015) | | 0.92 | | 0.88 | | | 0.97 |  |
|  | |  | |  | | |  |  |
| **CVD mortality** | |  | |  | | |  |  |
| Katagiri(2020) | | 0.83 | | 0.77 | | | 0.90 |  |
| Huang(2015) | | 0.88 | | 0.8 | | | 0.97 |  |
| Threapleton(2012)women | | 0.86 | | 0.78 | | | 0.94 |  |
| Crowe(2012) | | 0.87 | | 0.78 | | | 0.96 |  |
| Buyken(2010) | | 0.85 | | 0.78 | | | 0.94 |  |
| Eshak(2010) | | 0.87 | | 0.78 | | | 0.96 |  |
| Kaushik(2009) | | 0.87 | | 0.8 | | | 0.95 |  |
| Dariush(2003) | | 0.86 | | 0.78 | | | 0.95 |  |
| Liu(2002)women | | 0.85 | | 0.77 | | | 0.92 |  |
| Pietinen(1996)men | | 0.87 | | 0.79 | | | 0.96 |  |

**
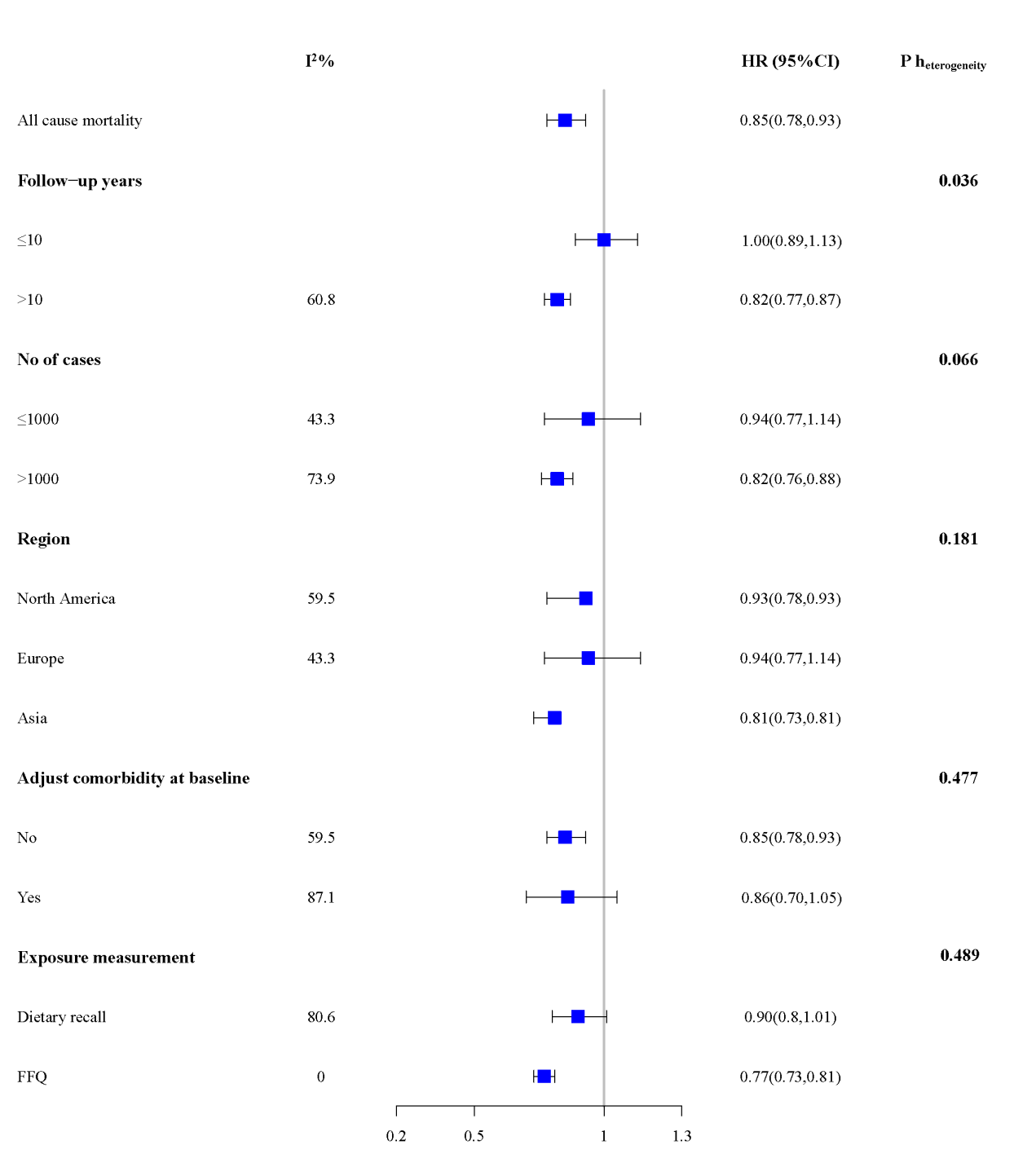
** **
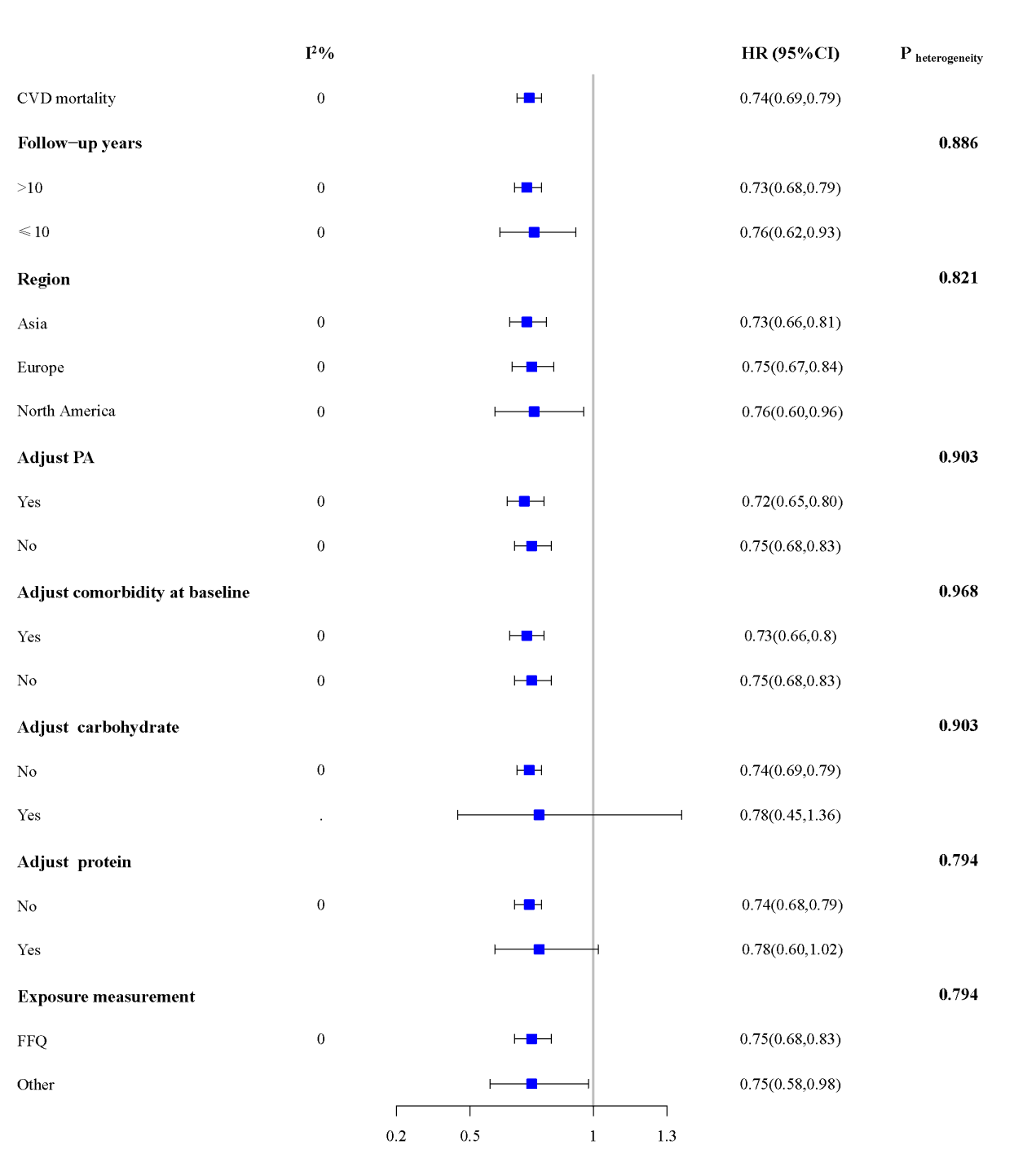
**

A

**Supplemental Figure 5.** Subgroup analysis of insoluble fiber intake and risk of all cause mortality (A) and CVD mortality (B) for the highest versus lowest meta-analysis.

B

**Supplemental Table 7.** Sensitivity analysis of insoluble fiber intake and risk of all cause mortality and CVD mortality for the highest versus lowest meta-analysis.

| **Study omitted** | | **RR** | | | | **95% CI** | | |
| --- | --- | --- | --- | --- | --- | --- | --- | --- |
| **All cause mortality** | |  | |  | | |  | |
| Xu(2022) | | 0.87 | | 0.75 | | | 1.00 |  |
| Partula(2020) | | 0.82 | | 0.77 | | | 0.87 |  |
| Katagiri(2020) | | 0.88 | | 0.8 | | | 0.98 |  |
| Dominguez(2019) | | 0.86 | | 0.78 | | | 0.94 |  |
| Chan(2016) | | 0.84 | | 0.76 | | | 0.93 |  |
|  | |  | |  | | |  |  |
| **CVD mortality** | |  | |  | | |  |  |
| Xu(2022) | | 0.75 | | 0.68 | | | 0.83 |  |
| Katagiri(2020) | | 0.74 | | 0.68 | | | 0.80 |  |
| Threapleton(2012) women | | 0.74 | | 0.69 | | | 0.79 |  |
| Eshak(2010) | | 0.73 | | 0.67 | | | 0.79 |  |
| Liu(2002) women | | 0.74 | | 0.68 | | | 0.79 |  |
| Pietinen(1996)men | | 0.74 | | 0.68 | | | 0.79 |  |
|  | |  | |  | | |  |  |
| **Cancer mortality** | |  | |  | | |  |  |
| Xu(2022) | | 1.03 | | 0.92 | | | 1.17 |  |
| Katagiri(2020) | | 0.81 | | 0.72 | | | 0.91 |  |
| Chan(2016) | | 0.91 | | 0.68 | | | 1.22 |  |

**
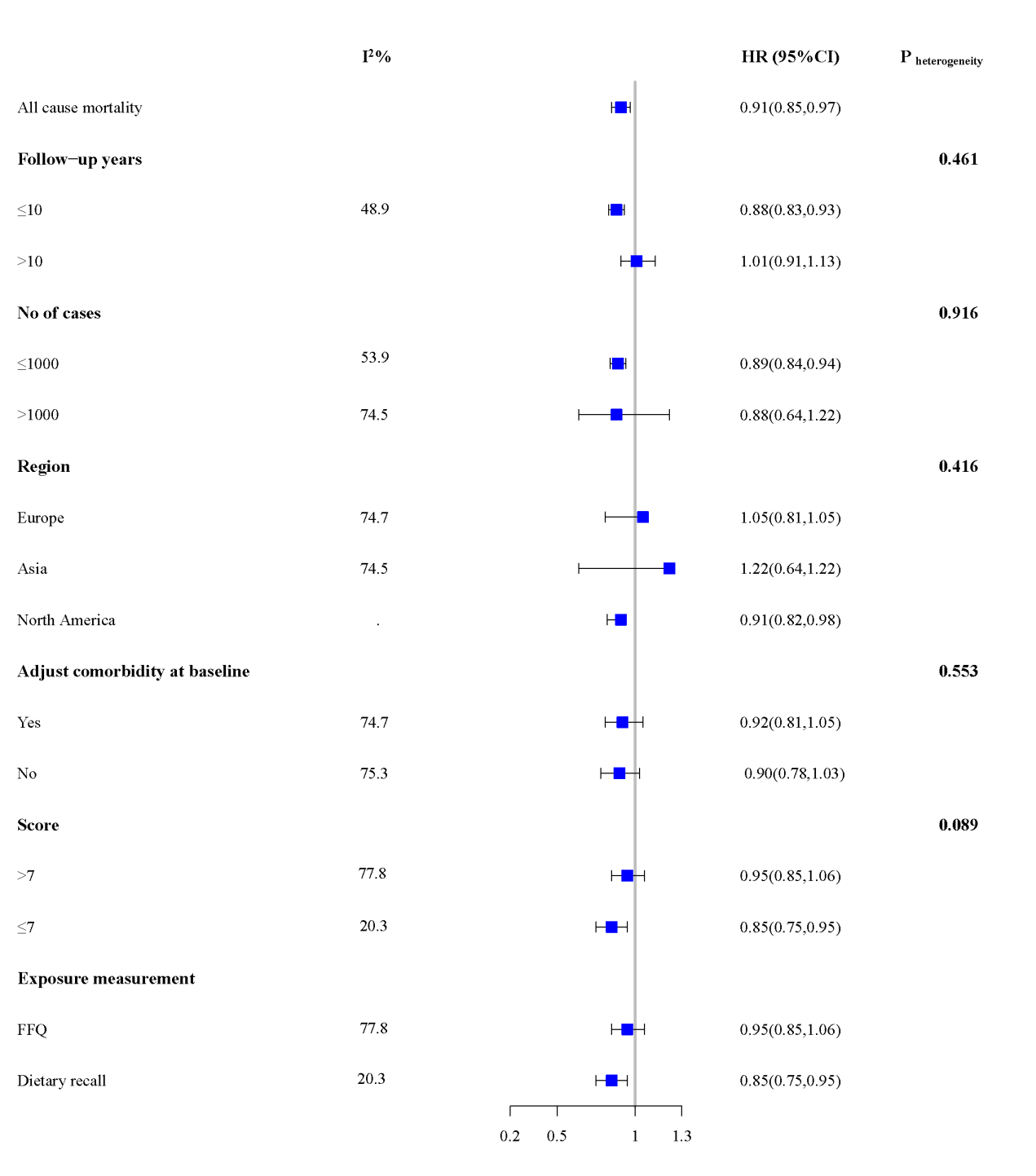
** **
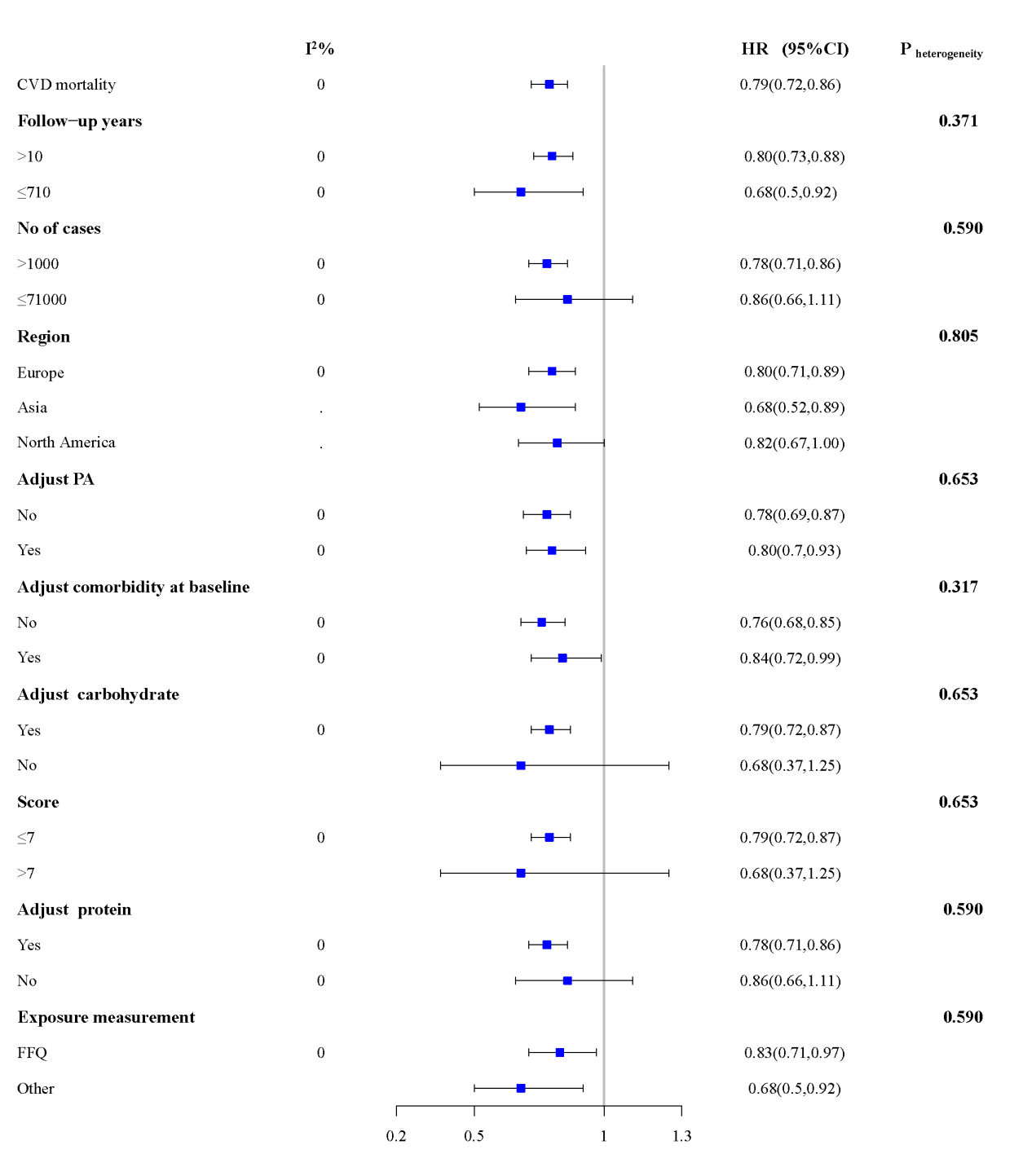
**

A

**Supplemental Figure 6.** Subgroup analysis of soluble fiber intake and risk of all cause (A) mortality and CVD mortality (B) for the highest versus lowest meta-analysis.

B

**Supplemental Table 8.** Sensitivity analysis of soluble fiber intake and risk of all cause mortality and CVD mortality for the highest versus lowest meta-analysis.

| **Study omitted** | | **RR** | | | | **95% CI** | | |
| --- | --- | --- | --- | --- | --- | --- | --- | --- |
| **All cause mortality** | |  | |  | | |  | |
| Xu (2022) | | 0.92 | | 0.82 | | | 1.03 |  |
| Partula (2020) | | 0.88 | | 0.83 | | | 0.93 |  |
| Katagiri( 2020) | | 0.92 | | 0.83 | | | 1.03 |  |
| Dominguez (2019) | | 0.91 | | 0.85 | | | 0.98 |  |
| Chan (2016) | | 0.89 | | 0.83 | | | 0.95 |  |
|  | |  | |  | | |  |  |
| **CVD mortality** | |  | |  | | |  |  |
| Xu (2022) | | 0.80 | | 0.69 | | | 0.92 |  |
| Threapleton (2012)women | | 0.79 | | 0.72 | | | 0.87 |  |
| Eshak (2010) | | 0.78 | | 0.70 | | | 0.86 |  |
| Liu (2002) women | | 0.78 | | 0.70 | | | 0.85 |  |
| Pietinen (1996)men | | 0.80 | | 0.73 | | | 0.88 |  |


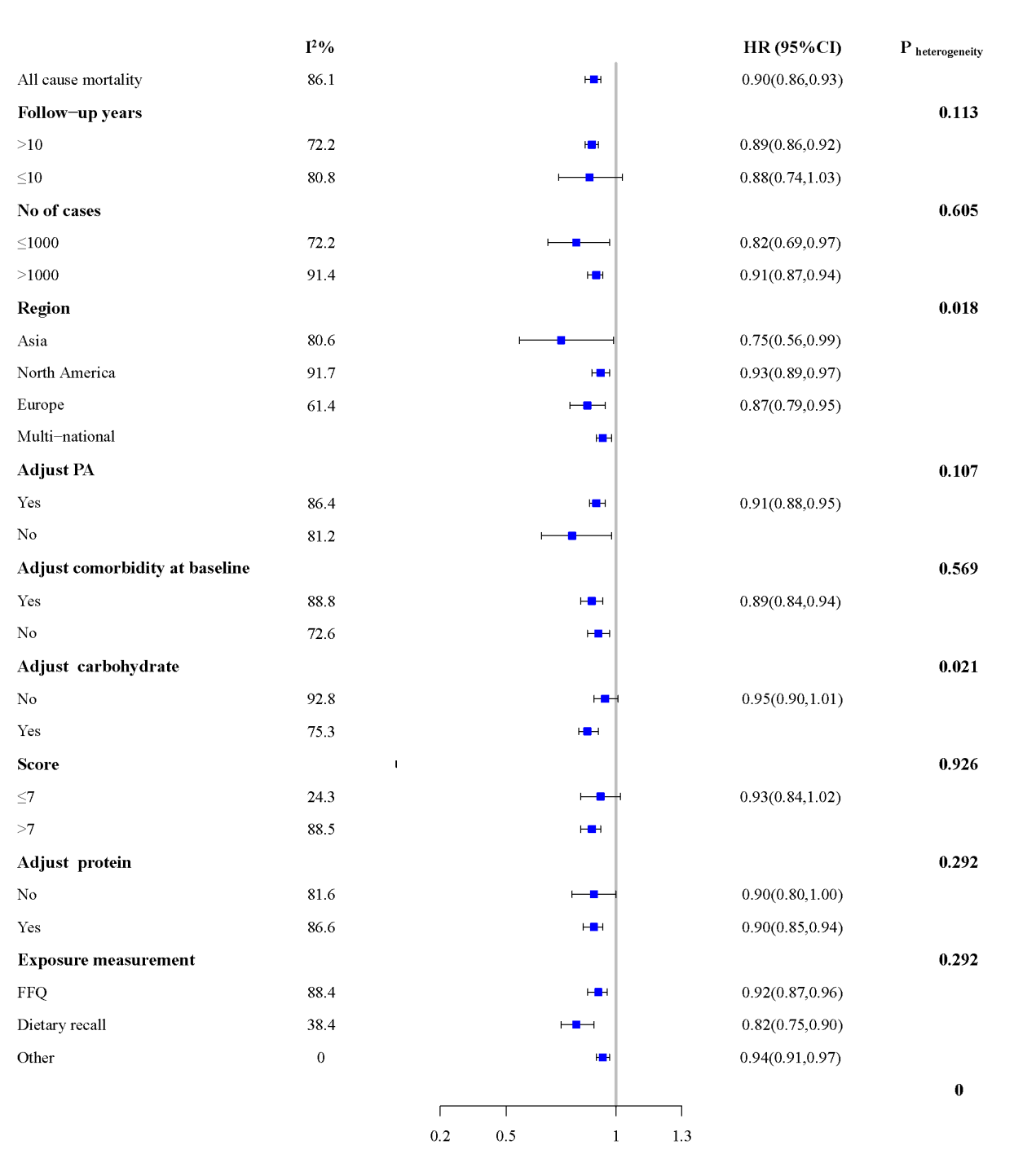

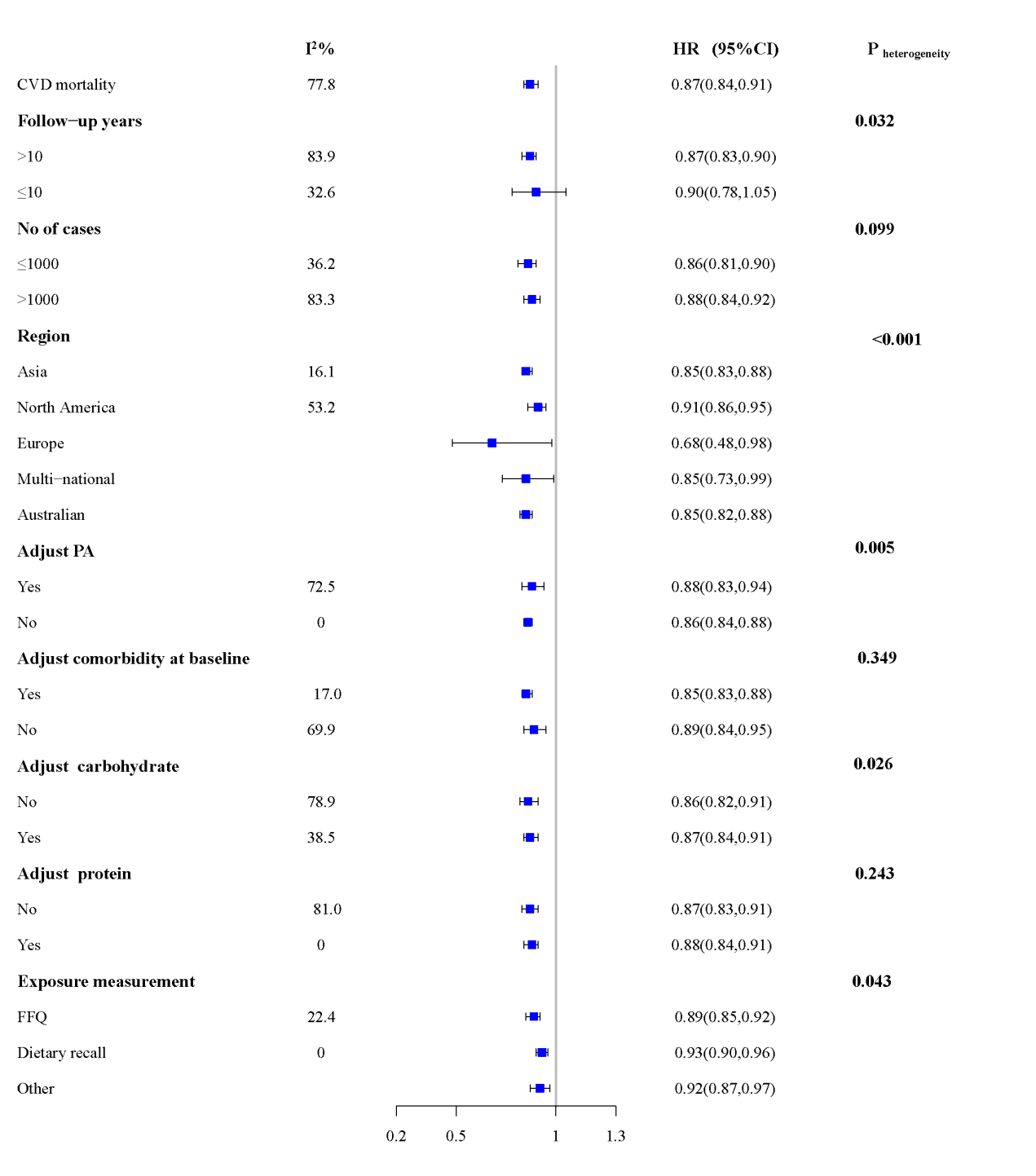

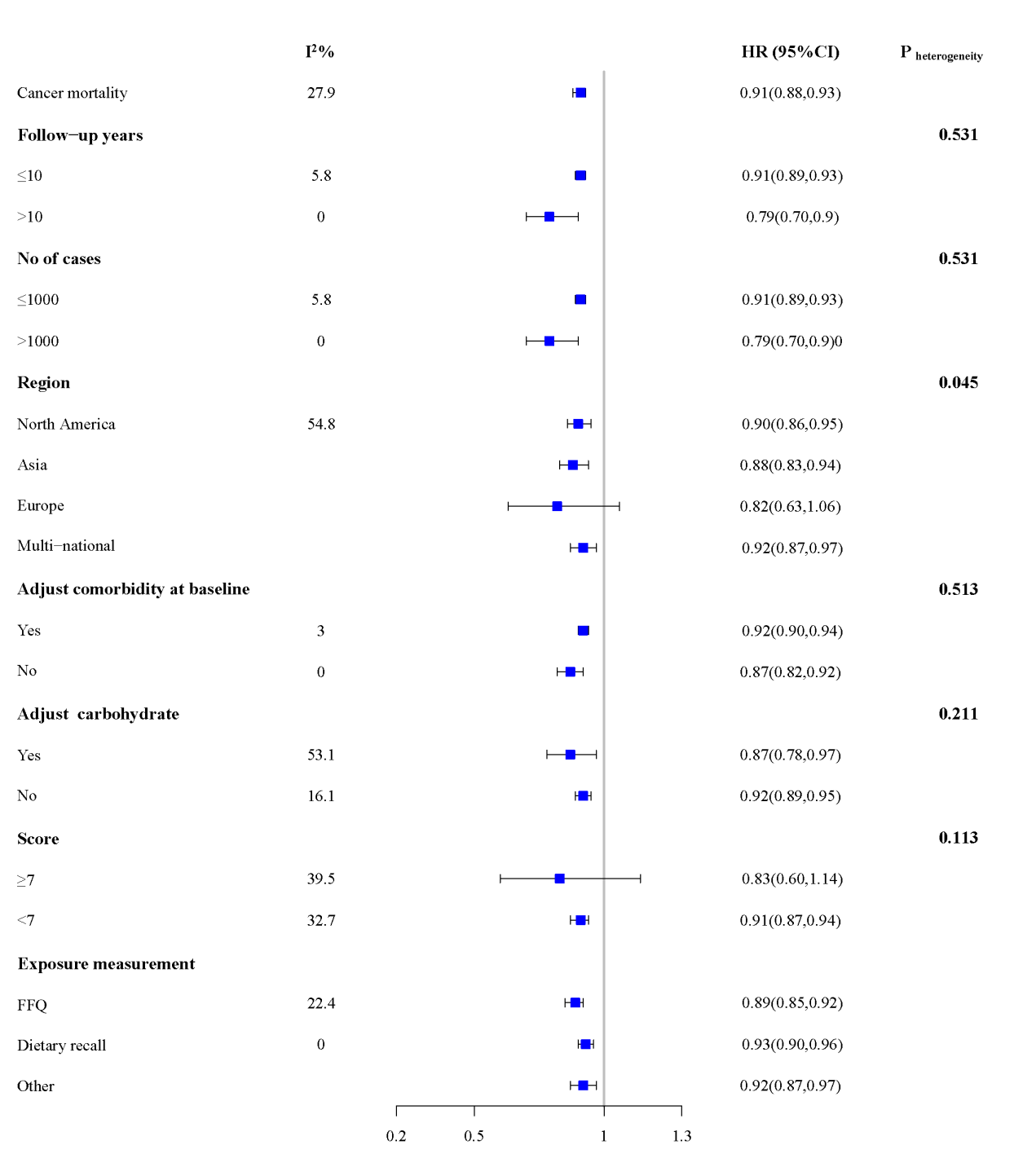

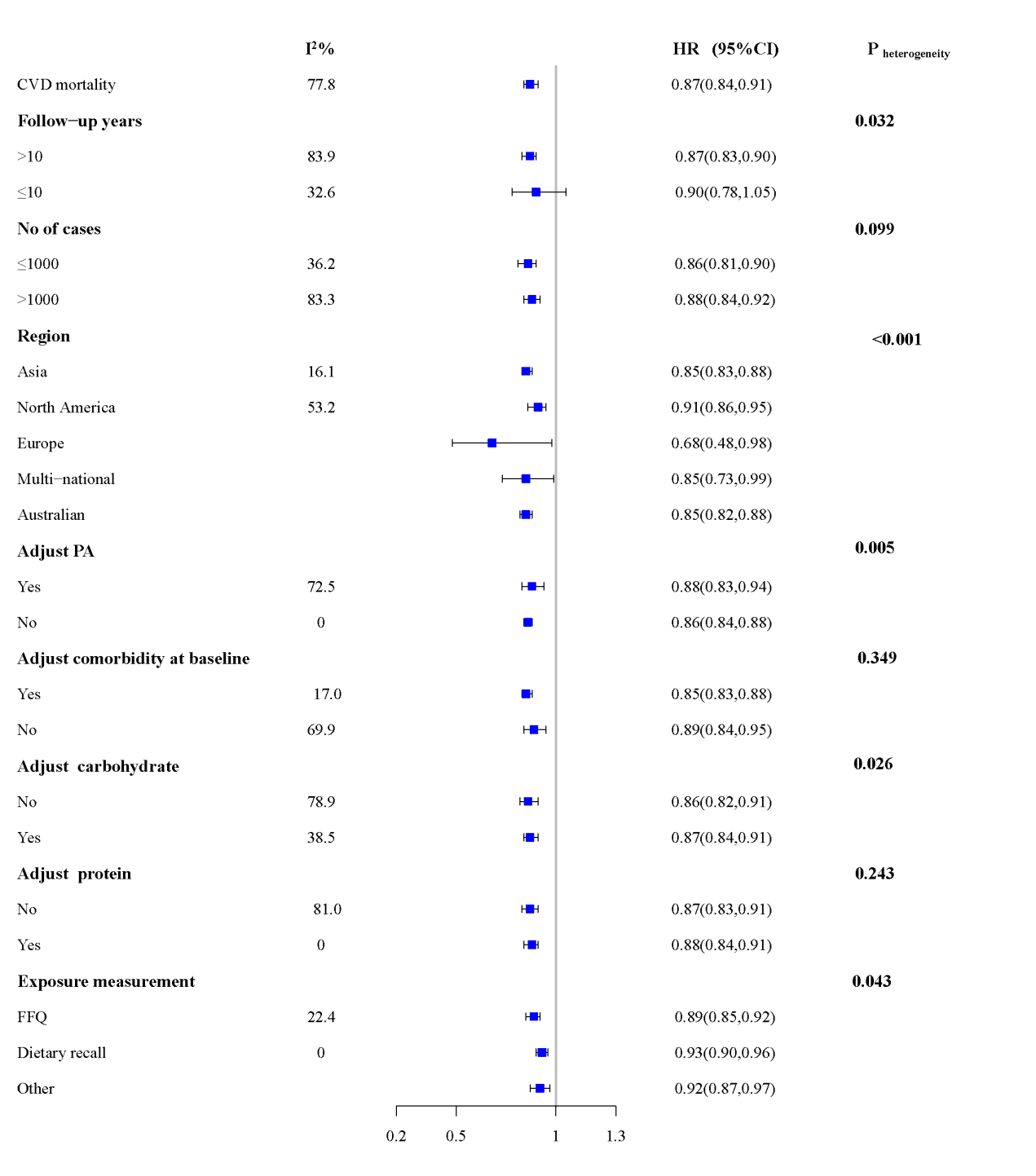


A

B

C

**Supplemental Figure 7.** Subgroup analysis of dietary fiber intake and risk of all cause mortality (A) , CVD mortality (B) and cancer mortality (C), per 10 g fiber intake.

**Supplemental Table 9.** Sensitivity analysis of dietary fiber intake and all cause mortality, CVD mortality and cancer mortality, per 10 g intake.

| **Study omitted** | | **RR** | | | **95% CI** | | | |
| --- | --- | --- | --- | --- | --- | --- | --- | --- |
| **All cause mortality** | |  | | |  |  | | |
| Zhang(2022) | | 0.89 | 0.86 | | | 0.93 |  |  |
| You(2022) | | 0.90 | 0.87 | | | 0.94 |  |  |
| Xu(2022) | | 0.89 | 0.85 | | | 0.94 |  |  |
| Kwon(2022) | | 0.90 | 0.87 | | | 0.94 |  |  |
| Ha(2021) | | 0.89 | 0.86 | | | 0.92 |  |  |
| Partula(2020) | | 0.89 | 0.86 | | | 0.93 |  |  |
| Ho(2020) | | 0.91 | 0.87 | | | 0.94 |  |  |
| Katagiri(2020) | | 0.91 | 0.87 | | | 0.94 |  |  |
| Dominguez(2019) | | 0.9 | 0.87 | | | 0.94 |  |  |
| Xu(2016) | | 0.89 | 0.86 | | | 0.94 |  |  |
| Xu(2014) | | 0.90 | 0.87 | | | 0.93 |  |  |
| Cosiales(2014) | | 0.90 | 0.87 | | | 0.94 |  |  |
| Chuang(2012) | | 0.89 | 0.85 | | | 0.93 |  |  |
| Streppel(2008) | | 0.90 | 0.86 | | | 0.93 |  |  |
|  | |  | | |  |  | | |
| **CVD mortality** | |  | | |  |  | | |
| Kwon(2022) | | 0.87 | 0.84 | | | 0.91 |  |  |
| Xu(2022) | | 0.87 | 0.83 | | | 0.91 |  |  |
| Ha(2021) | | 0.86 | 0.83 | | | 0.90 |  |  |
| Katagiri(2020) | | 0.87 | 0.83 | | | 0.91 |  |  |
| Miyazawa(2020) | | 0.87 | 0.84 | | | 0.91 |  |  |
| Xu(2016) | | 0.86 | 0.84 | | | 0.88 |  |  |
| Xu(2014) | | 0.87 | 0.83 | | | 0.90 |  |  |
| Cosiales(2014) | | 0.87 | 0.84 | | | 0.91 |  |  |
| Crowe(2012) | | 0.87 | 0.84 | | | 0.91 |  |  |
| Buyken(2010) | | 0.87 | 0.84 | | | 0.91 |  |  |
| Eshak(2010) | | 0.87 | 0.84 | | | 0.91 |  |  |
| Liu(2002) | | 0.87 | 0.84 | | | 0.91 |  |  |
| KHAW(1987) | | 0.87 | 0.84 | | | 0.91 |  |  |
|  | |  | | |  |  | | |
| **Cancer mortality** | |  | | |  |  | | |
| Xu(2022) | | 0.90 | 0.87 | | | 0.94 |  |  |
| Ha(2021) | | 0.91 | 0.89 | | | 0.93 |  |  |
| Katagiri(2020) | | 0.91 | 0.88 | | | 0.94 |  |  |
| Xu(2016) | | 0.90 | 0.87 | | | 0.92 |  |  |
| Cosiales(2014) | | 0.91 | 0.88 | | | 0.93 |  |  |
| Xu(2014) | | 0.91 | 0.88 | | | 0.93 |  |  |
| Chuang(2012) | | 0.90 | 0.87 | | | 0.93 |  |  |

A


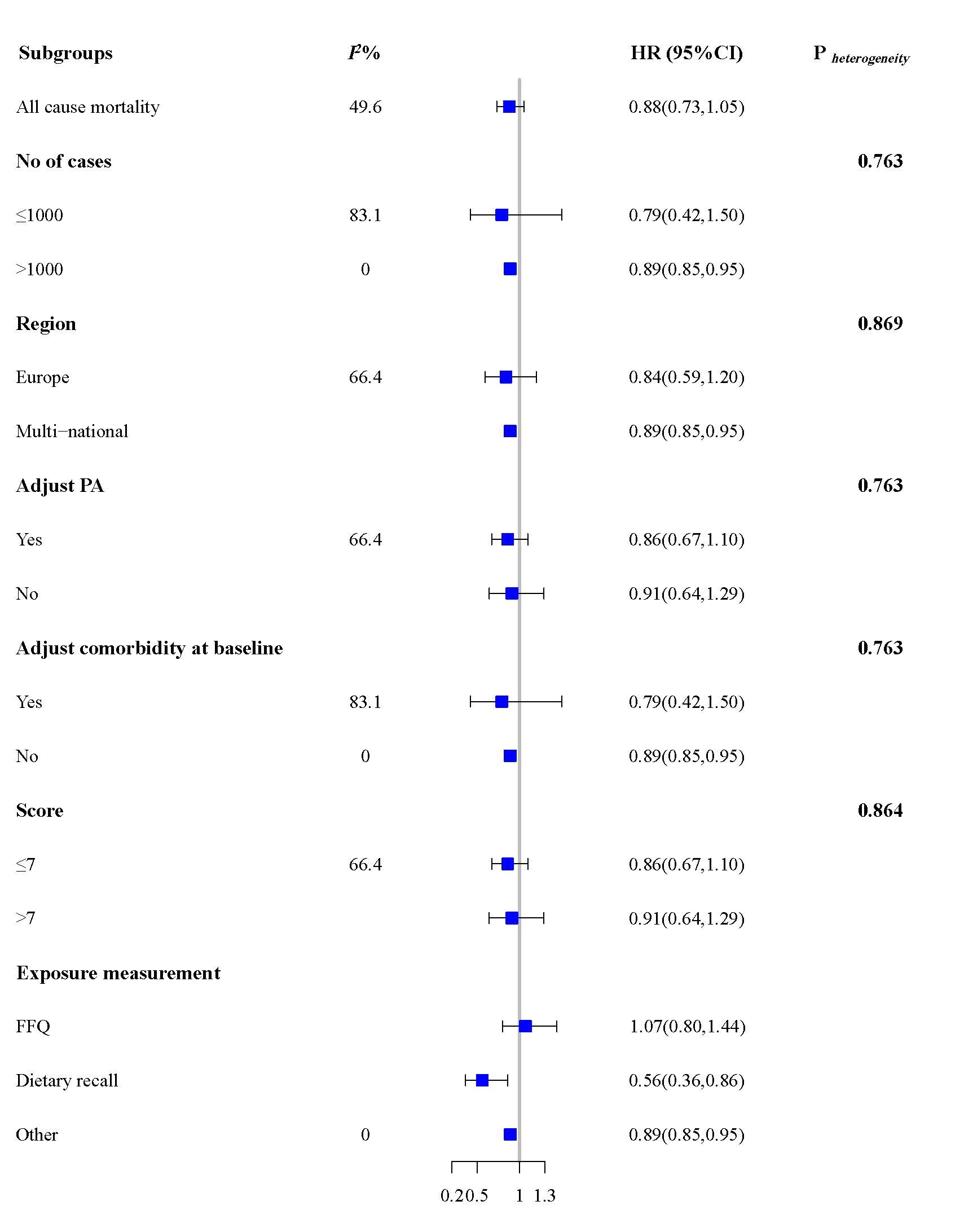

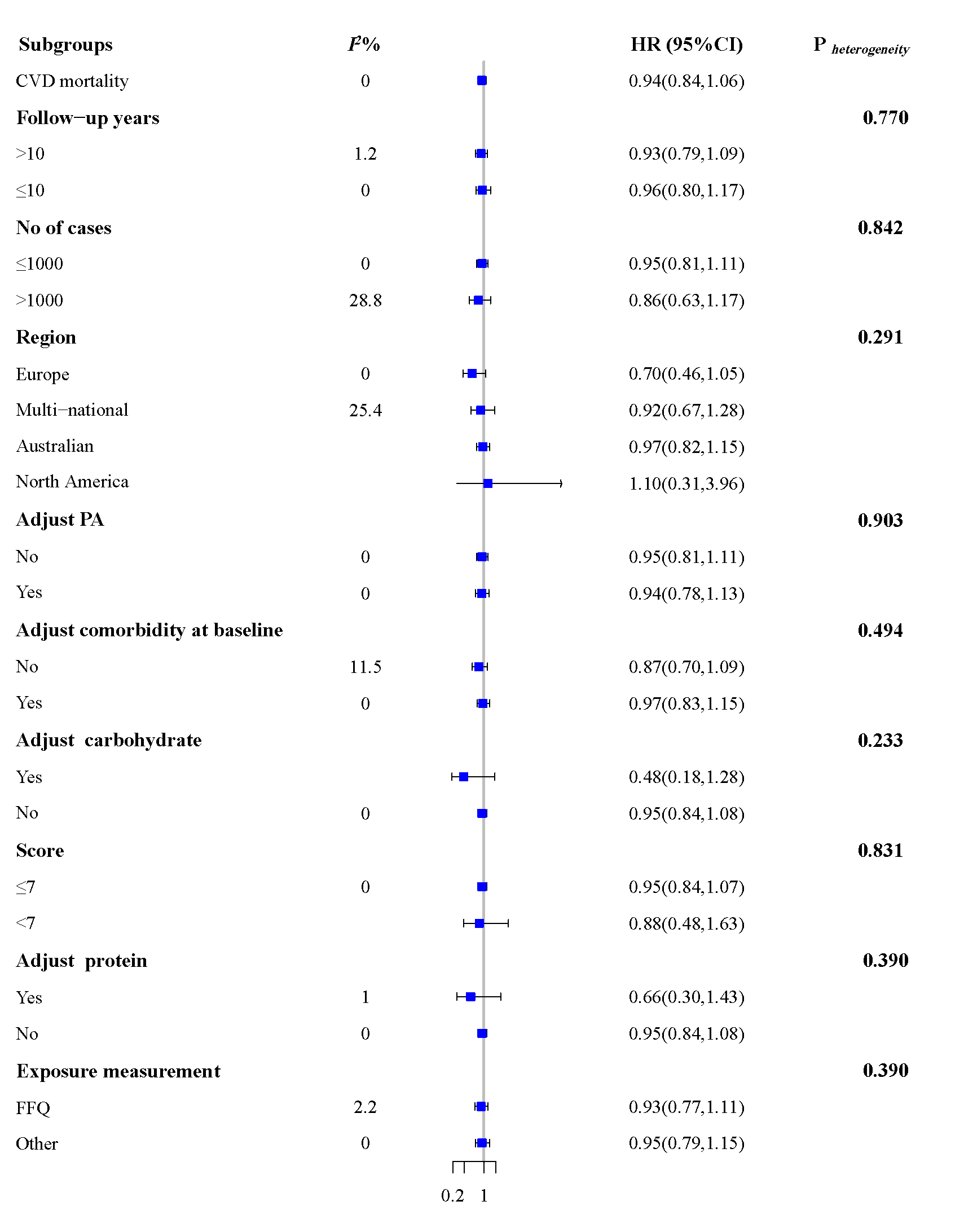


**Supplemental Figure 8**. Subgroup analysis of vegetable fiber intake and risk of all cause mortality(A) and CVD mortality(B), per 10 g fiber intake.

B

**Supplemental Table 10**. Sensitivity analysis of vegetable fiber intake and all cause mortality, CVD mortality and cancer mortality, per 10 g intake.

| **Study omitted** | **RR** | **95% CI** | |
| --- | --- | --- | --- |
| **All-cause mortality** |  |  |  |
| Partula(2020) | 1.07 | 0.80 | 1.45 |
| Dominguez(2019) | 0.56 | 0.37 | 0.87 |
| Chuang(2012) | 0.89 | 0.85 | 0.95 |
| Streppel(2008)men | 0.91 | 0.64 | 1.29 |
|  |  |  |  |
| **CVD mortality** |  |  |  |
| Threapleton(2012)women | 0.95 | 0.84 | 1.08 |
| Crowe(2012) | 0.95 | 0.84 | 1.08 |
| Buyken(2010) | 0.91 | 0.77 | 1.09 |
| Streppel(2008)men | 0.95 | 0.84 | 1.07 |
| Liu(2002)women | 0.94 | 0.83 | 1.06 |
| Pietinen(1996) | 0.96 | 0.85 | 1.08 |
|  |  |  |  |

A


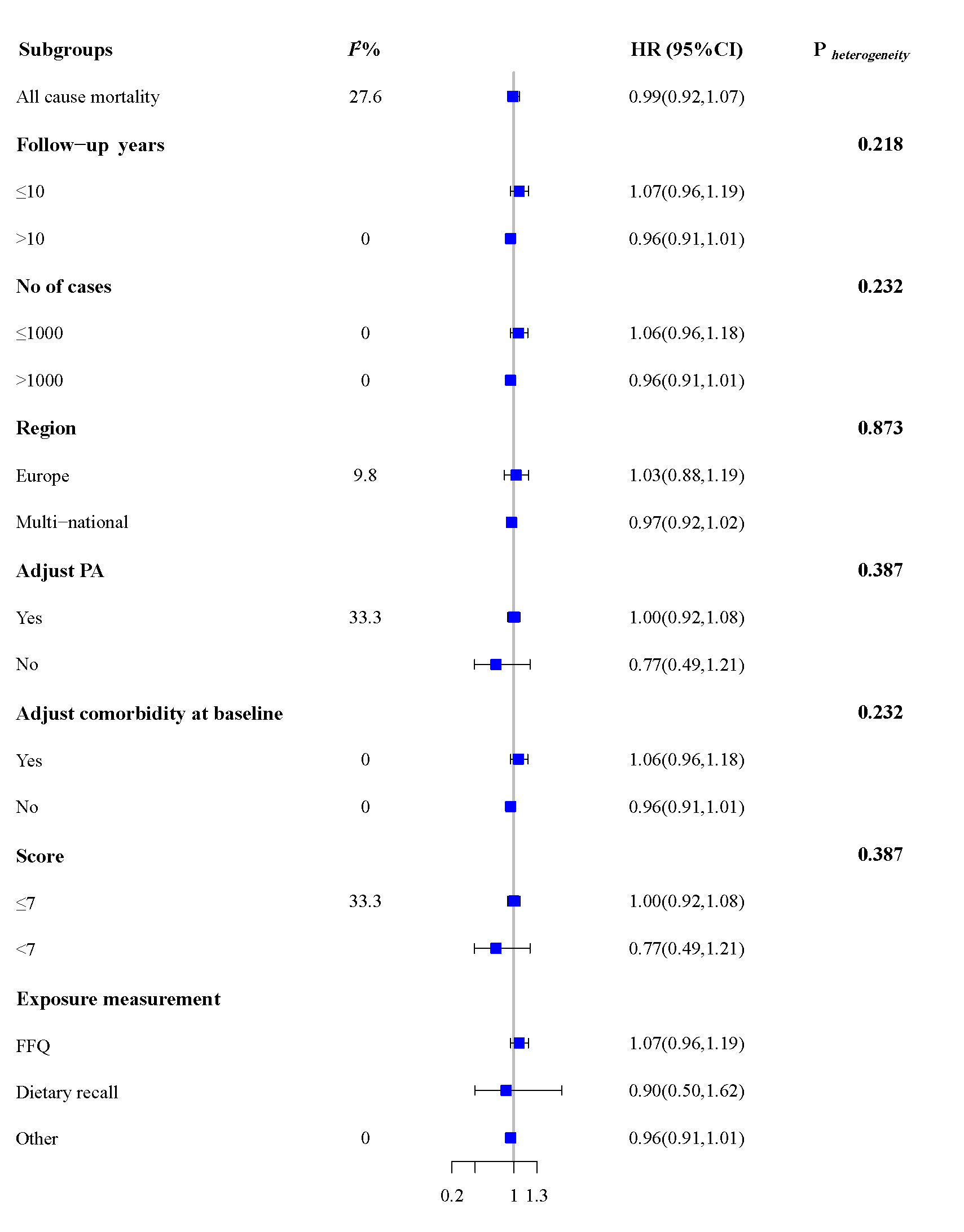

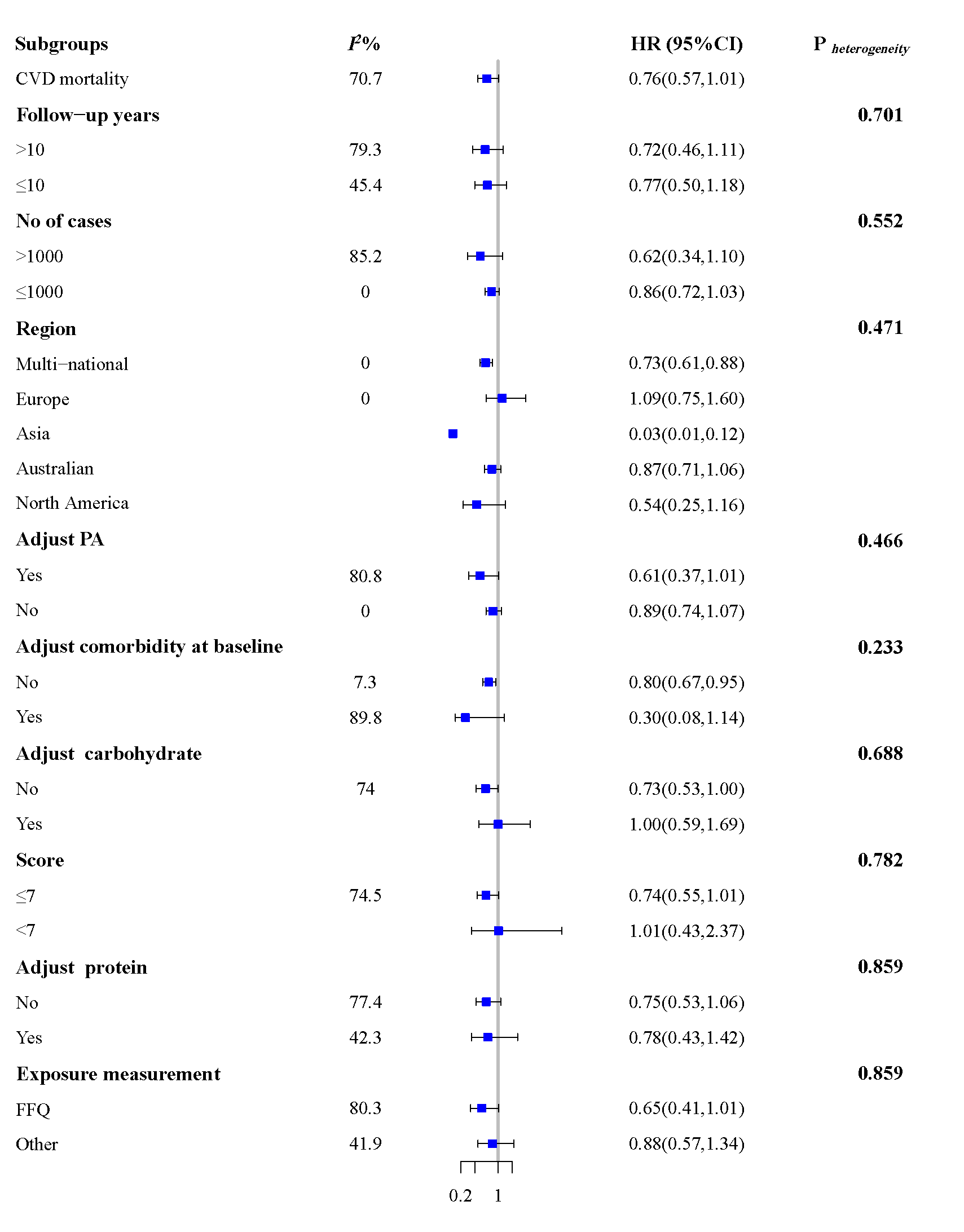


**Supplemental Figure 9.** Subgroup analysis of fruit fiber intake and risk of all cause mortality(A) and CVD mortality(B), per 10 g fiber intake.

B

**Supplemental Table 11**. Sensitivity analysis of fruit fiber intake and all cause mortality, CVD mortality and cancer mortality, per 10 g intake.

| **Study omitted** | **RR** | **95% CI** | |
| --- | --- | --- | --- |
| **All-cause mortality** |  |  |  |
| Partula(2020) | 0.96 | 0.91 | 1.01 |
| Dominguez(2019) | 0.99 | 0.90 | 1.10 |
| Chuang(2012) | 1.02 | 0.88 | 1.19 |
| Streppel(2008)men | 1.00 | 0.92 | 1.08 |
| Streppel (2008) men | 0.96 | 0.91 | 1.01 |
|  |  |  |  |
| **CVD mortality** |  |  |  |
| Crowe(2012) | 0.74 | 0.51 | 1.07 |
| Threapleton(2012)women | 0.73 | 0.53 | 1.00 |
| Eshak(2010) | 0.81 | 0.71 | 0.92 |
| Buyken(2010) | 0.72 | 0.49 | 1.05 |
| Streppel(2008)men | 0.74 | 0.55 | 1.01 |
| Liu(2002)women | 0.79 | 0.58 | 1.07 |
| Pietinen(1996)men | 0.72 | 0.53 | 0.97 |

A


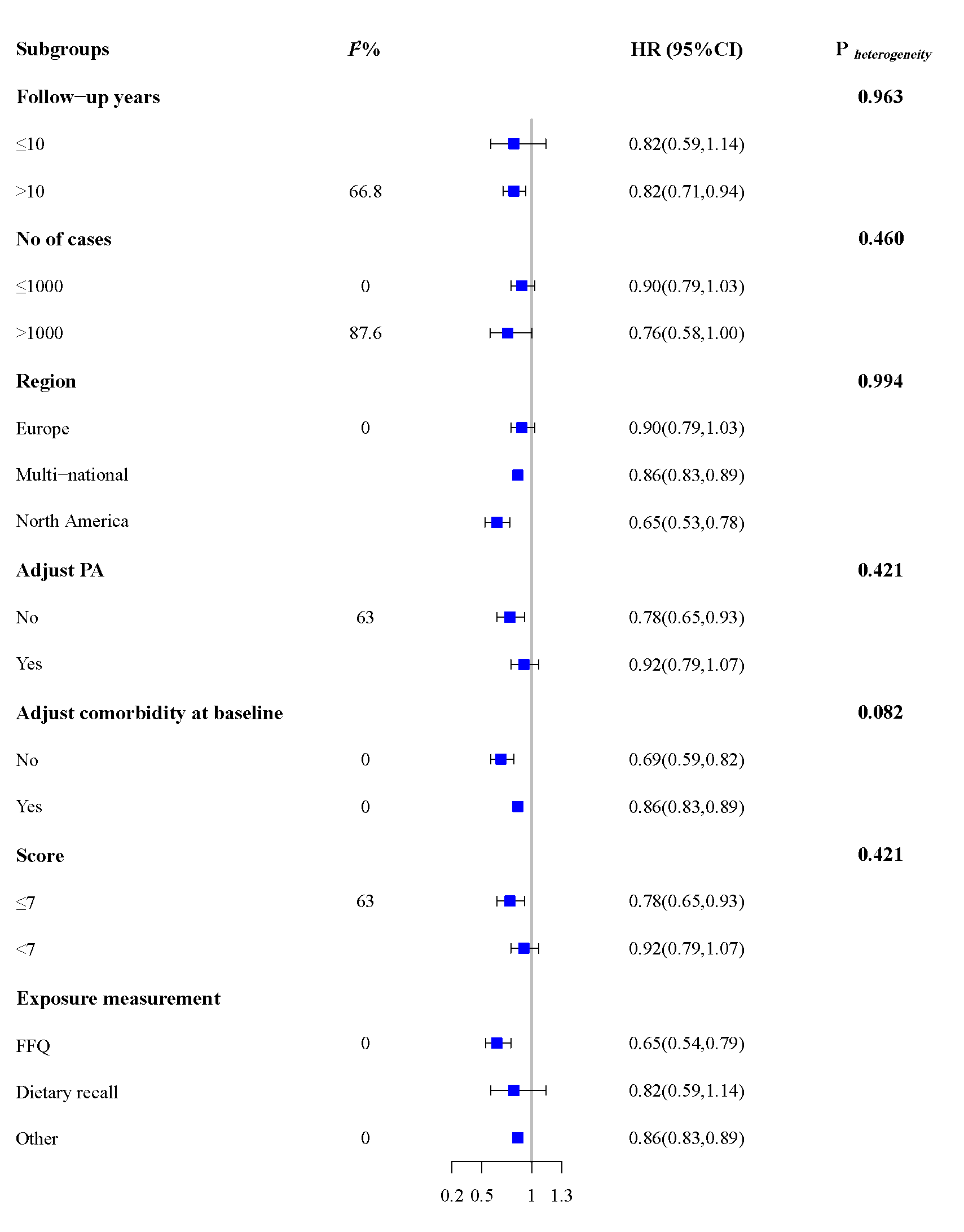

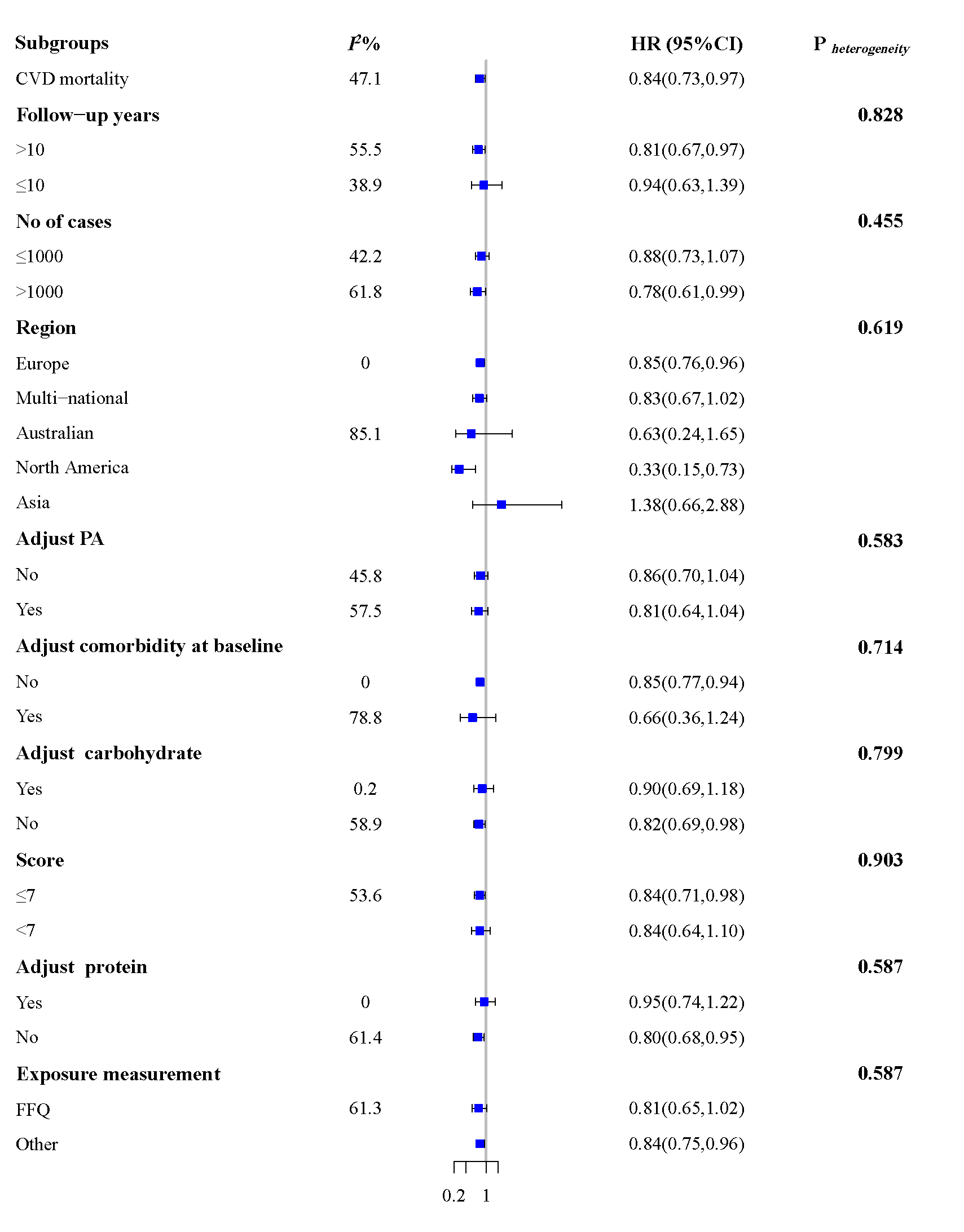


**Supplemental Figure 10.** Subgroup analysis of cereal fiber intake and risk of all-cause mortality(A) and CVD mortality(B), per 10 g fiber intake.

B

**Supplemental Table 12**. Sensitivity analysis of cereal fiber intake and CVD mortality, per 10 g intake.

| **Study omitted** | **RR** | **95% CI** | |
| --- | --- | --- | --- |
| **All-cause mortality** |  |  |  |
| Partula(2020) | 0.82 | 0.71 | 0.94 |
| Dominguez(2019) | 0.82 | 0.72 | 0.93 |
| Chuang(2012) | 0.79 | 0.64 | 0.98 |
| Baer(2010)women | 0.86 | 0.83 | 0.89 |
| Streppel(2008)men | 0.78 | 0.65 | 0.93 |
| **CVD mortality** |  |  |  |
| Threapleton(2012)women | 0.82 | 0.71 | 0.96 |
| Crowe(2012) | 0.84 | 0.71 | 0.99 |
| Threapleton(2012) | 0.84 | 0.72 | 0.98 |
| Buyken(2010) | 0.81 | 0.69 | 0.95 |
| Eshak(2010) | 0.87 | 0.78 | 0.97 |
| Kaushik(2009) | 0.87 | 0.78 | 0.97 |
| Streppel(2008)men | 0.84 | 0.71 | 0.98 |
| Liu(2002)women | 0.83 | 0.72 | 0.95 |
| Pietinen(1996)men | 0.83 | 0.69 | 0.99 |

**
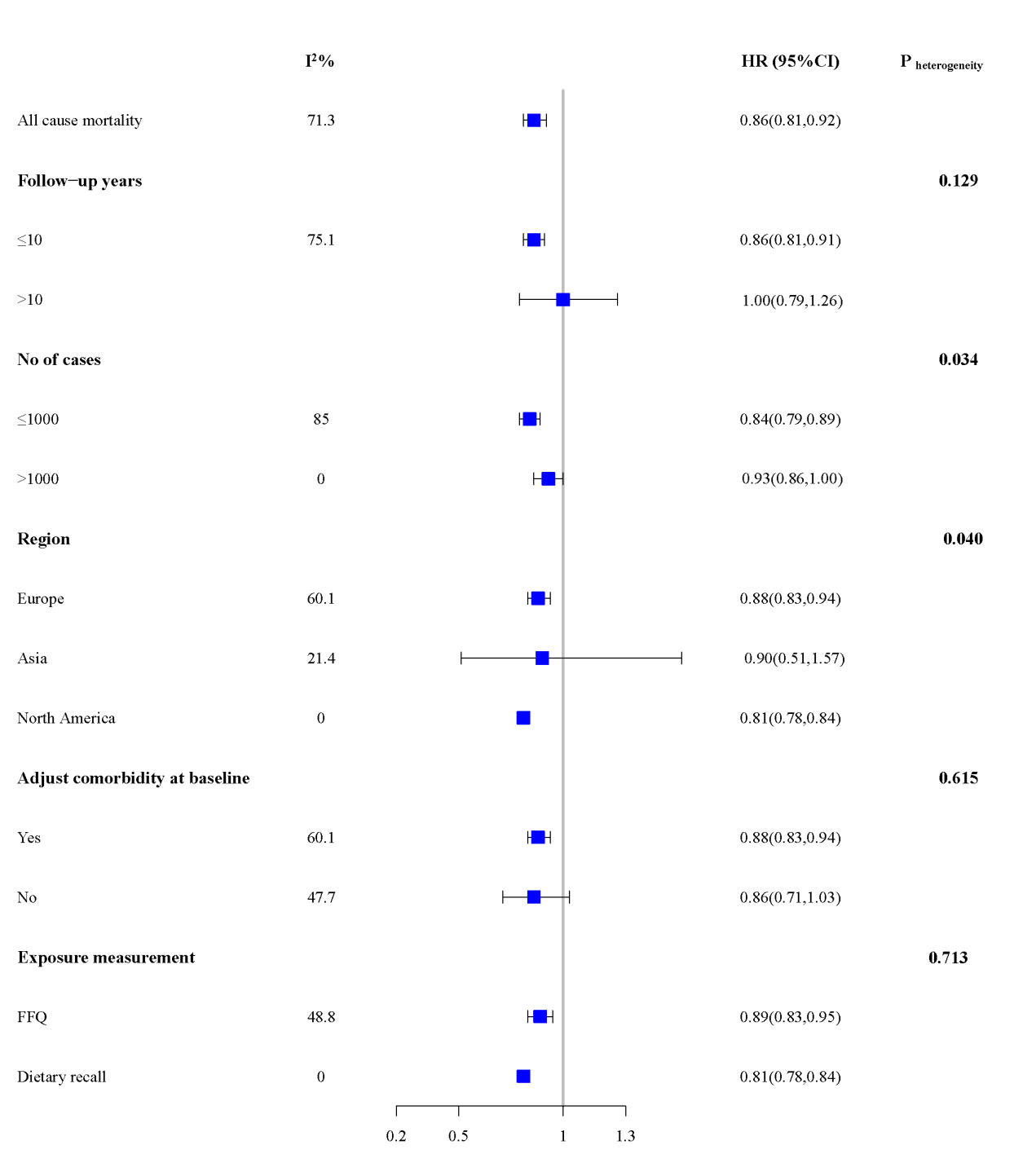
**
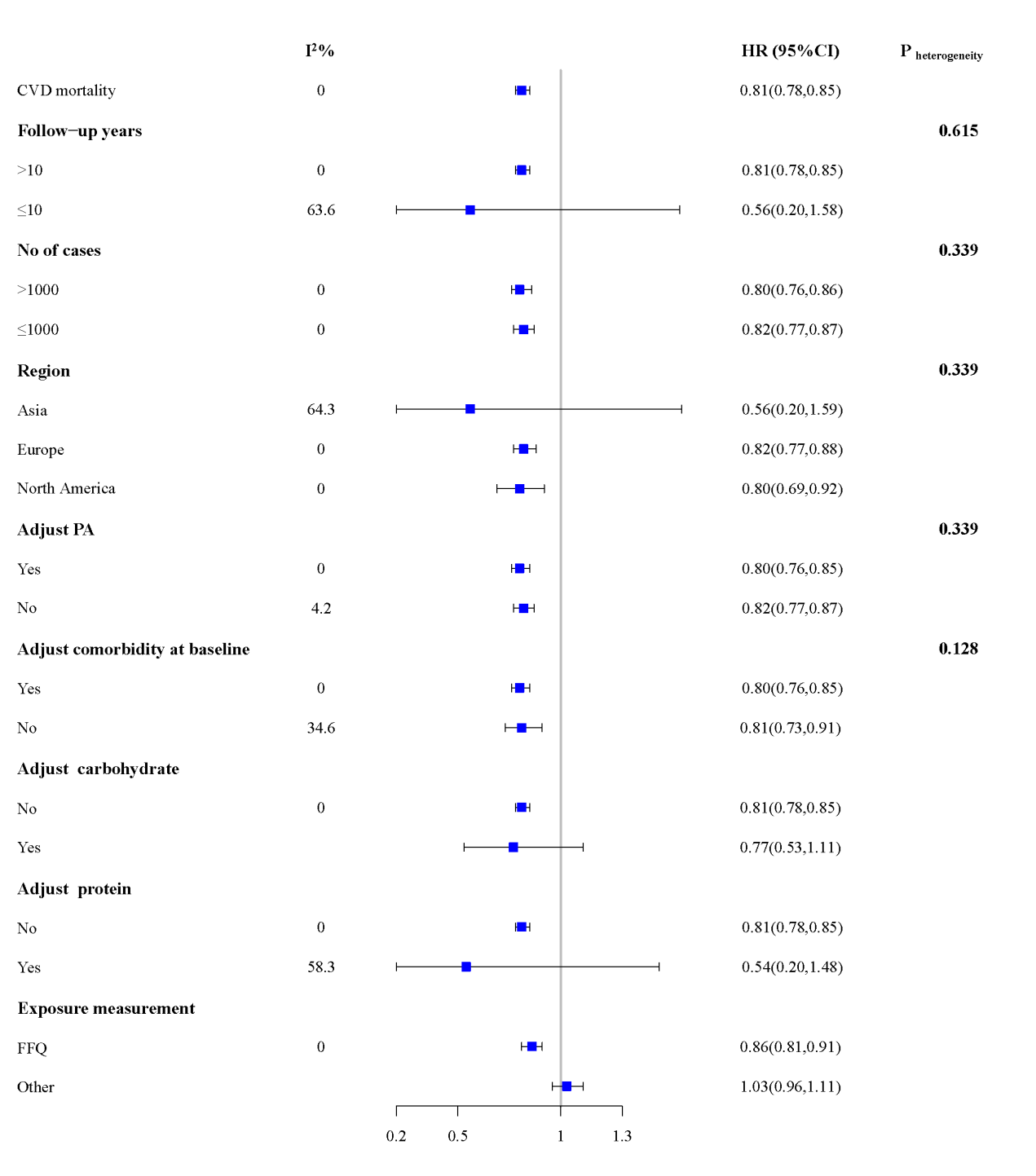


A

**Supplemental Figure 11.** Subgroup analysis of insoluble fiber intake and risk of all cause mortality (A) and CVD mortality (B), per 10 g fiber intake.

B

**Supplemental Table 13**. Sensitivity analysis of insoluble fiber intake and all cause mortality, CVD mortality and cancer mortality, per 10 g intake.

| **Study omitted** | **RR** | **95% CI** | |
| --- | --- | --- | --- |
| **All-cause mortality** |  |  |  |
| Xu(2022) | 0.88 | 0.78 | 0.99 |
| Partula(2020) | 0.85 | 0.81 | 0.91 |
| Katagiri(2020) | 0.89 | 0.83 | 0.95 |
| Dominguez(2019) | 0.86 | 0.81 | 0.92 |
| Chan(2016) | 0.84 | 0.79 | 0.90 |
|  |  |  |  |
| **CVD mortality** |  |  |  |
| Xu(2022) | 0.82 | 0.77 | 0.87 |
| Katagiri(2020) | 0.80 | 0.76 | 0.85 |
| Threapleton(2012)women | 0.81 | 0.78 | 0.85 |
| Eshak(2010) | 0.81 | 0.78 | 0.85 |
| Liu(2002) | 0.81 | 0.78 | 0.85 |
| Pietinen(1996) | 0.81 | 0.78 | 0.85 |
|  |  |  |  |
| **Ccancer mortality** |  |  |  |
| Xu(2022) | 0.99 | 0.86 | 1.12 |
| Katagiri(2020) | 0.86 | 0.81 | 0.91 |
| Chan(2016) | 0.94 | 0.78 | 1.13 |

**
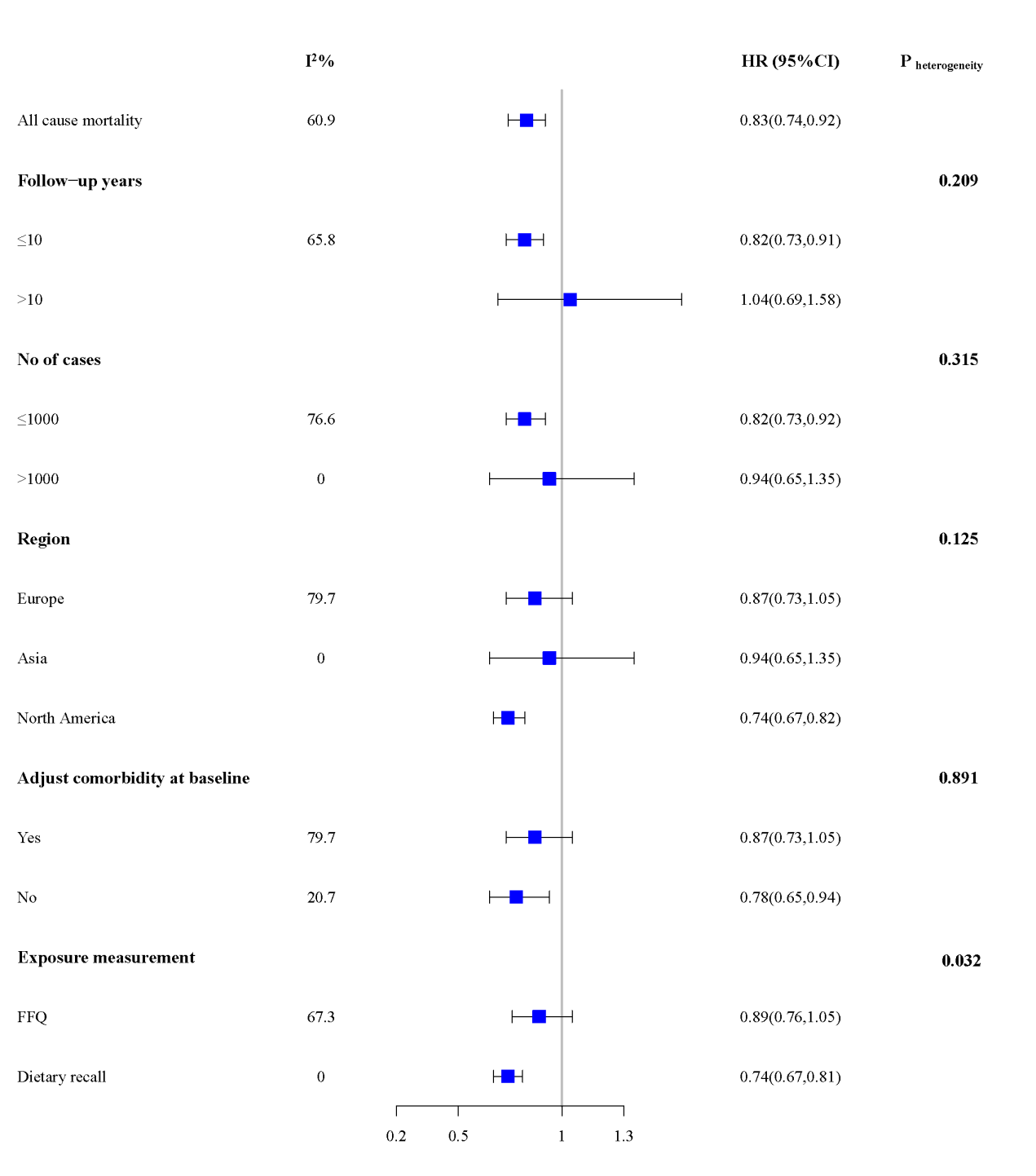
**
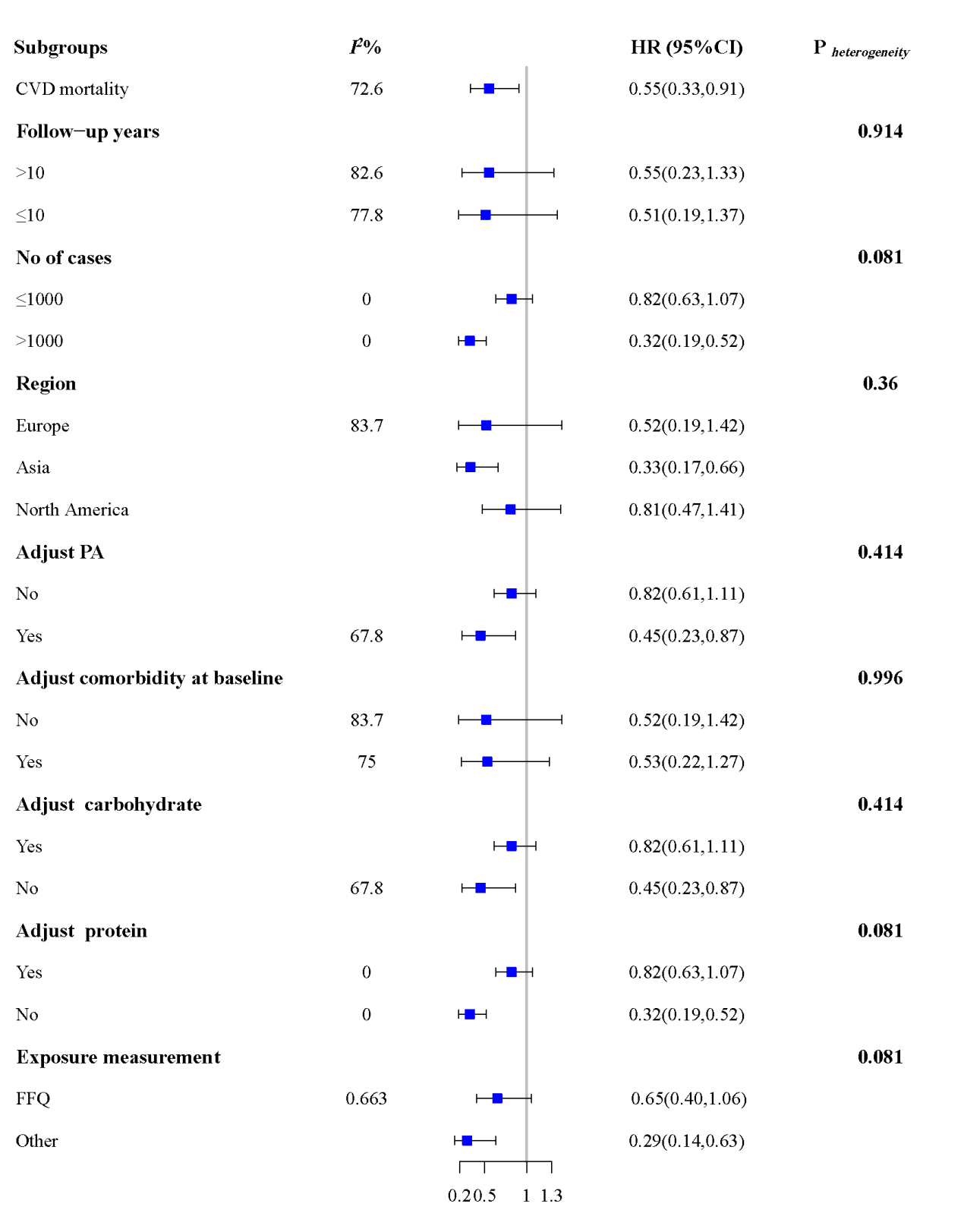


A

**Supplemental Figure 12.** Subgroup analysis of soluble fiber intake and risk of all cause mortality (A) and CVD mortality (B), per 10 g fiber intake.

B

B

**Supplemental Table 14**. Sensitivity analysis of soluble fiber intake and all cause mortality, CVD mortality, per 10 g intake.

| **Study omitted** | **RR** | **95% CI** | |
| --- | --- | --- | --- |
| **All-cause mortality** |  |  |  |
| Xu(2022) | 0.86 | 0.69 | 1.06 |
| Partula(2020) | 0.82 | 0.73 | 0.91 |
| Katagiri(2020) | 0.88 | 0.76 | 1.02 |
| Dominguez(2019) | 0.83 | 0.74 | 0.93 |
| Chan(2016) | 0.79 | 0.73 | 0.85 |
|  |  |  |  |
| **CVD mortality** |  |  |  |
| Xu(2022) | 0.55 | 0.33 | 0.91 |
| Threapleton(2012)women | 0.54 | 0.35 | 0.83 |
| Eshak(2010) | 0.70 | 0.54 | 0.90 |
| Liu(2002)women | 0.58 | 0.41 | 0.82 |
| Pietinen(1996)men | 0.70 | 0.55 | 0.88 |

Supplemental Figures


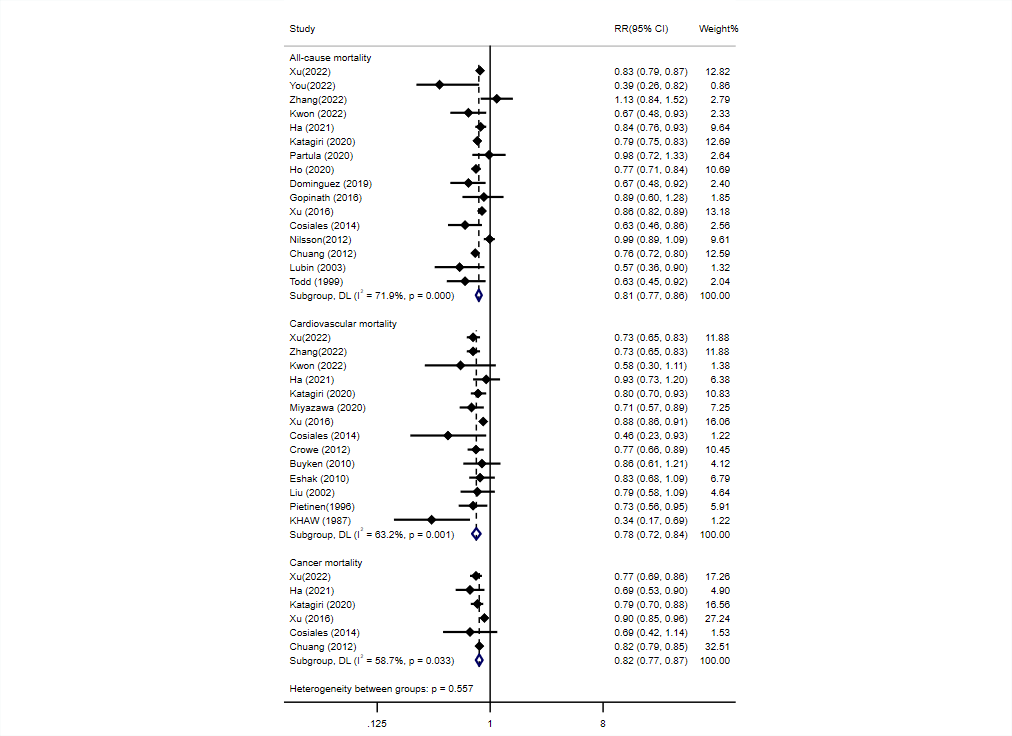


Supplemental Figure 13. Forest plots for highest vs. lowest intake meta-analysis of the association between dietary fiber intake and risk of all-cause, CVD and cancer mortality. RR, relative risk.

**
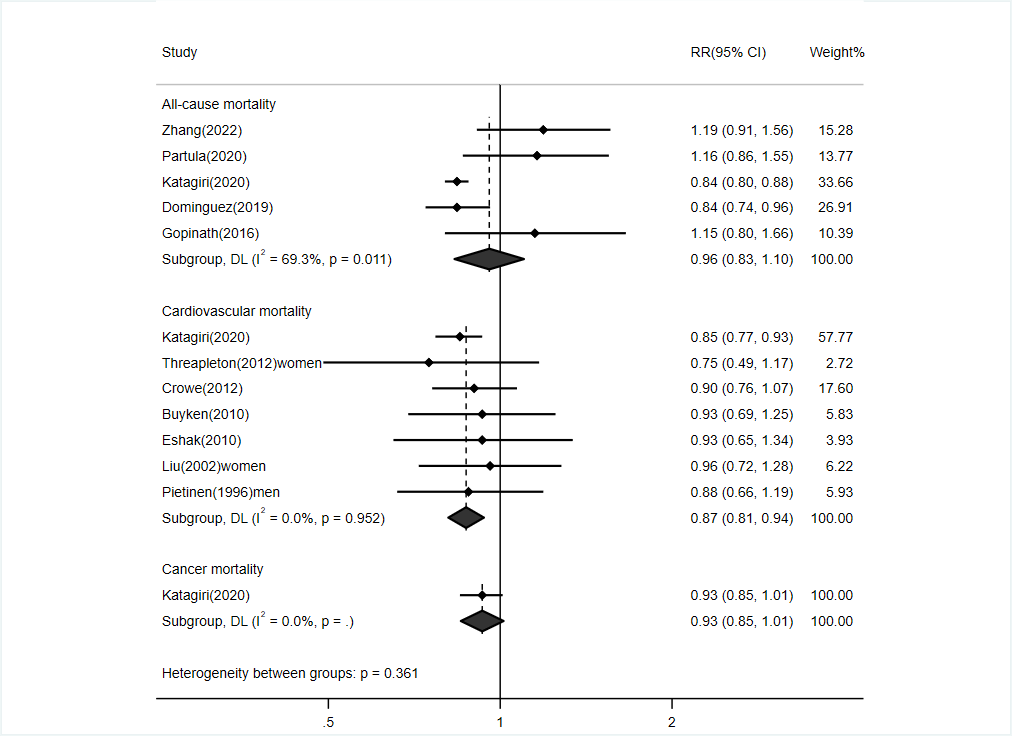
**

Supplemental Figure 14. Forest plots for highest vs. lowest intake meta-analysis of the association between vegetable fiber intake and risk of all-cause and CVD mortality. RR, relative risk.


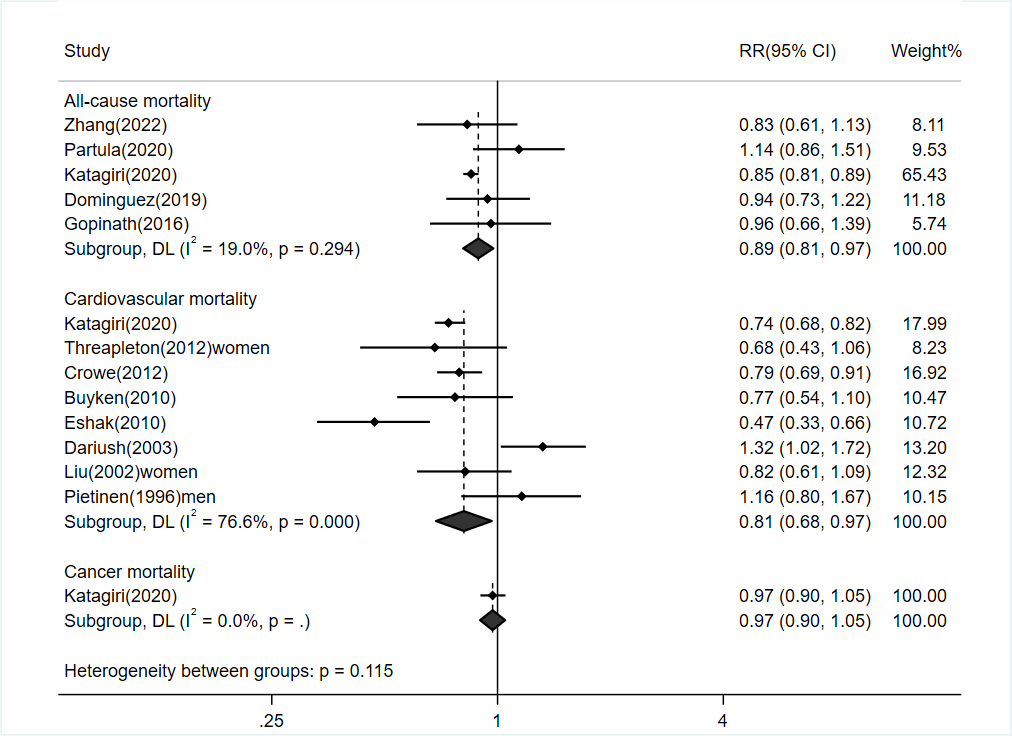


Supplemental Figure 15. Forest plots for highest vs. lowest intake meta-analysis of the association between fruit fiber intake and risk of all-cause, CVD and cancer mortality. RR, relative risk.


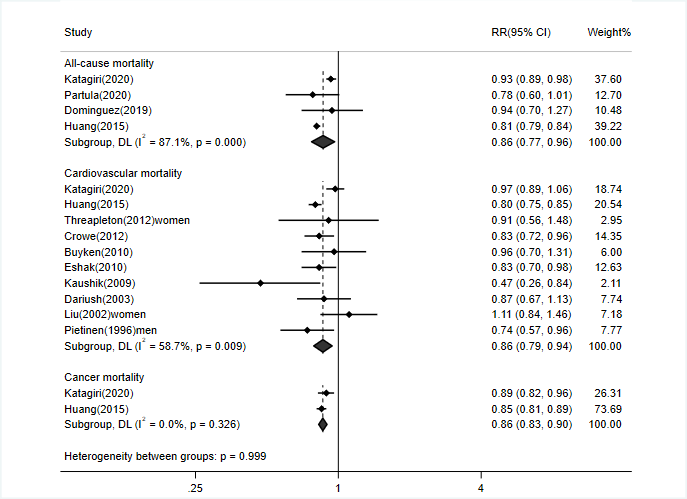


Supplemental Figure 16. Forest plots for highest vs. lowest intake meta-analysis of the association between cereal fiber intake and risk of all-cause, CVD and cancer mortality. RR, relative risk.


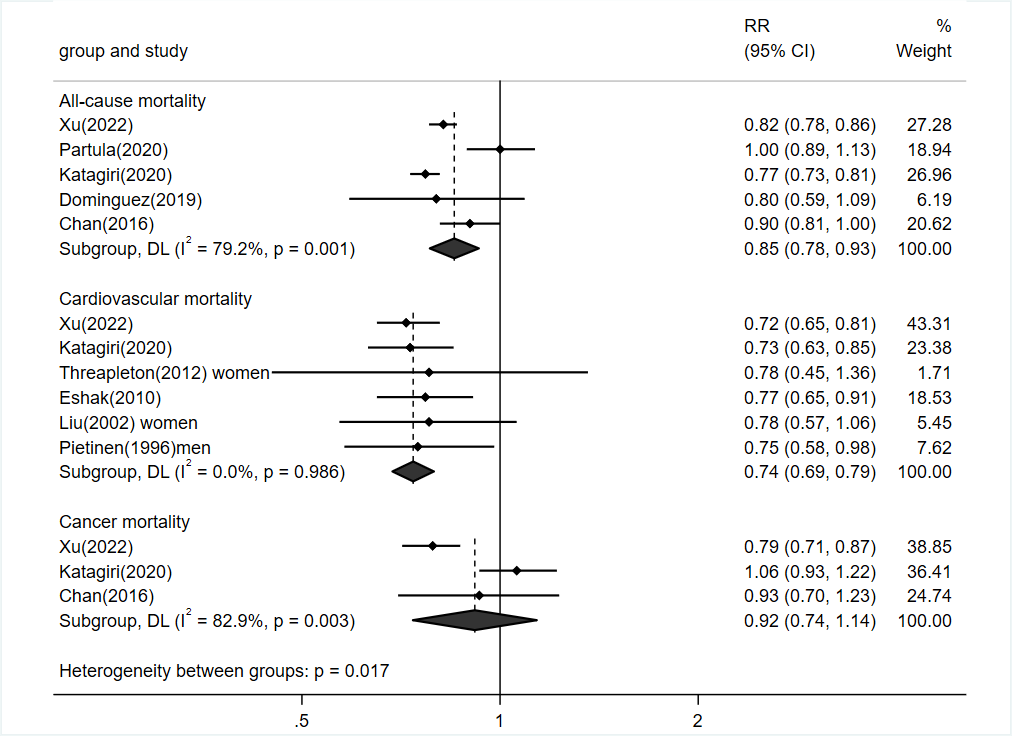


Supplemental Figure 17. Forest plots for highest vs. lowest intake meta-analysis of the association between insoluble fiber intake and risk of all-cause, CVD and cancer mortality. RR, relative risk.


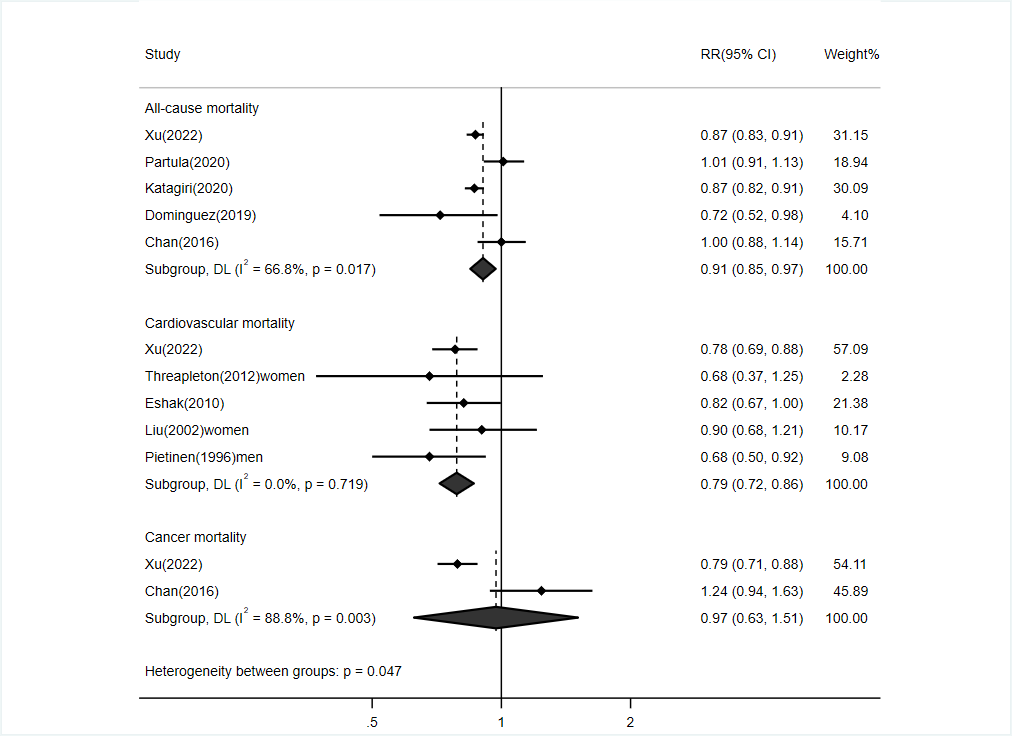


Supplemental Figure 18. Forest plots for highest vs. lowest intake meta-analysis of the association between soluble fiber intake and risk of all-cause and CVD mortality. RR, relative risk.


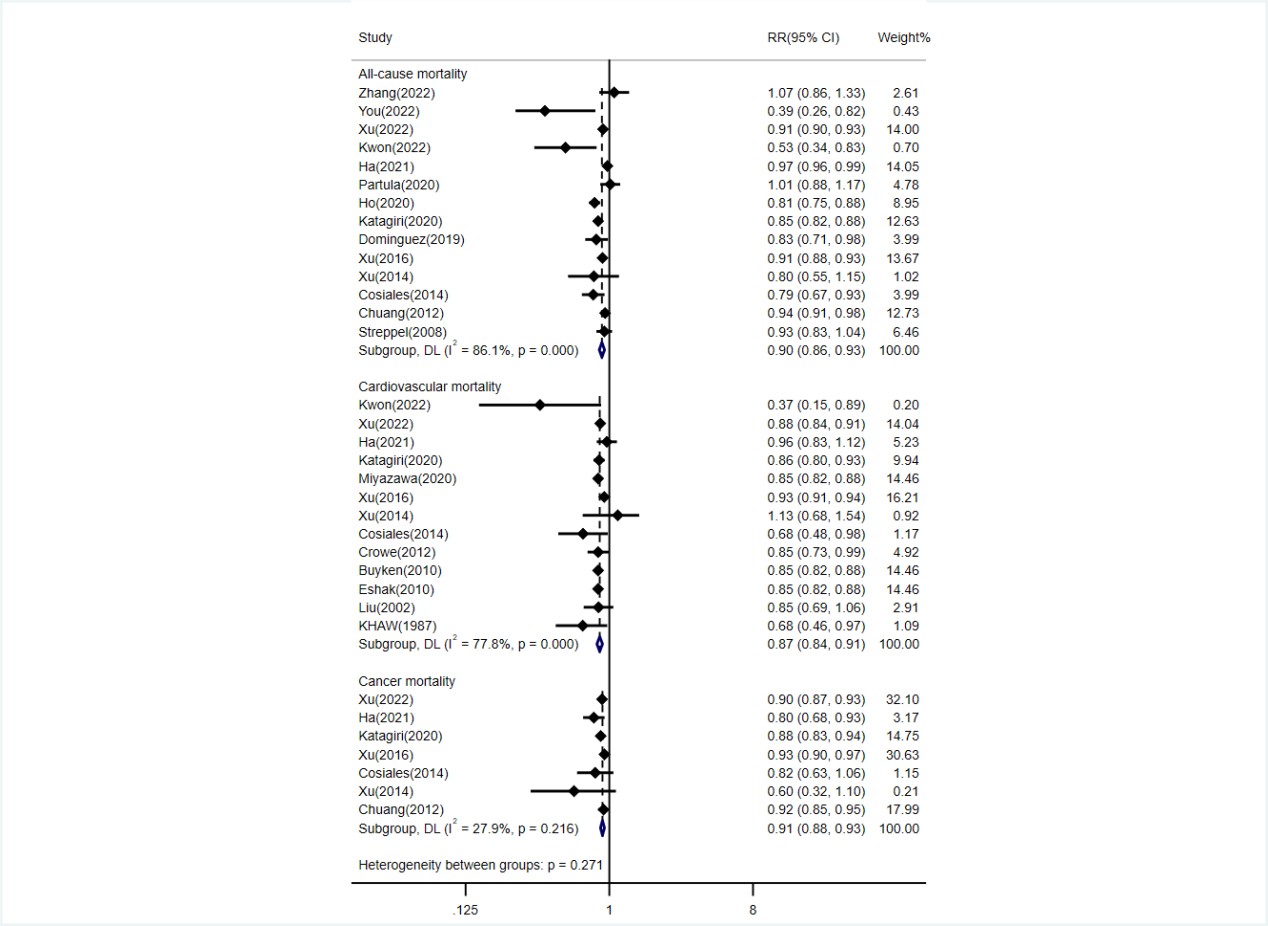


Supplemental Figure 19. Forest plots of the association between dietary fiber intake and risk of all-cause, CVD and cancer mortality. RR, relative risk, per 10 g intake.


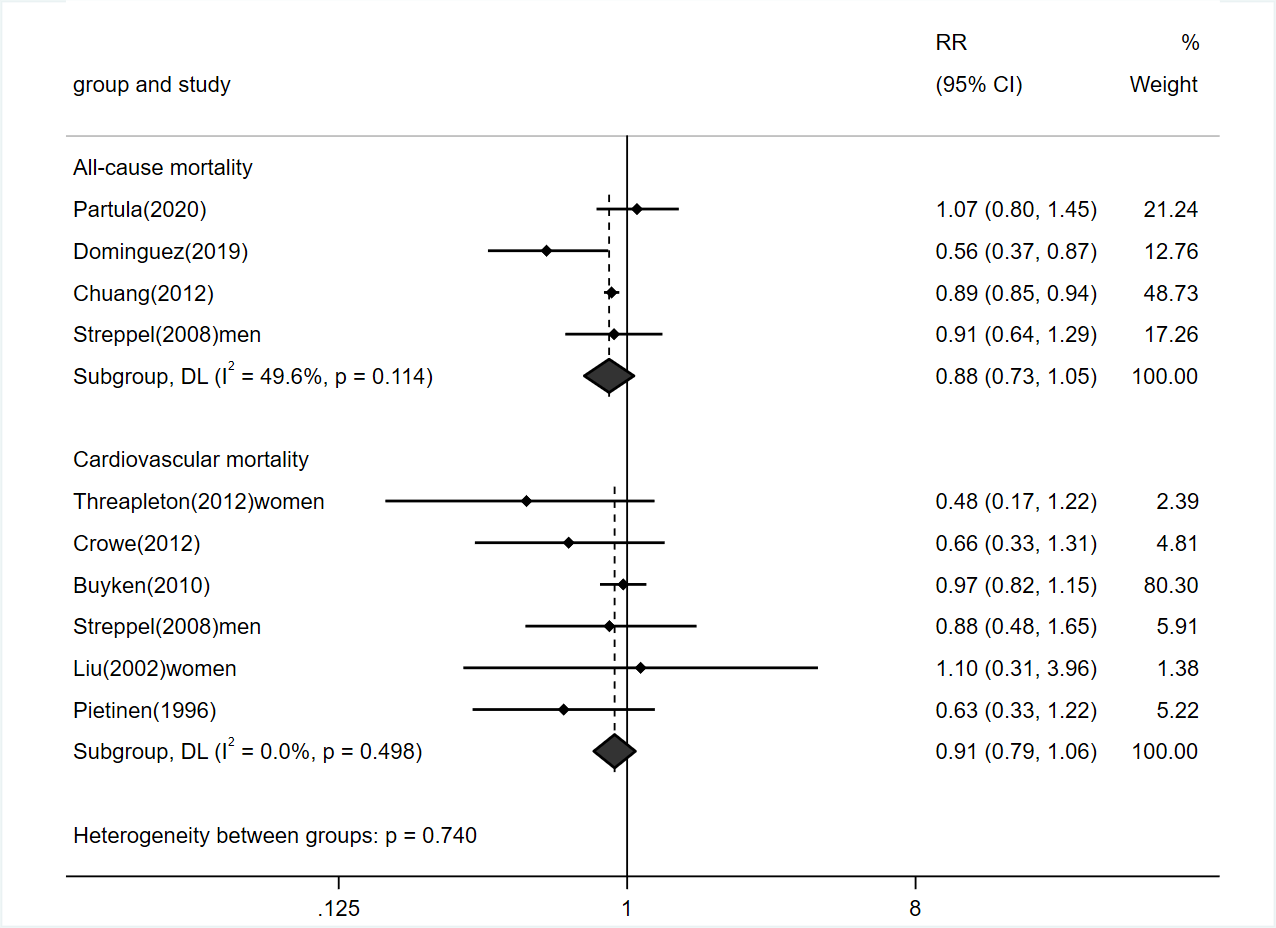


Supplemental Figure 20. Forest plots of the association between vegetable fiber intake and risk of all-cause, CVD and cancer mortality. RR, relative risk, per 10 g intake.


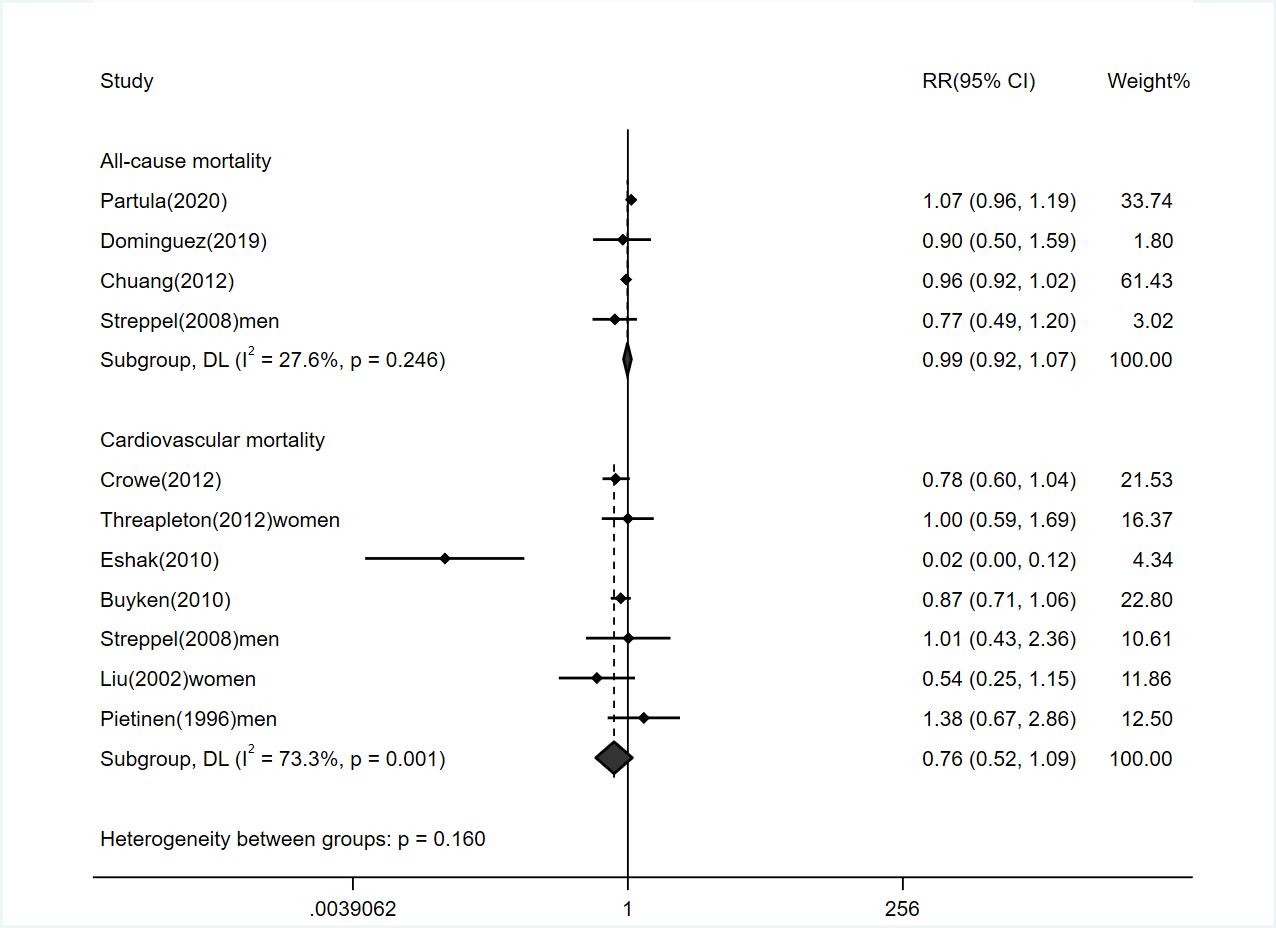


Supplemental Figure 21. Forest plots of the association between fruit fiber intake and risk of all-cause, CVD and cancer mortality. RR, relative risk, per 10 g intake.


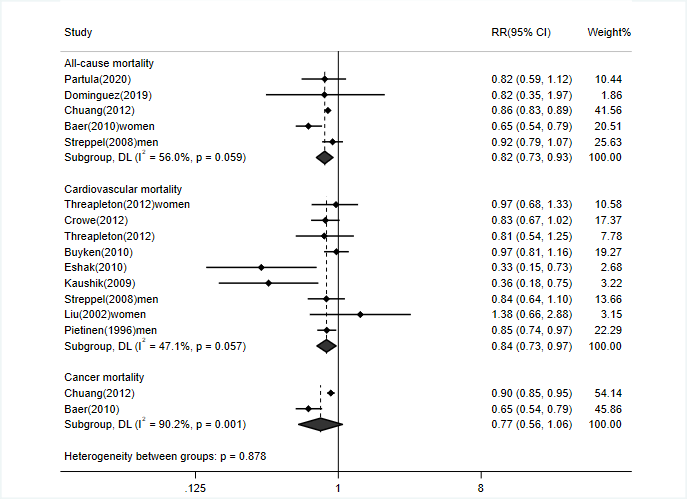


Supplemental Figure 22. Forest plots of the association between cereal fiber intake and risk of all-cause, CVD and cancer mortality. RR, relative risk, per 10 g intake.


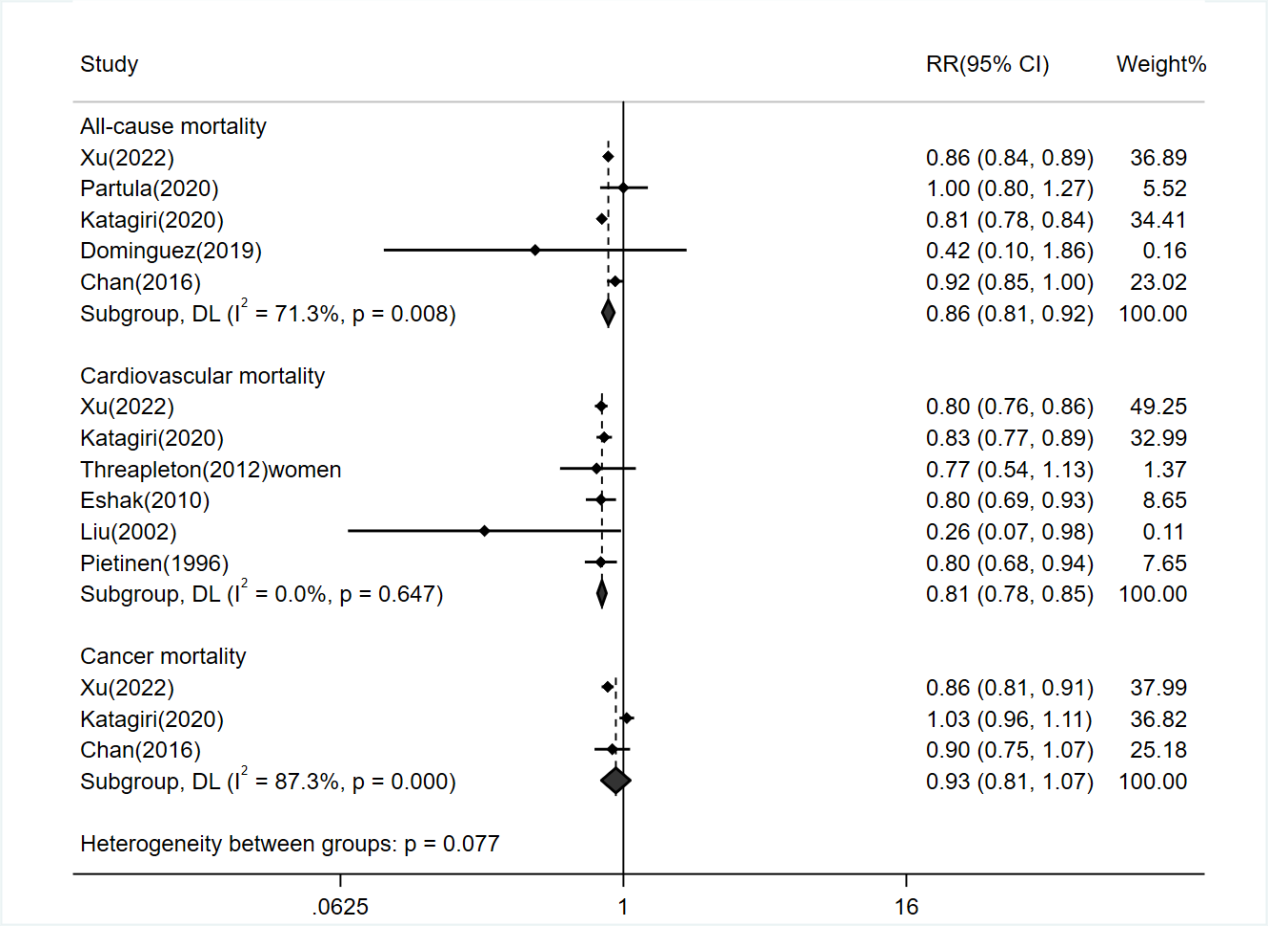


Supplemental Figure 23. Forest plots of the association between insoluble fiber intake and risk of all-cause, CVD and cancer mortality. RR, relative risk, per 10 g intake.


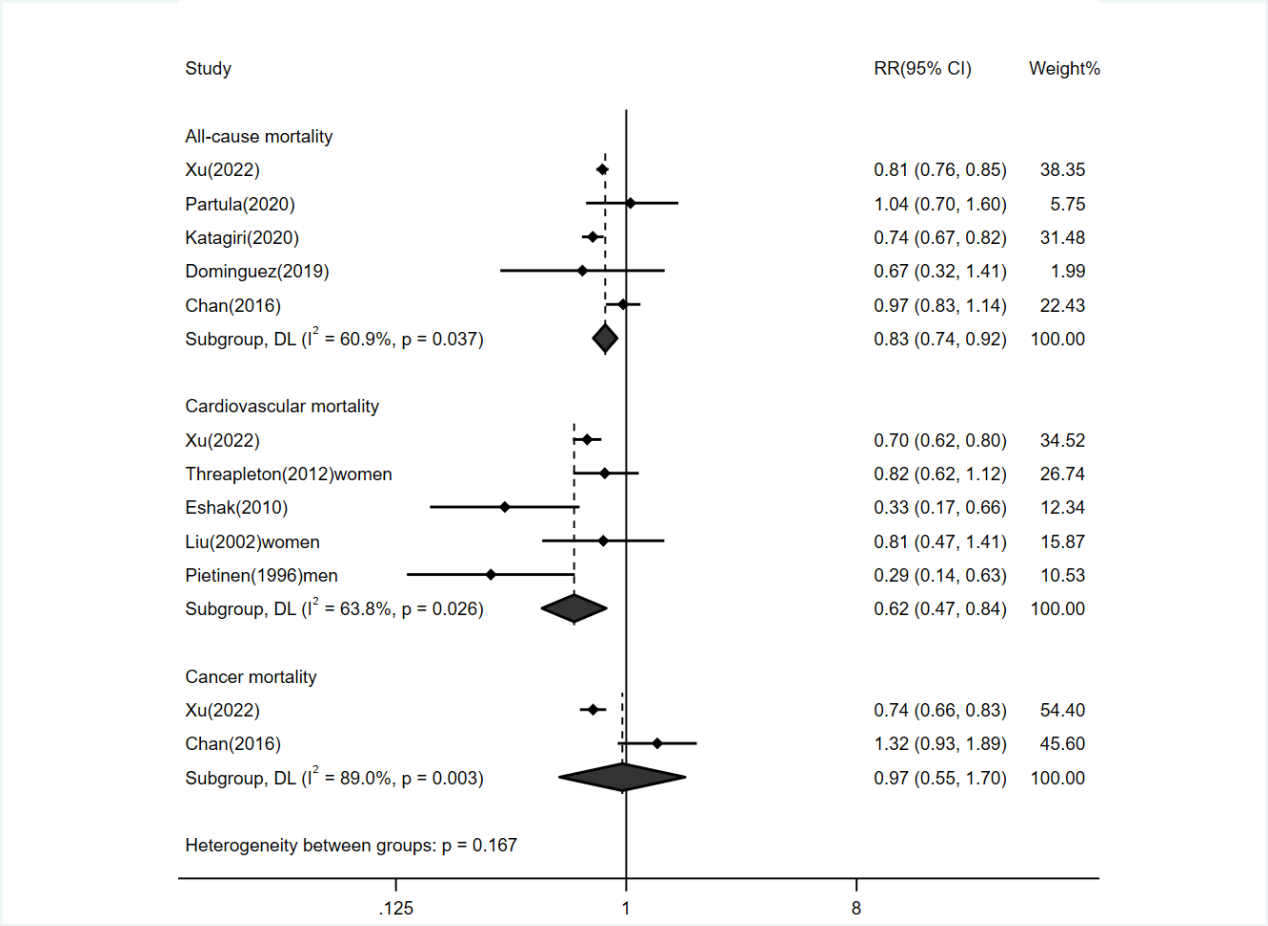


Supplemental Figure 24. Forest plots of the association between soluble fiber intake and risk of all-cause, CVD and cancer mortality. RR, relative risk, per 10 g intake.

**
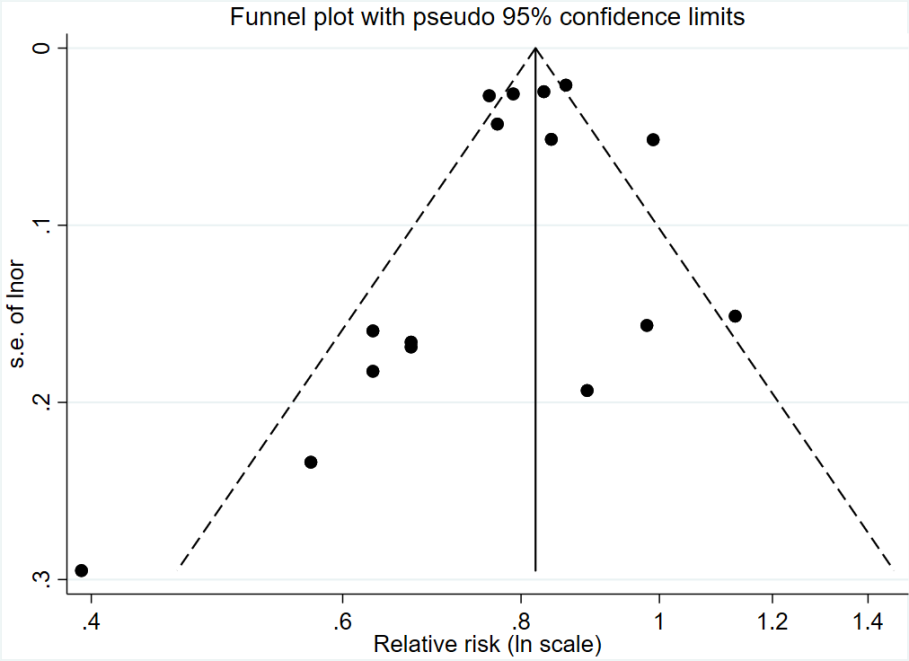
**


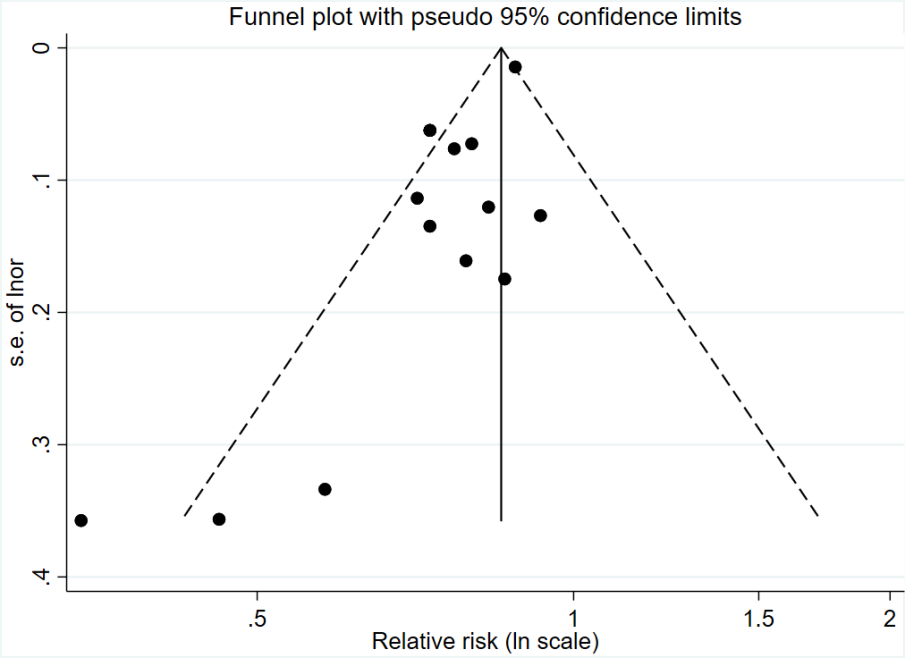


B

A


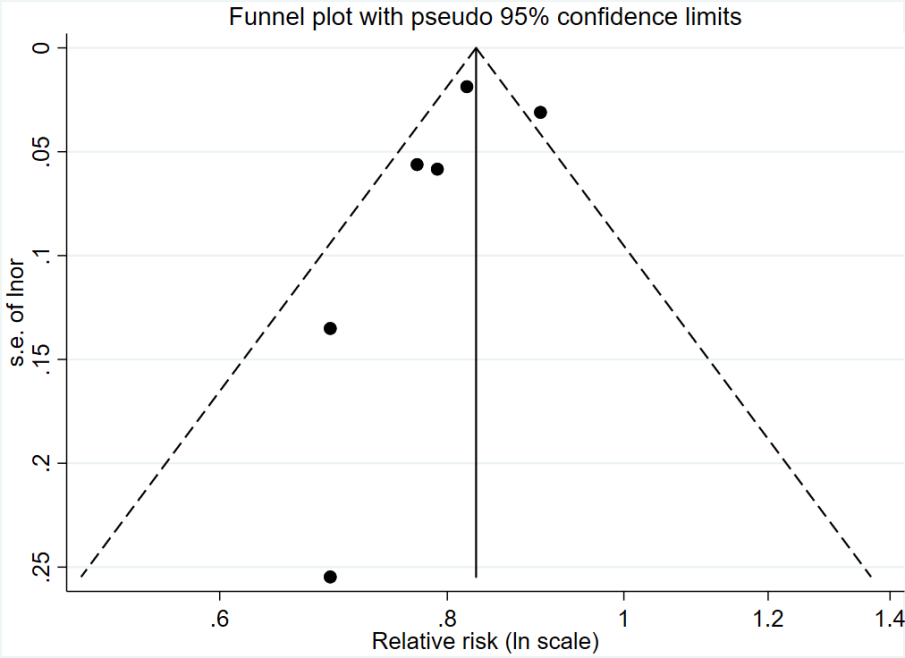


C

**Supplemental Figure 25. Funnel plots for detection of publication bias of included studies for highest vs. lowest intake meta-analysis of dietary fiber intake and risk of all-cause (A), CVD (B) and cancer(C) mortality.
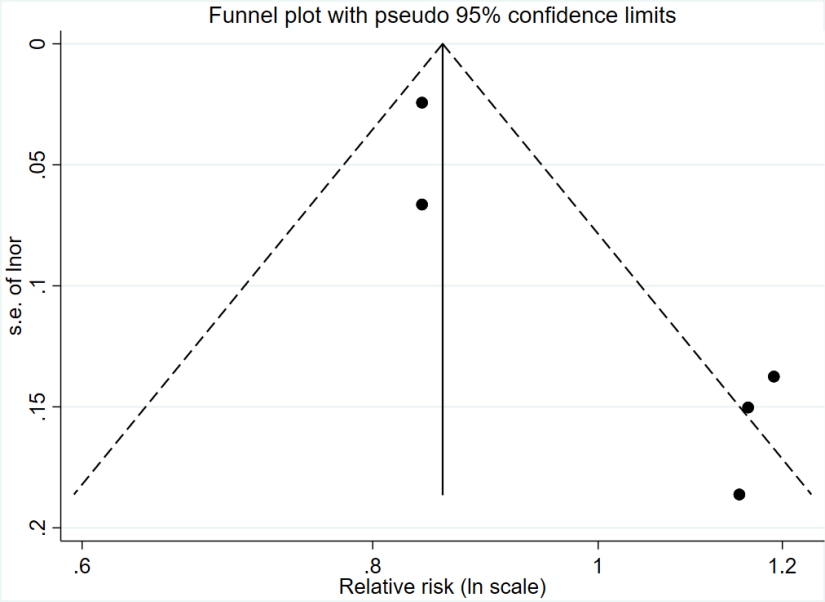
**

B

A


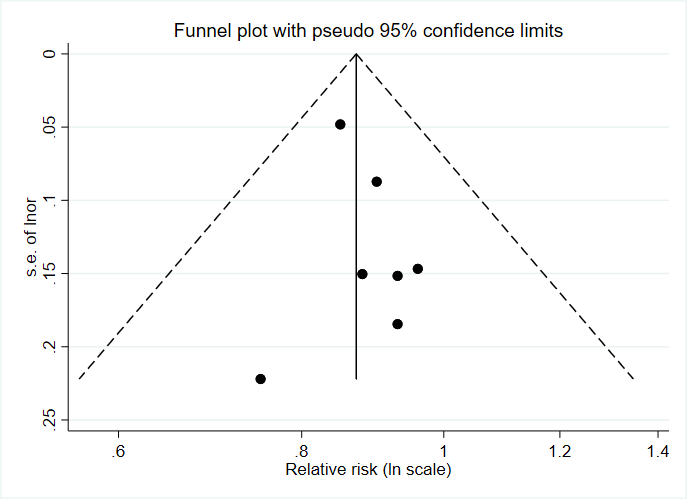


**Supplemental Figure 26. Funnel plots for detection of publication bias of included studies for highest vs. lowest intake meta-analysis of vegetable fiber intake and risk of all-cause(A), and cancer (B) mortality.**

**
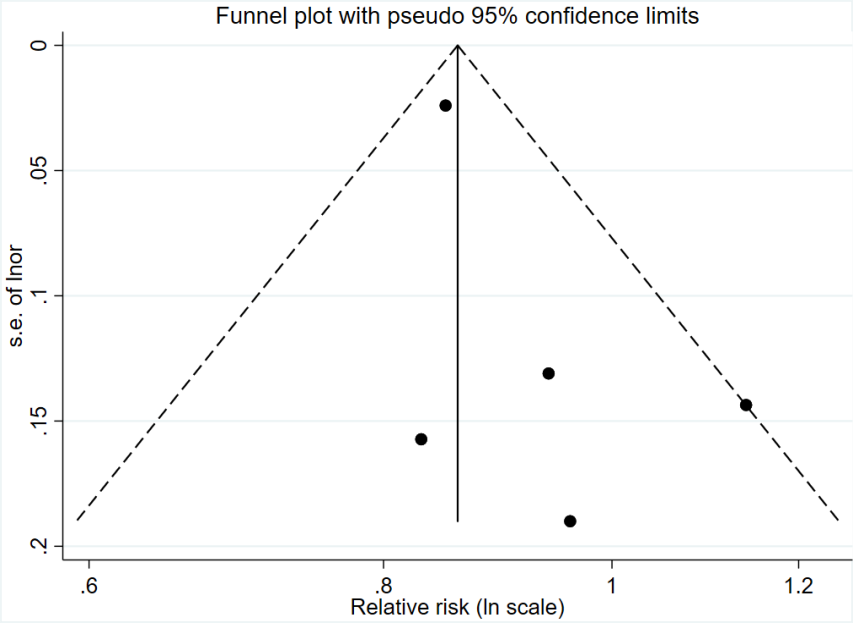
**

A

B


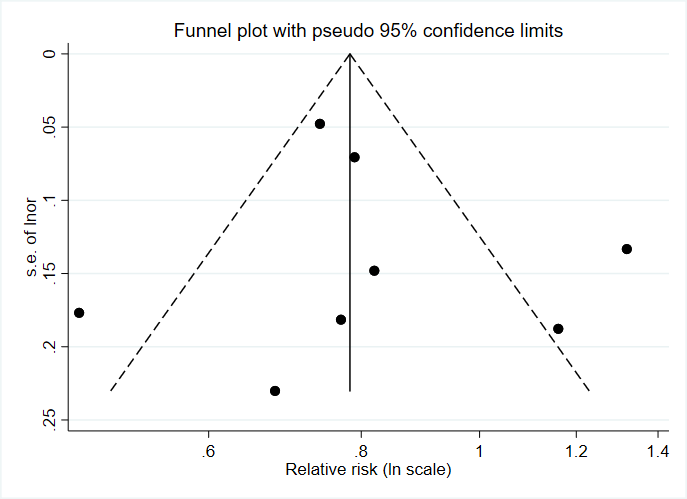


**Supplemental Figure 27. Funnel plots for detection of publication bias of included studies for highest vs. lowest intake meta-analysis of fruit fiber intake and risk of all-cause(A) and CVD (B) mortality.**

A


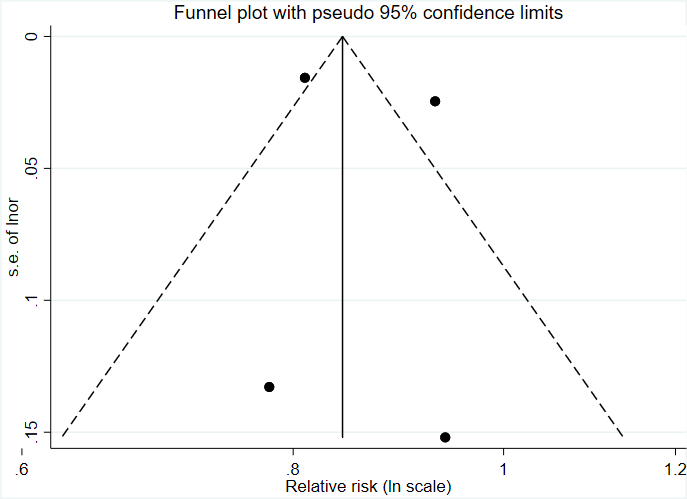


B

A


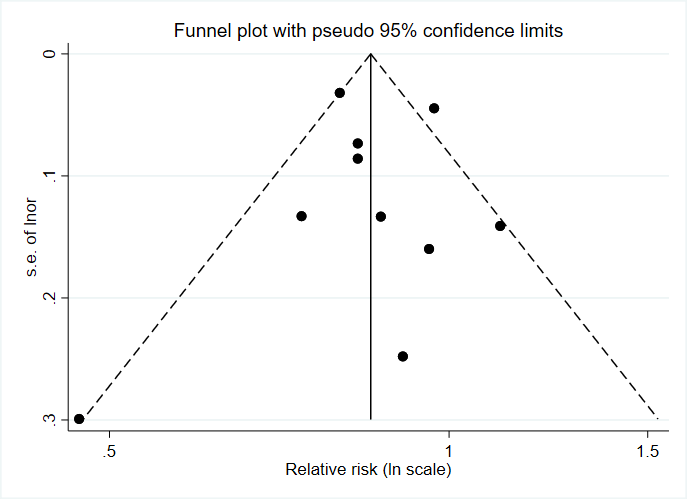


**Supplemental Figure 28. Funnel plots for detection of publication bias of included studies for highest vs. lowest intake meta-analysis of cereal fiber intake and risk of all-cause(A) and cardiovascular(B) mortality.**

**
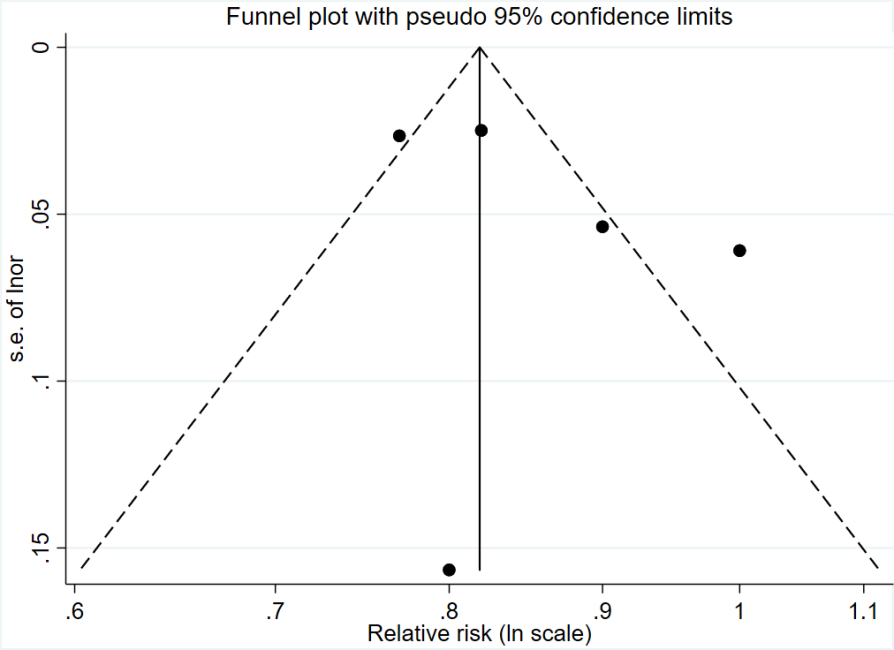
**

A

**
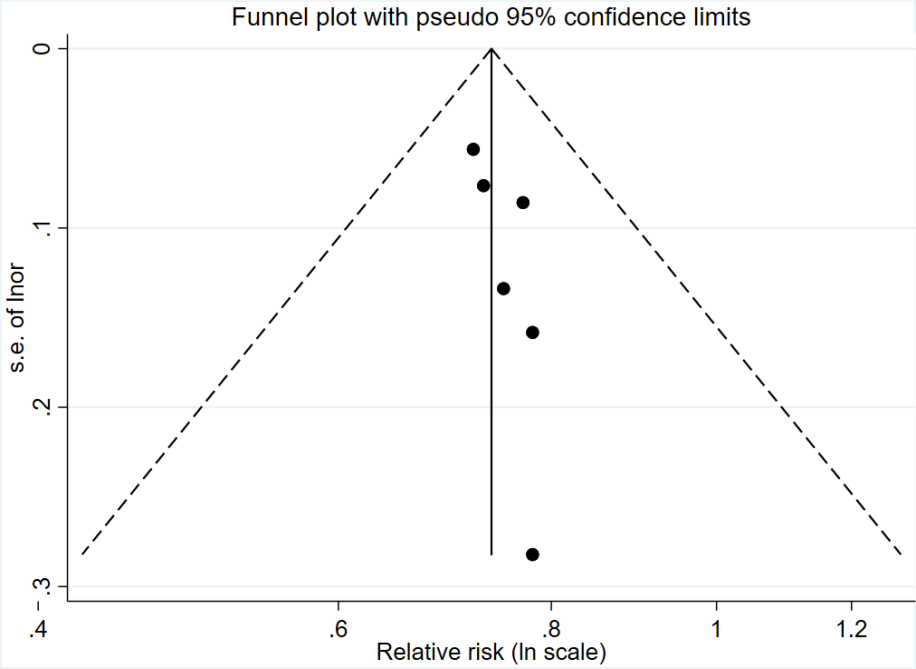
**

B

A

**Supplemental Figure 29. Funnel plots for detection of publication bias of included studies for highest vs. lowest intake meta-analysis of insoluble fiber intake and risk of all-cause(A) and cardiovascular(B) mortality.**

**
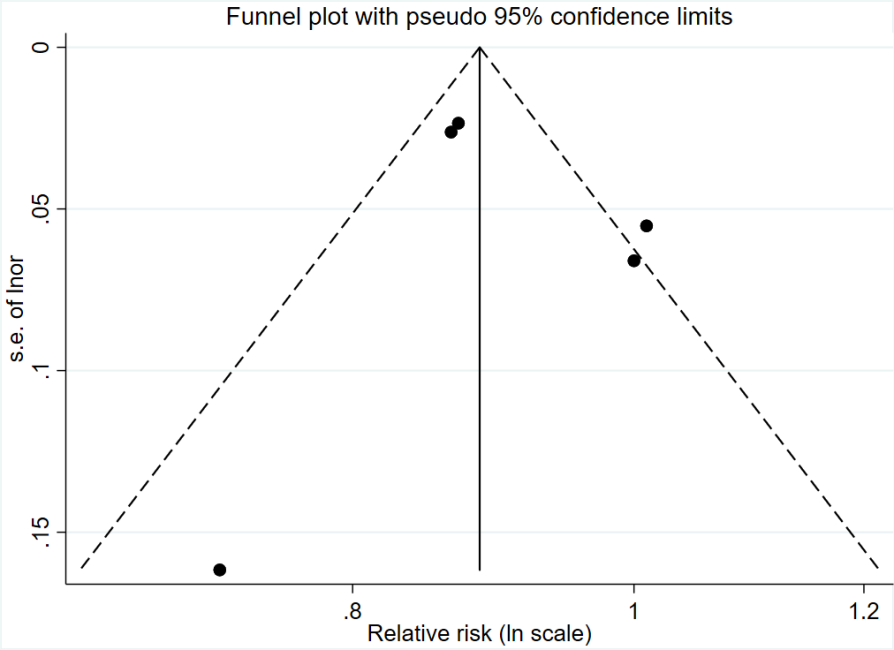
**

A

**
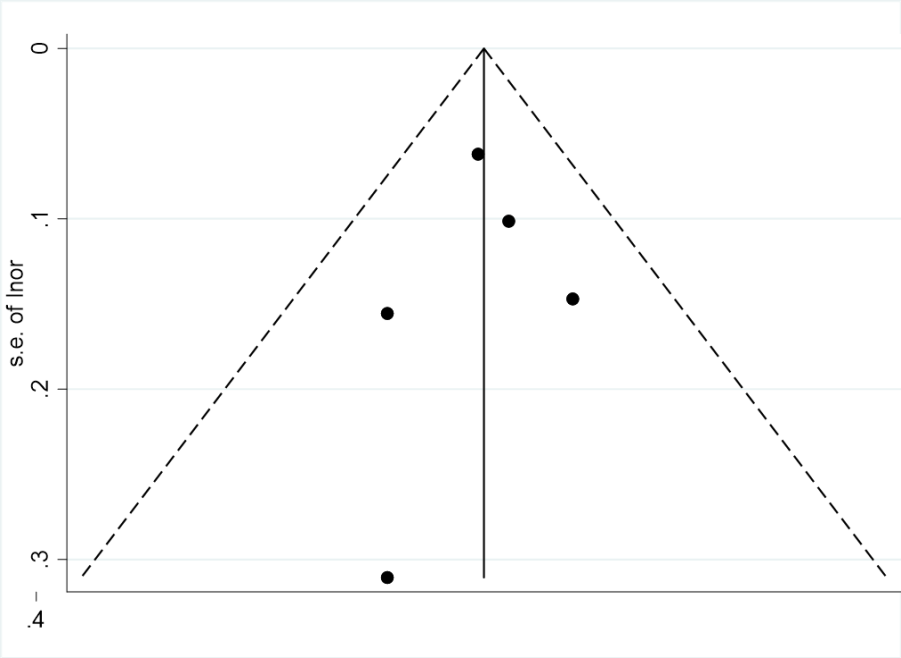
**

B

**Supplemental Figure 30. Funnel plots for detection of publication bias of included studies for highest vs. lowest intake meta-analysis of soluble fiber intake and risk of all-cause(A) and CVD B) mortality.**

**
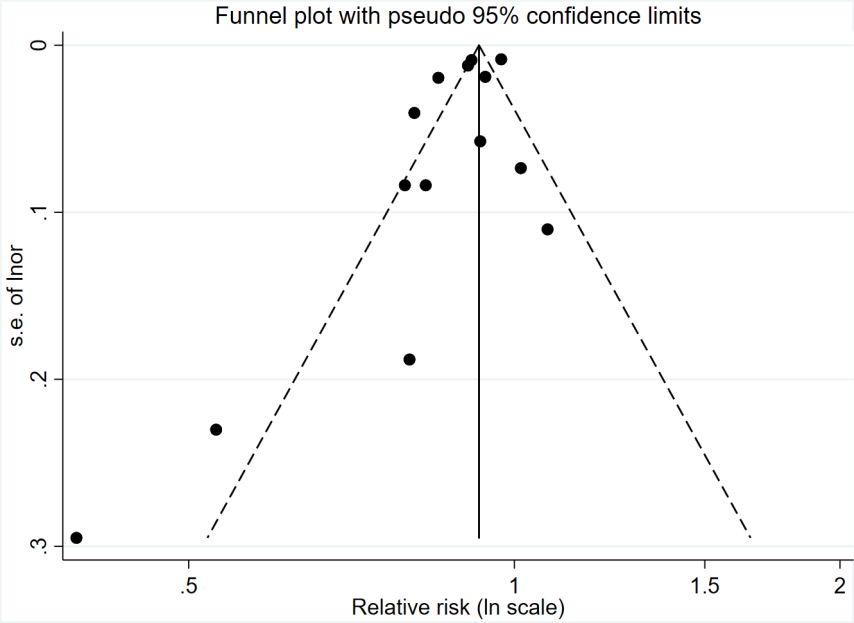
**

A

**
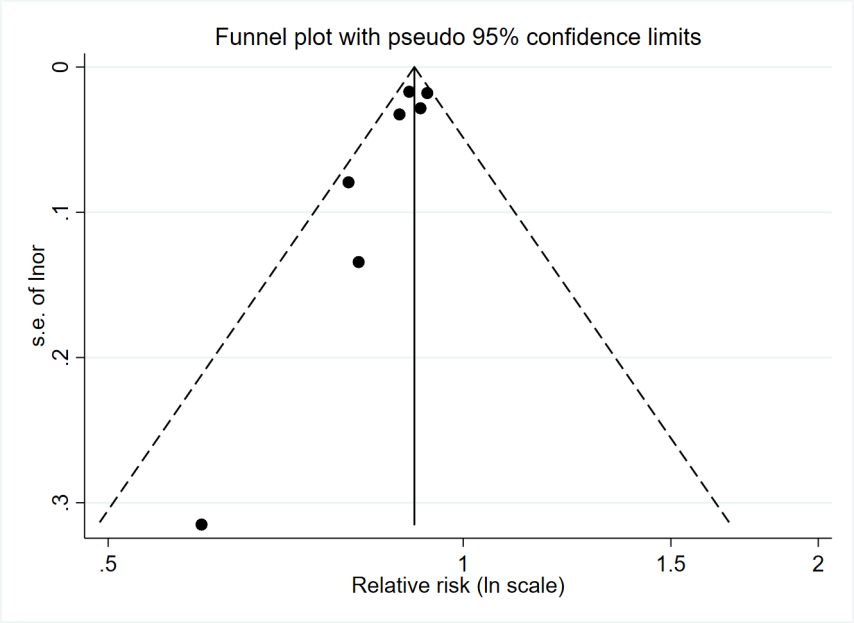
**

C

B

**
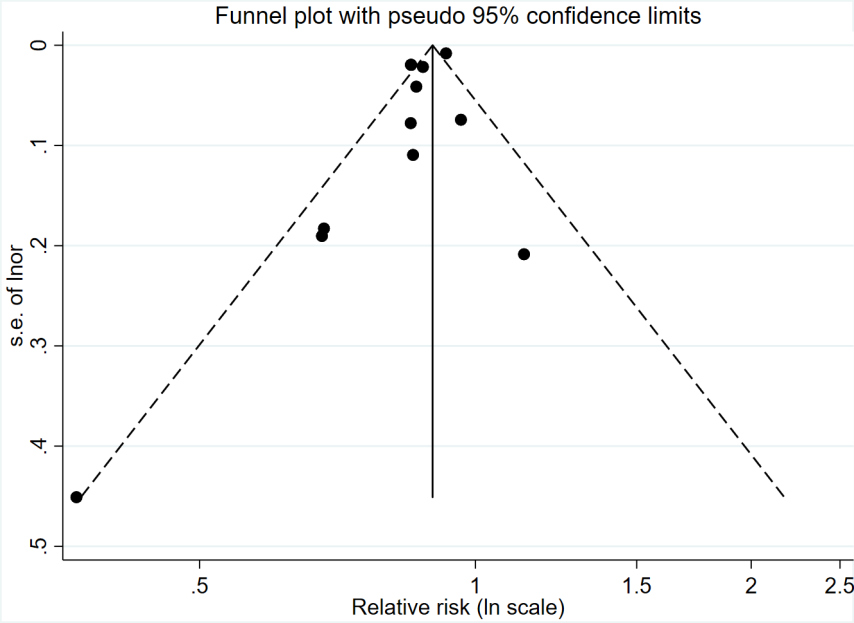
**

**Supplemental Figure 31. Funnel plots for detection of publication bias of included studies for dietary fiber intake and risk of all-cause (A), cardiovascular(B) and cancer(C) mortality, per 10 g intake.**

A


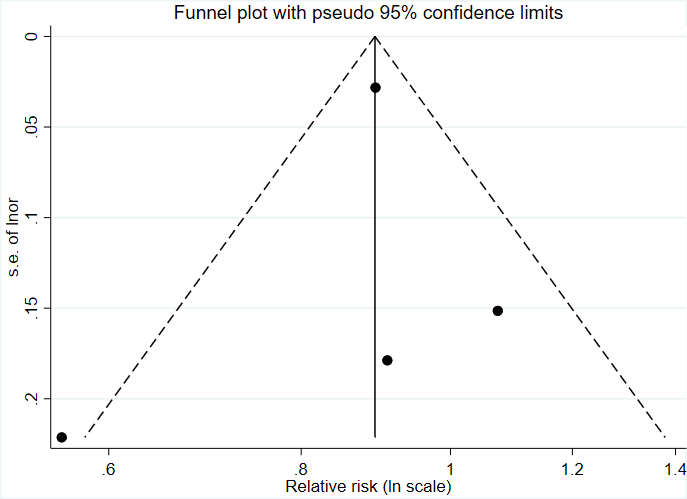


B


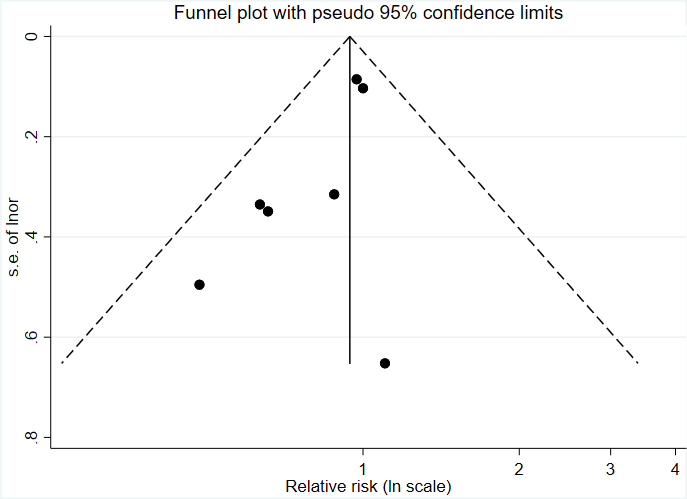


**Supplemental Figure 32. Funnel plots for detection of publication bias of included studies for vegetable fiber intake and risk of all-cause(A) and cardiovascular(B) mortality, per 10 g intake.**

A


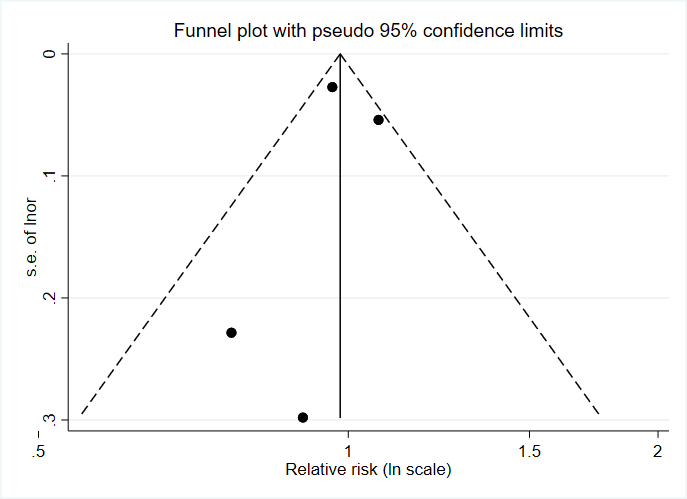

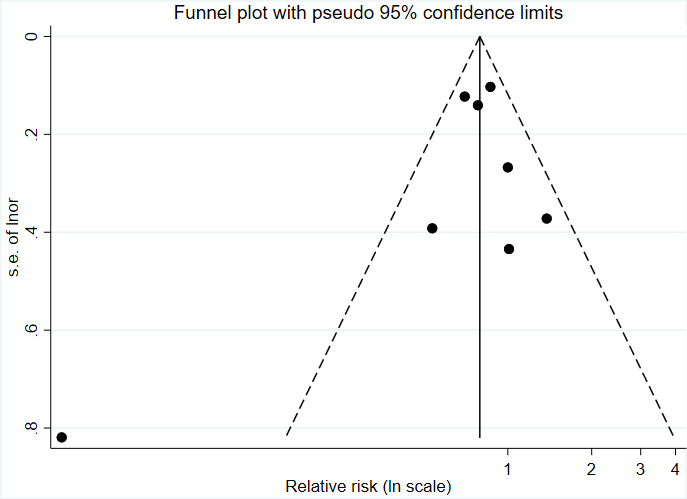


B

**Supplemental Figure 33. Funnel plots for detection of publication bias of included studies for fruit fiber intake and risk of all-cause (A) and cardiovascular (B) mortality, per 10 g intake.**

A


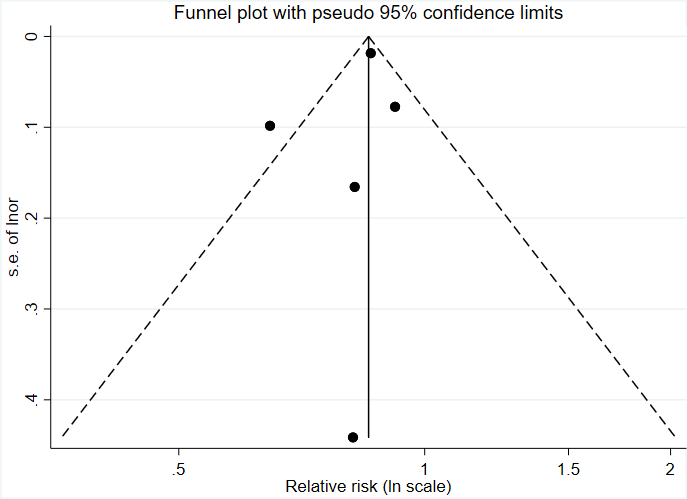


B


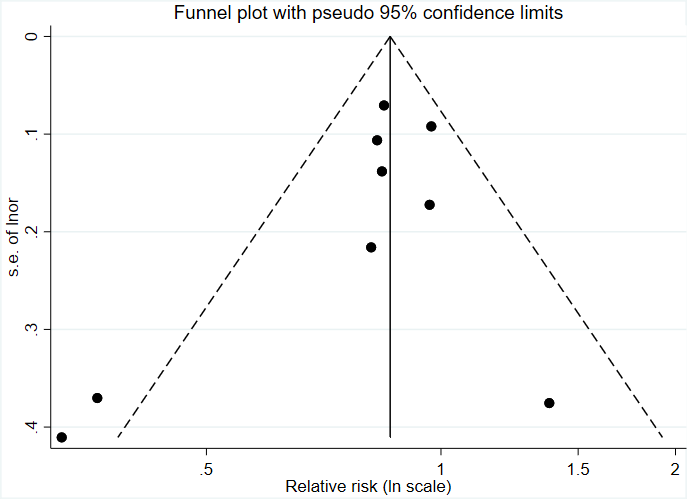


**Supplemental Figure 34. Funnel plots for detection of publication bias of included studies for cereal fiber intake and risk of all-cause(A), CVD(B) mortality, per 10 g intake.**


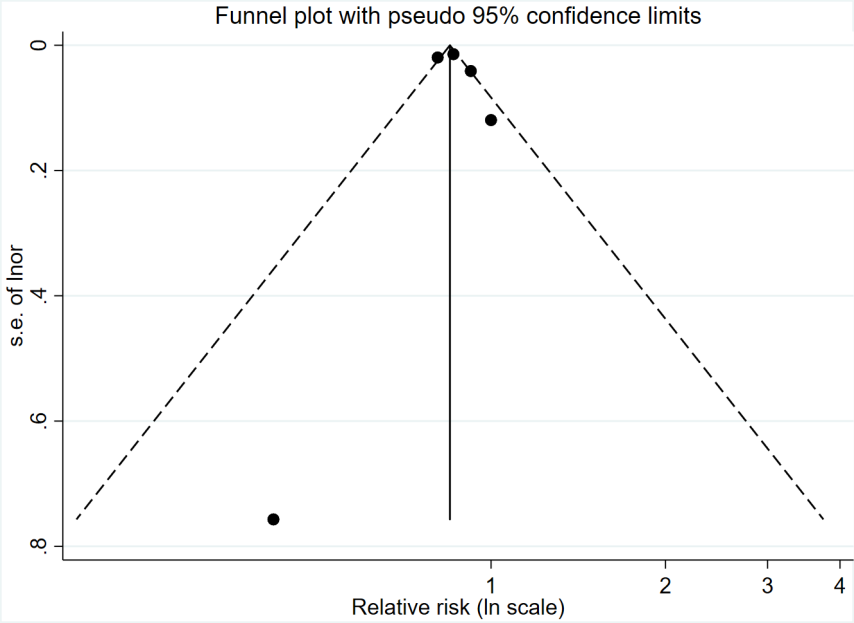


A

**
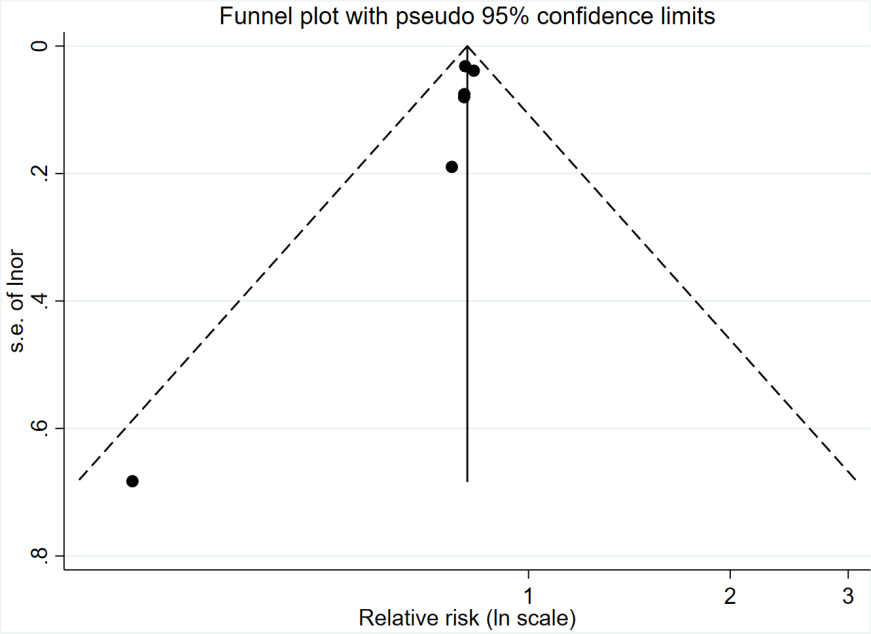
**

B

**Supplemental Figure 35. Funnel plots for detection of publication bias of included studies for insoluble fiber intake and risk of all-cause(A) and cardiovascular(B) mortality, per 10 g intake.**

**
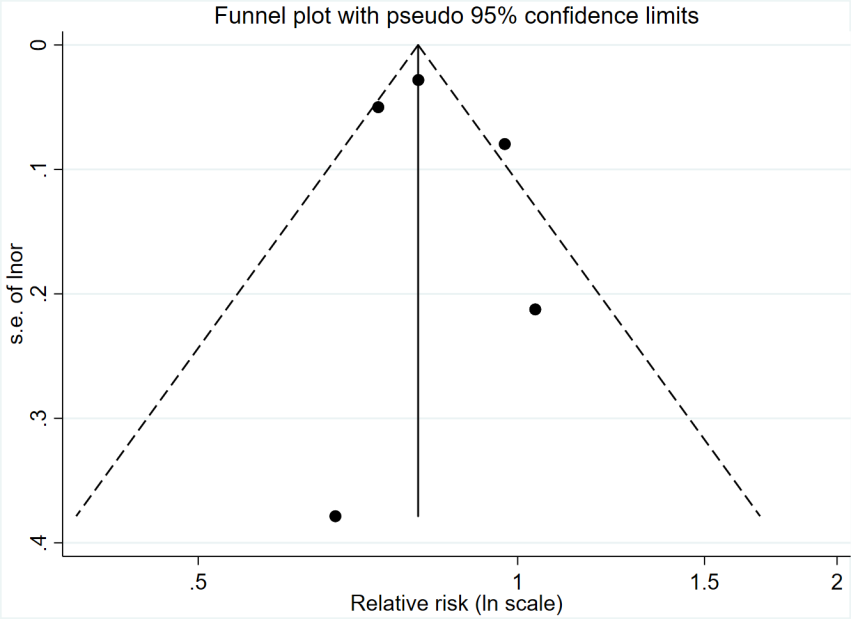
**

A

**
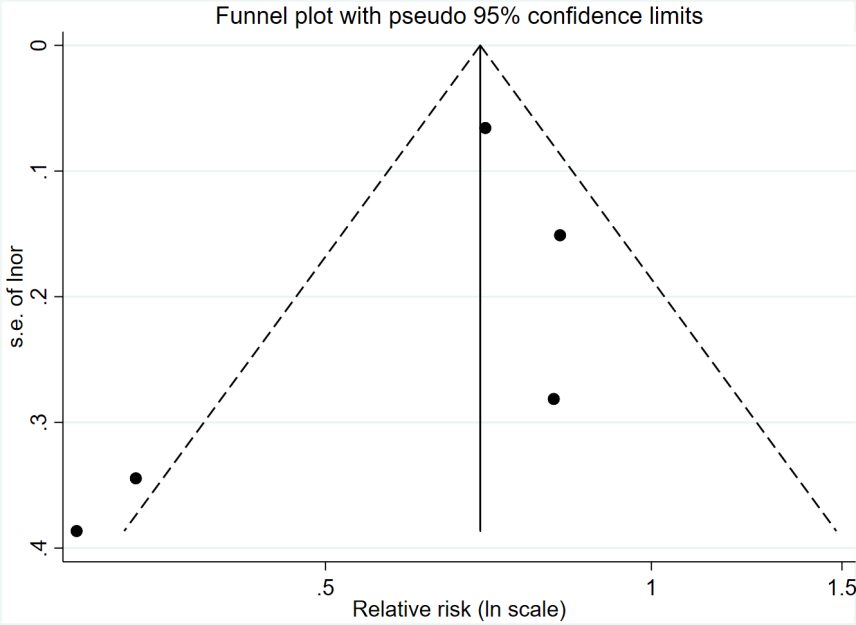
**

B

**Supplemental Figure 36. Funnel plots for detection of publication bias of included studies for soluble fiber intake and risk of all-cause(A) and cardiovascular(B) mortality, per 10 g intake.**

References:

1. Buil-Cosiales P, Zazpe I, Toledo E, Corella D, Salas-Salvadó J, Diez-Espino J, et al. Fiber intake and all-cause mortality in the Prevención con Dieta Mediterránea (PREDIMED) study. The American journal of clinical nutrition. 2014;100(6):1498-507.

2. Chuang SC, Norat T, Murphy N, Olsen A, Tjønneland A, Overvad K, et al. Fiber intake and total and cause-specific mortality in the European Prospective Investigation into Cancer and Nutrition cohort. The American journal of clinical nutrition. 2012;96(1):164-74.

3. Dominguez LJ, Bes-Rastrollo M, Toledo E, Gea A, Fresán U, Barbagallo M, et al. Dietary fiber intake and mortality in a Mediterranean population: the "Seguimiento Universidad de Navarra" (SUN) project. European journal of nutrition. 2019;58(8):3009-22.

4. Gopinath B, Flood VM, Kifley A, Louie JC, Mitchell P. Association Between Carbohydrate Nutrition and Successful Aging Over 10 Years. The journals of gerontology Series A, Biological sciences and medical sciences. 2016;71(10):1335-40.

5. Ha K, Sakaki JR, Chun OK. Nutrient Adequacy Is Associated with Reduced Mortality in US Adults. Journal of Nutrition. 2021;151(10):3214-22.

6. Ho FK, Gray SR, Welsh P, Petermann-Rocha F, Foster H, Waddell H, et al. Associations of fat and carbohydrate intake with cardiovascular disease and mortality: prospective cohort study of UK Biobank participants. Bmj-British Medical Journal. 2020;368.

7. Katagiri R, Goto A, Sawada N, Yamaji T, Iwasaki M, Noda M, et al. Dietary fiber intake and total and cause-specific mortality: the Japan Public Health Center-based prospective study. American Journal of Clinical Nutrition. 2020;111(5):1027-35.

8. Kwon YJ, Lee HS, Park GE, Lee JW. Association Between Dietary Fiber Intake and All-Cause and Cardiovascular Mortality in Middle Aged and Elderly Adults With Chronic Kidney Disease. Frontiers in nutrition. 2022;9:863391.

9. Lubin F, Lusky A, Chetrit A, Dankner R. Lifestyle and ethnicity play a role in all-cause mortality. Journal of Nutrition. 2003;133(4):1180-5.

10. Partula V, Deschasaux M, Druesne-Pecollo N, Latino-Martel P, Desmetz E, Chazelas E, et al. Associations between consumption of dietary fibers and the risk of cardiovascular diseases, cancers, type 2 diabetes, and mortality in the prospective NutriNet-Sante cohort. American Journal of Clinical Nutrition. 2020;112(1):195-207.

11. Todd S, Woodward M, Tunstall-Pedoe H, Bolton-Smith C. Dietary antioxidant vitamins and fiber in the etiology of cardiovascular disease and all-causes mortality: Results from the Scottish Heart Health Study. American journal of epidemiology. 1999;150(10):1073-80.

12. Xu M. Ready to eat cereal consumption with total and cause-specific mortality: Prospective analysis of 367,442 individuals. 2016.

13. Nilsson LM, Winkvist A, Brustad M, Jansson JH, Johansson I, Lenner P, et al. A traditional Sami diet score as a determinant of mortality in a general northern Swedish population. 2012.

14. Zhang Z, Chen B, Zeng J, Fan M, Xu W, Li X, et al. Associations between Consumption of Dietary Fibers and the Risk of Type 2 Diabetes, Hypertension, Obesity, Cardiovascular Diseases, and Mortality in Chinese Adults: Longitudinal Analyses from the China Health and Nutrition Survey. Nutrients. 2022;14(13).

15. You YX, Rivan NFM, Singh DKA, Rajab NF, Ludin AFM, Din NC, et al. Incidence and Predictors of Mortality among Community-Dwelling Older Adults in Malaysia: A 5 Years Longitudinal Study. International journal of environmental research and public health. 2022;19(15).

16. Xu X, Zhang J, Zhang Y, Qi H, Wang P. Associations between dietary fiber intake and mortality from all causes, cardiovascular disease and cancer: a prospective study. Journal of translational medicine. 2022;20(1):344.

17. Crowe FL, Key TJ, Appleby PN, Overvad K, Schmidt EB, Egeberg R, et al. Dietary fibre intake and ischaemic heart disease mortality: the European Prospective Investigation into Cancer and Nutrition-Heart study. European journal of clinical nutrition. 2012;66(8):950-6.

18. Eshak ES, Iso H, Date C, Kikuchi S, Watanabe Y, Wada Y, et al. Dietary Fiber Intake Is Associated with Reduced Risk of Mortality from Cardiovascular Disease among Japanese Men and Women. Journal of Nutrition. 2010;140(8):1445-53.

19. Khaw KT, Barrett-Connor E. Dietary fiber and reduced ischemic heart disease mortality rates in men and women: a 12-year prospective study. American journal of epidemiology. 1987;126(6):1093-102.

20. Miyazawa I, Miura K, Miyagawa N, Kondo K, Kadota A, Okuda N, et al. Relationship between carbohydrate and dietary fibre intake and the risk of cardiovascular disease mortality in Japanese: 24-year follow-up of NIPPON DATA80. European journal of clinical nutrition. 2020;74(1):67-76.

21. Pietinen P, Ascherio A, Korhonen P, Hartman AM, Willett WC, Albanes D, et al. Intake of fatty acids and risk of coronary heart disease in a cohort of Finnish men. The Alpha-Tocopherol, Beta-Carotene Cancer Prevention Study. American journal of epidemiology. 1996;145(10):876-87.

22. Xu H, Huang XY, Riserus U, Krishnamurthy VM, Cederholm T, Arnlov J, et al. Dietary Fiber, Kidney Function, Inflammation, and Mortality Risk. Clinical Journal of the American Society of Nephrology. 2014;9(12):2104-10.

23. Buyken AE, Flood V, Empson M, Rochtchina E, Barclay AW, Brand-Miller J, et al. Carbohydrate nutrition and inflammatory disease mortality in older adults. American Journal of Clinical Nutrition. 2010;92(3):634-43.

24. Liu SM, Buring JE, Sesso HD, Rimm EB, Willett WC, Manson JE. A prospective study of dietary fiber intake and risk of cardiovascular disease among women. Journal of the American College of Cardiology. 2002;39(1):49-56.

25. Threapleton DE, Greenwood DC, Burley VJ, Aldwairji M, Cade JE. Dietary fibre and cardiovascular disease mortality in the UK Women's Cohort Study. European journal of epidemiology. 2012;28(4):335-46.

26. Mozaffarian D, Kumanyika SK, Lemaitre RN, Olson JL, Burke GL, Siscovick DS. Cereal, fruit, and vegetable fiber intake and the risk of cardiovascular disease in elderly individuals. Jama-Journal of the American Medical Association. 2003;289(13):1659-66.

27. Huang T, Xu M, Lee A, Cho S, Qi L. Consumption of whole grains and cereal fiber and total and cause-specific mortality: prospective analysis of 367,442 individuals (vol 13, pg 59, 2015). BMC medicine. 2015;13.

28. Kaushik S, Wang JJ, Wong TY, Flood V, Barclay A, Brand-Miller J, et al. Glycemic Index, Retinal Vascular Caliber, and Stroke Mortality. Stroke. 2009;40(1):206-12.

29. Chan CW, Lee PH. Association between dietary fibre intake with cancer and all-cause mortality among 15 740 adults: the National Health and Nutrition Examination Survey III. Journal of Human Nutrition and Dietetics. 2016;29(5):633-42.

Women. Journal of Nutrition. 2010;140(8):1445-53.

19. Khaw KT, Barrett-Connor E. Dietary fiber and reduced ischemic heart disease mortality rates in men and women: a 12-year prospective study. American journal of epidemiology. 1987;126(6):1093-102.

20. Miyazawa I, Miura K, Miyagawa N, Kondo K, Kadota A, Okuda N, et al. Relationship between carbohydrate and dietary fibre intake and the risk of cardiovascular disease mortality in Japanese: 24-year follow-up of NIPPON DATA80. European journal of clinical nutrition. 2020;74(1):67-76.

21. Pietinen P, Ascherio A, Korhonen P, Hartman AM, Willett WC, Albanes D, et al. Intake of fatty acids and risk of coronary heart disease in a cohort of Finnish men. The Alpha-Tocopherol, Beta-Carotene Cancer Prevention Study. American journal of epidemiology. 1996;145(10):876-87.

22. Xu H, Huang XY, Riserus U, Krishnamurthy VM, Cederholm T, Arnlov J, et al. Dietary Fiber, Kidney Function, Inflammation, and Mortality Risk. Clinical Journal of the American Society of Nephrology. 2014;9(12):2104-10.
